# Computational prediction of miRNAs and their targets in *Phaseolus vulgaris* using simple sequence repeat signatures

**DOI:** 10.1186/s12870-015-0516-3

**Published:** 2015-06-12

**Authors:** Chandran Nithin, Nisha Patwa, Amal Thomas, Ranjit Prasad Bahadur, Jolly Basak

**Affiliations:** Computational Structural Biology Lab, Department of Biotechnology, Indian Institute of Technology Kharagpur, Kharagpur, 721302 India; Department of Biotechnology, Visva-Bharati, Santiniketan, 731235 India

**Keywords:** miRNA, *Phaseolus vulgaris*, SSRs, Shannon entropy, MFEI

## Abstract

**Background:**

MicroRNAs (miRNAs) are endogenous, noncoding, short RNAs directly involved in regulating gene expression at the post-transcriptional level. In spite of immense importance, limited information of *P. vulgaris* miRNAs and their expression patterns prompted us to identify new miRNAs in *P. vulgaris* by computational methods. Besides conventional approaches, we have used the simple sequence repeat (SSR) signatures as one of the prediction parameter. Moreover, for all other parameters including normalized Shannon entropy, normalized base pairing index and normalized base-pair distance, instead of taking a fixed cut-off value, we have used 99 % probability range derived from the available data.

**Results:**

We have identified 208 mature miRNAs in *P. vulgaris* belonging to 118 families, of which 201 are novel. 97 of the predicted miRNAs in *P. vulgaris* were validated with the sequencing data obtained from the small RNA sequencing of *P. vulgaris.* Randomly selected predicted miRNAs were also validated using qRT-PCR. A total of 1305 target sequences were identified for 130 predicted miRNAs. Using 80 % sequence identity cut-off, proteins coded by 563 targets were identified. The computational method developed in this study was also validated by predicting 229 miRNAs of *A. thaliana* and 462 miRNAs of *G. max,* of which 213 for *A. thaliana* and 397 for *G. max* are existing in miRBase 20.

**Conclusions:**

There is no universal SSR that is conserved among all precursors of Viridiplantae, but conserved SSR exists within a miRNA family and is used as a signature in our prediction method. Prediction of known miRNAs of *A. thaliana* and *G. max* validates the accuracy of our method. Our findings will contribute to the present knowledge of miRNAs and their targets in *P. vulgaris.* This computational method can be applied to any species of Viridiplantae for the successful prediction of miRNAs and their targets.

**Electronic supplementary material:**

The online version of this article (doi:10.1186/s12870-015-0516-3) contains supplementary material, which is available to authorized users.

## Background

MicroRNAs (miRNAs) are small non-coding RNAs [1] with an approximate length of 22 nucleotides originating from long self-complementary precursors [2]. miRNA precursor sequences (pre-miRs) have intrinsic hairpin structure which consists of the entire miRNA sequence on one arm of the hairpin and the miRNA* sequence on the opposite arm. miRNAs regulate a variety of biological processes like development, metabolism, stress response, pathogen defense and maintenance of genome integrity [3, 4]. Mature miRNA gets incorporated into the RNA-induced silencing complex (RISC) [2], which regulates gene expression either by inhibiting translation or by degrading coding mRNAs by perfect or near-perfect complement with the target mRNAs [5, 6]. For a given miRNA, the number of target mRNA ranges from one to hundreds [7]. However, in plants, most of the target mRNAs contain a single miRNA-complementary site, and the corresponding miRNAs perfectly complement these sites and cleave the target mRNAs [8].

The first miRNA (lin-4) was identified in *Caenorhabditis elegans* in 1993 [9]. Since then, hundreds of miRNAs have been identified in plants, animals and viruses. In recent years, advancement in technologies such as Bioinformatics and Next-Generation Sequencing (NGS) facilitated the identification of huge number of putative miRNAs in different organisms. However, the process of identifying miRNAs is still a complex and difficult task requiring interdisciplinary strategies, including experimental approaches as well as computational methods. Compared to the experimental approaches, computational predictions have been proved to be fast, affordable, and accurate [10–26]. In the last ten years, different computational strategies have been developed to find new miRNAs, including mining the repository of available Expressed Sequence Tags (ESTs) with known miRNAs, as well as those based on the conserved nature of miRNAs [12–16, 22, 23].

Majority of miRNAs are evolutionarily conserved between different species of the same kingdom and may also exist as orthologs or homologs in other species [27]. Computational prediction of putative miRNAs is often based on their evolutionarily conserved nature. Accordingly, homologs of known miRNAs are searched in the EST databases to identify the putative pre-miRs in other species. Pre-miRs have a specific range of percentage AU content in their sequences as well as Minimal Folding free Energy Index (MFEI) [27]. Studies have also shown that pre-miRs have distinct RNA folding measures such as normalised Shannon entropy (NQ), normalized base-pair distance (ND) and normalized base-pairing propensity (Npb). Thus, AU content and MFEI are also used as parameters for prediction of new miRNAs.

Simple sequence repeats (SSRs) are repeating sequences of one to six nucleotides long [28]. The presence of SSRs in pre-miRs was identified by several studies [29–31], although their precise role in pre-miRs is yet to be elucidated. The SSRs present in pre-miRs in different species did not show noticeable locational preferences and are found anywhere in pre-miRs, suggesting that SSRs are the important component of pre-miRs [32]. In pre-miRs, mononucleotide repeats are the most abundant repeats, followed by di- and tri-nucleotide repeats, while tetra-, penta-, and hexanucleotide repeats rarely occur [32]. Moreover, the number of repeats correlates inversely to the length of the repeats [32]. Absence of long SSRs and low number of repeat types in pre-miRNAs may be attributed to their small size, stability and low mutation rate [32]. Due to these very characteristics, the identification of SSR signatures in pre-miRs is easy and can be used as a parameter in predicting miRNAs. However, SSR signatures have not been used in the computational prediction of new miRNAs. In the present study, we have used SSR signatures as a parameter to predict new miRNAs.

*Phaseolus vulgaris,* belonging to the Fabaceae family*,* is a vital leguminous crop in tropical and subtropical areas of Asia, Africa, and Latin America, as well as parts of southern Europe and the USA (FAOSTAT 2009). *P. vulgaris* is an important food worldwide and a significant source of fibre, proteins and vitamins (FAOSTAT 2009). High protein and carbohydrate content makes it not only important for the human diet, but also suitable as high protein feed and fodder for livestock. *P. vulgaris* is a particular valuable component of low-input farming system of resource-poor farmers (FAOSTAT 2009). This leguminous crop enhances soil fertility through nitrogen fixation [33]. In spite of immense importance, limited information is available about the miRNAs of *P. vulgaris* and their patterns of expression [34–40]. There are only eight reported miRNAs of *P. vulgaris* in the miRBase 20 [41]. In the present study, we have identified new miRNAs *in P. vulgaris* by computational methods. In addition to the conventional approaches, we have used the conserved SSR signatures as one of the parameters for prediction. Moreover, for all the other parameters, instead of considering a fixed cut-off value, we have used a 99 % probability range derived from the available data. We obtained 208 new miRNAs, of which 201 are novel. Few randomly selected predicted miRNAs were validated using qRT-PCR. Targets for many of the predicted miRNAs were identified. Additionally, we also validated our computational method by predicting known miRNAs in *A. thaliana* and *G. max.* Our findings will contribute to the present knowledge of miRNAs and their targets in *P. vulgaris*. The computational method developed in this study is not only restricted to *P. vulgaris* but can be applied to any species of Viridiplantae.

## Results

### Analysis of known Viridiplantae pre-miRs

**Fig. 1:**
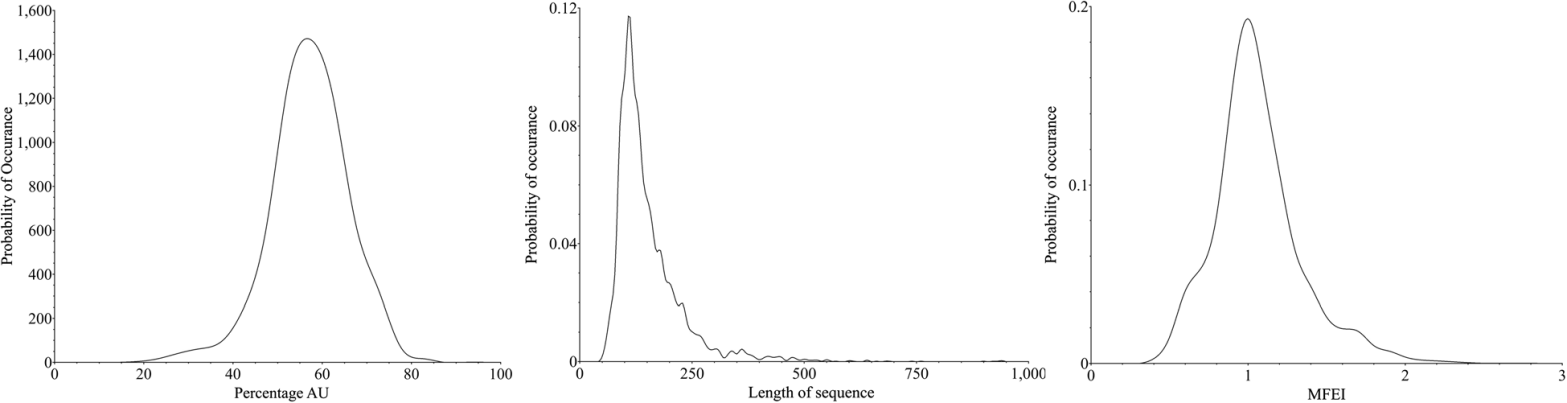
Probability distributions of percentage AU content, length and MFEI of pre-miRs belonging to Viridiplantae

**Fig. 2:**
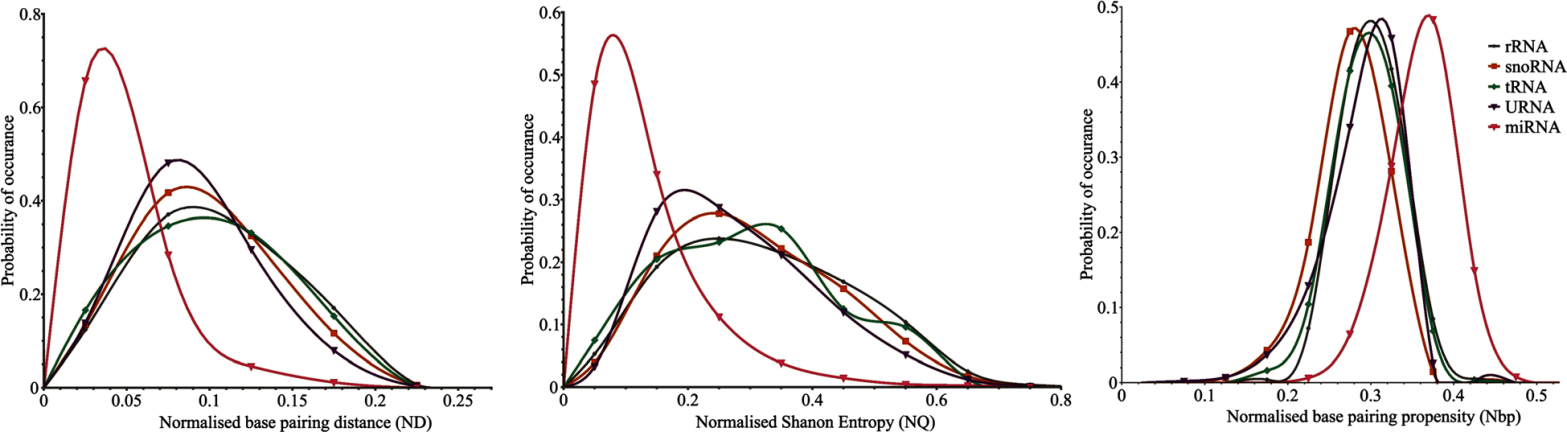
Probability distributions of normalized base-pair distance (ND). normalized Shannon entropy (NQ) and normalized base pairing propensity (Npb) of pre-miRs belonging to Viridiplantae

All the known 6088 pre-miRs of Viridiplantae in the miRBase 20 [41] were analysed, and the probability distributions of their AU content, length and MFEI are shown in Fig. 1. The length of pre-miRs varies from 43 to 938 nucleotides, with the mean value of 149. However, when we consider the 99 % probability range, the length of pre-miRs varies from 55 to 505 nucleotides. Consequently, we set this range as a cut-off value for the prediction of new miRNAs. The percentage of AU content in the pre-miRs ranges from 17 % to 92 %. This range becomes 27 % to 77 % when we consider the 99 % probability region, and accordingly it is used as the AU content cut-off range. The MFEI has a mean value of 1.0 ± 0.28, however while considering 99 % probability range, it is greater than or equal to 0.41. Consequently, this value is used as the cut-off for MFEI. The probability distributions for ND, NQ and Npb are plotted in Fig. 2. Considering the 99 % probability region in the distribution, the values of NQ and ND are less than or equal to 0.45 and 0.15, respectively, while for Npb it is greater than or equal to 0.25. These values have been used as the cut-off for these parameters.

### Simple Sequence Repeats (SSRs)

To find the conserved SSR signatures within the pre-miRs, all the 1892 miRNA families of Viridiplantae were analysed (Additional file 1 Table S1). None of the SSR signatures were found to be conserved in all the families. However, conserved SSR signature(s) was found when a particular family was considered. We find 1427 families with only one pre-miR, and 465 families with two or more pre-miRs. Within these 465 families, only those conserved SSRs that are present in all the members of a particular family were considered. The conserved SSR having the maximum average R (number of SSR signatures per 100 nucleotides) value was chosen as a SSR signature for a given family. We find that with the window size three, the average R of a signature SSR is greater than 2.5. With the increase in the window size, the number of miRNA families having a conserved SSR signature with an average R greater than two becomes limited. Accordingly, the window size three was set to identify the conserved SSR signatures in pre-miRs. For the 1427 families with only one pre-miR, the SSR with the maximum R was selected as a signature. In single member families, the R is always greater than 2.5, which is the minimum average R for the SSR signatures found in the multimember families.

**Fig. 3:**
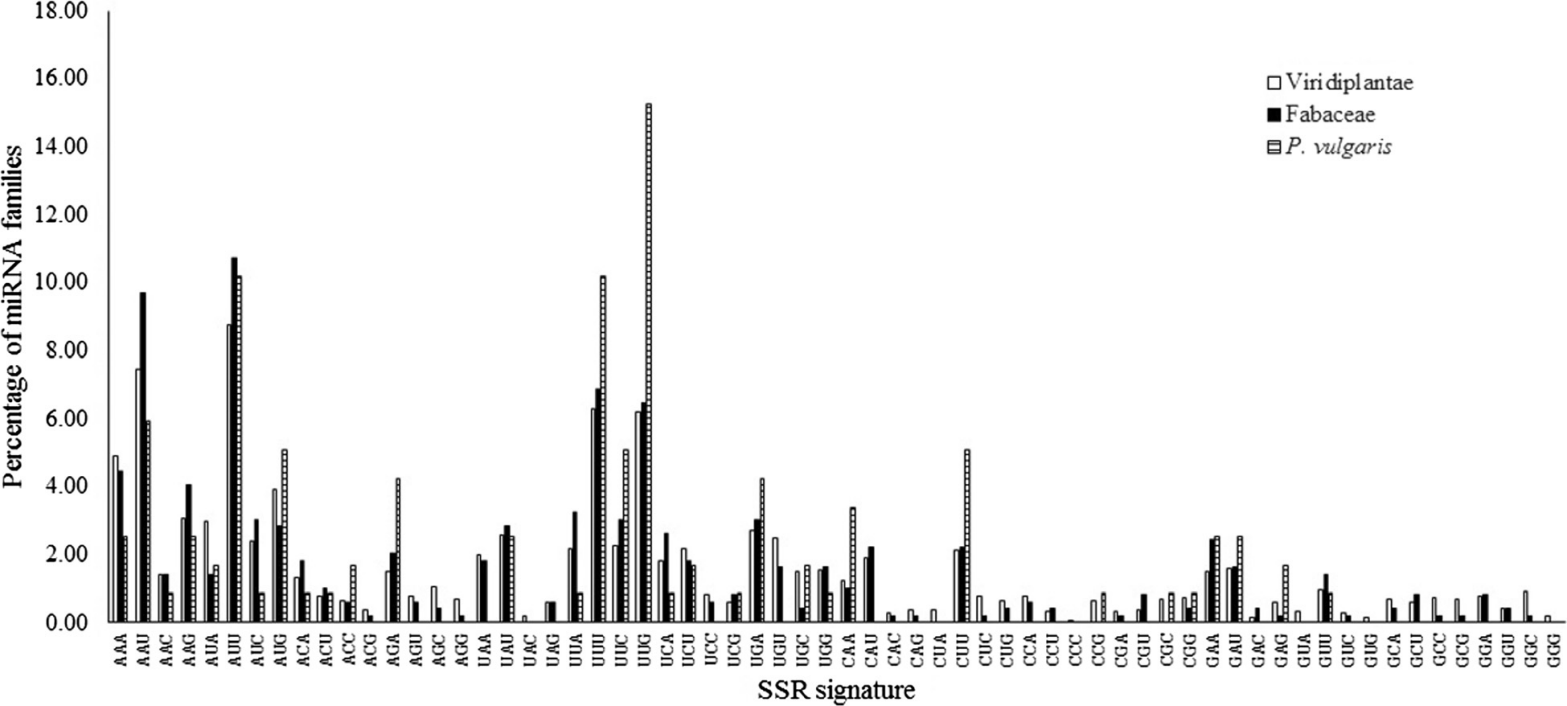
Distribution of SSR signatures in Viridiplantae, Fabaceae and *P. vulgaris*

The SSR signatures in different miRNA families of the kingdom Viridiplantae, the family Fabaceae and the species *P. vulgaris* were analysed in Table 1. It shows that in Viridiplantae, 8.77 % of miRNA families contain the signature AUU, 7.45 % of miRNA families contain the signature AAU and 6.29 % of miRNA families contain the signature UUU. In Fabaceae, 10.71 % of miRNA families contain the signature AUU, 9.70 % of miRNA families contain the signature AAU and 6.87 % of miRNA families contain the signature UUU. In *P. vulgaris*, the signature UUG is present in 15.25 % of miRNA families, while both the signatures AUU and UUU are present in 10.17 % of miRNA families. Significantly, the three most frequently found signatures in each taxonomic category are found in most of the miRNA families. They are the signatures of 23 % miRNA families in Viridiplantae, of 27 % miRNA families in Fabaceae and of 36 % miRNA families in *P. vulgaris*. The signature CCC is found in only one miRNA family in Viridiplantae, and is absent in all miRNA families in Fabaceae as well as in *P. vulgaris*. In Fabaceae, eight signatures are absent in all miRNA families, while 11 signatures are found only in one miRNA family. In *P. vulgaris,* 32 out of 64 signatures are absent in all miRNA families*.* The relative distribution of the SSR signatures in the Viridiplantae, Fabaceae and *P. vulgari*s is shown in Fig. 3.

**Table 1:** Distribution of SSR signatures in various miRNA families of Viridiplantae, Fabaceae and P. vulgaris

	A			U			C			G			
	V^a^	F^b^	P^c^	V^a^	F^b^	P^c^	V^a^	F^b^	P^c^	V^a^	F^b^	P^c^	
A	4.92	4.44	2.54	2.96	1.41	1.69	1.32	1.82	0.85	1.48	2.02	4.24	A
	7.45	9.70	5.93	8.77	10.71	10.17	0.79	1.01	0.85	0.79	0.61	0.00	U
	1.43	1.41	0.85	2.38	3.03	0.85	0.63	0.61	1.69	1.06	0.40	0.00	C
	3.07	4.04	2.54	3.91	2.83	5.08	0.37	0.20	0.00	0.69	0.20	0.00	G
U	2.01	1.82	0.00	2.17	3.23	0.85	1.80	2.63	0.85	2.70	3.03	4.24	A
	2.59	2.83	2.54	6.29	6.87	10.17	2.17	1.82	1.69	2.48	1.62	0.00	U
	0.21	0.00	0.00	2.27	3.03	5.08	0.85	0.61	0.00	1.48	0.40	1.69	C
	0.58	0.61	0.00	6.18	6.46	15.25	0.58	0.81	0.85	1.53	1.62	0.85	G
C	1.22	1.01	3.39	0.37	0.00	0.00	0.79	0.61	0.00	0.32	0.20	0.00	A
	1.90	2.22	0.00	2.11	2.22	5.08	0.32	0.40	0.00	0.37	0.81	0.00	U
	0.26	0.20	0.00	0.79	0.20	0.00	0.05	0.00	0.00	0.69	0.00	0.85	C
	0.37	0.20	0.00	0.63	0.40	0.00	0.63	0.00	0.85	0.74	0.40	0.85	G
G	1.48	2.42	2.54	0.32	0.00	0.00	0.69	0.40	0.00	0.79	0.81	0.00	A
	1.59	1.62	2.54	0.95	1.41	0.85	0.58	0.81	0.00	0.42	0.40	0.00	U
	0.16	0.40	0.00	0.26	0.20	0.00	0.74	0.20	0.00	0.90	0.20	0.00	C
	0.58	0.20	1.69	0.16	0.00	0.00	0.69	0.20	0.00	0.21	0.00	0.00	G

V^a^- The percentage of miRNA families belonging to Viridiplantae with a particular signature SSR. There are 1892 miRNA families to which Viridiplantae miRNAs belong. F^b^- The percentage of miRNA families belonging to Fabaceae with a particular signature SSR. There are 495 miRNA families to which *P. vulgaris* miRNAs belong. P^c^- The percentage of miRNA families belonging to *P. vulgaris* with a particular signature SSR. There are 118 miRNA families to which *P. vulgaris* miRNAs belong

### Prediction of new miRNAs in *P. vulgaris*

**Fig. 4:**
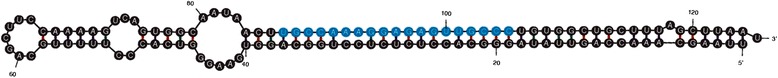
Secondary structure of a pre-miR (pvu-miR399a) showing the mature miRNA sequence highlighted in blue

The known Viridiplantae miRNAs from the miRBase 20 were used as query in the BLAST search with the EST and GSS sequences of *P. vulgaris* as subject*.* From the BLAST results satisfying the conditions mentioned in the ‘materials and methods’ section, a total of 141,724,357 sequences were extracted with all possible lengths. These sequences were used in BLASTX to identify and remove the protein coding sequences. After removal, the number of sequences reduced to 122,163,665. These sequences were examined for the seven criteria mentioned in the ‘materials and methods’ section, and only those fulfilling these criteria were retained as the predicted pre-miRs. In case of multiple sequences resulted from a single BLAST hit, the one which fulfils all the seven criteria with the maximum MFEI and the maximum R was retained. Finally, 310 sequences were obtained and were designated as putative pre-miRs in *P. vulgaris*. Extraction of the mature miRNAs from these 310 pre-miRs resulted in 208 new miRNAs, of which 201 are novel. These new miRNAs belong to 118 miRNA families in *P. vulgaris* (Additional file 2 Table S2). Fig. 4 shows a particular miRNA ‘pvu-miR399a’ that fulfils all the seven criteria used for the prediction.

**Fig. 5:**
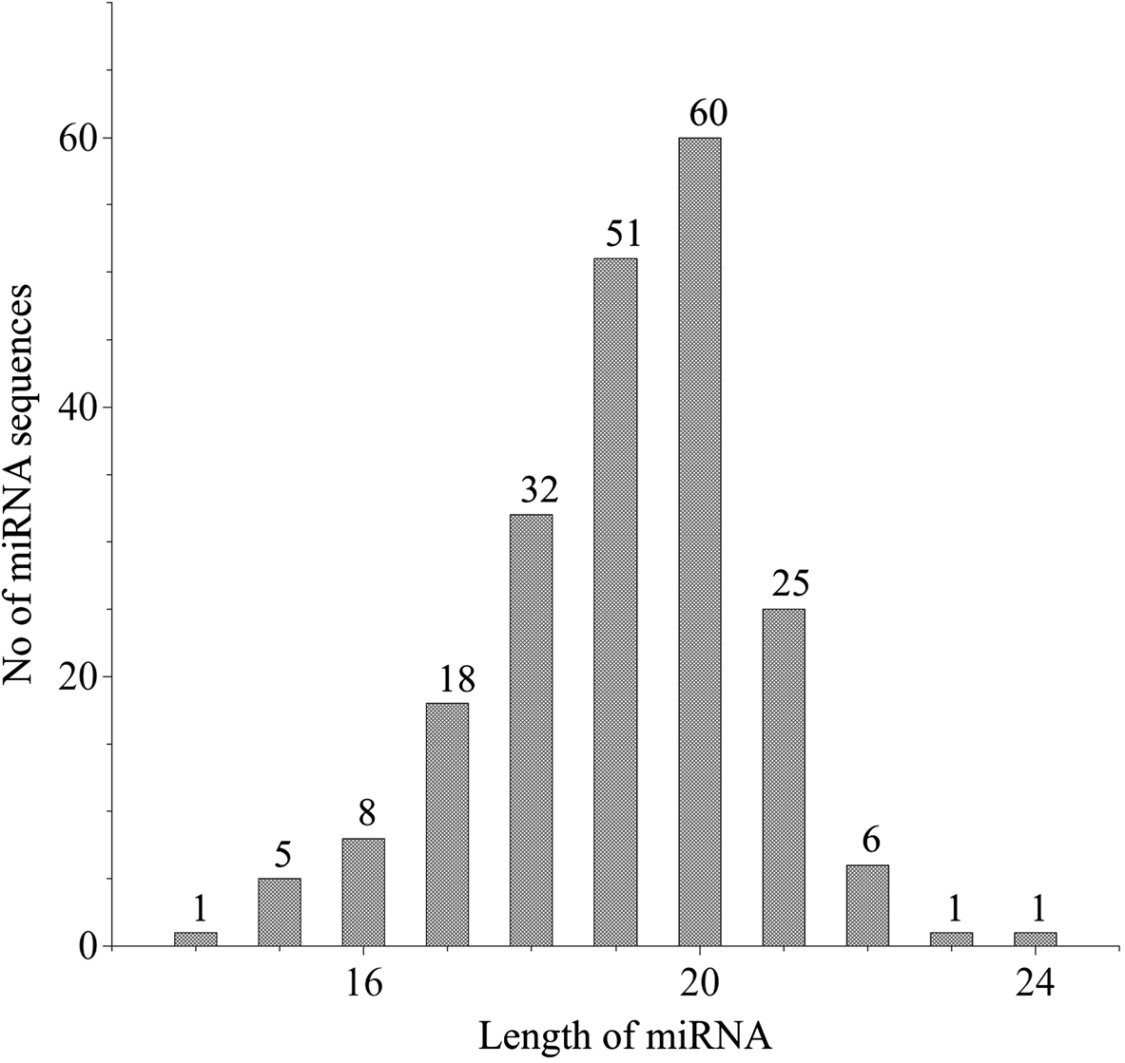
Frequency distribution of the length of mature miRNAs of *P. vulgaris*

The distribution of 208 newly predicted miRNAs in *P vulgaris* varies among the 118 miRNA families (Table 2). Four of the families namely MIR1533, MIR1527, MIR5021 and MIR848 are the most populated families with 15, 10, 10 and 7 members, respectively, while 85 families contain only one member. In the remaining 29 families, the number of miRNA varies from 2 to 5. This is in accordance with the diversity observed in other plant species [42]. The length distribution of newly predicted miRNAs (Fig. 5) shows that the length of mature miRNAs fall within the range of 15–24 nucleotides with an average length of 19 nucleotide (±1.6). However, miRNA pvu-miR848f is the only exception with the length of 14 nucleotides.

**Table 2:** Distribution of miRNAs within different miRNA families of P. vulgaris

miRNA families	Number of members/family
MIR1533	15
MIR1527	10
MIR5021	10
MIR848	7
MIR167, MIR171	5
MIR156, MIR159, MIR166, MIR169, MIR6034	4
MIR319, MIR3440, MIR5054, MIR529, MIR5721, MIR6470, MIR902	3
MIR1514, MIR2606, MIR2673, MIR3442, MIR396, MIR4345, MIR477, MIR5261, MIR5368, MIR5558, MIR5654, MIR5998, MIR6169, MIR829, MIR866	2
MIR1029, MIR1030, MIR1043, MIR1044, MIR1051, MIR1052, MIR1075, MIR1099, MIR1134, MIR1217, MIR1428, MIR1441, MIR1519, MIR165, MIR1846, MIR1860, MIR1888, MIR1916, MIR2082, MIR2088, MIR2095, MIR2105, MIR2109, MIR2610, MIR2873, MIR2934, MIR2938, MIR3444, MIR3630, MIR3633, MIR3711, MIR395, MIR3954, MIR3979, MIR398, MIR399, MIR408, MIR419, MIR4224, MIR4225, MIR4243, MIR4245, MIR4246, MIR4413, MIR482, MIR5014, MIR5041, MIR5057, MIR5083, MIR5140, MIR5169, MIR5176, MIR5177, MIR5179, MIR5213, MIR5248, MIR5255, MIR5264, MIR5281, MIR5298, MIR5555, MIR5562, MIR5662, MIR5674, MIR5675, MIR5741, MIR5773, MIR5778, MIR5820, MIR6027, MIR6114, MIR6167, MIR6171, MIR6196, MIR6214, MIR6479, MIR6484, MIR771, MIR773, MIR774, MIR831, MIR846, MIR861, MIR863, MIR919	1

### Experimental validation of the predicted miRNAs in *P. vulgaris*

Deep-sequencing of *P. vulgaris* small RNA library generated a total of 33,672,751 reads. The low quality reads as well the reads with lower than 14 nucleotide length were removed, resulting in 33,602,649 reads. The reads were made unique using fastx_collapser. The sequencing data obtained was BLAST searched with predicted miRNAs. The presence of 97 (Additional file 3 Table S3) of the predicted miRNAs in *P. vulgaris* is confirmed from the sequencing data.

**Fig. 6:**
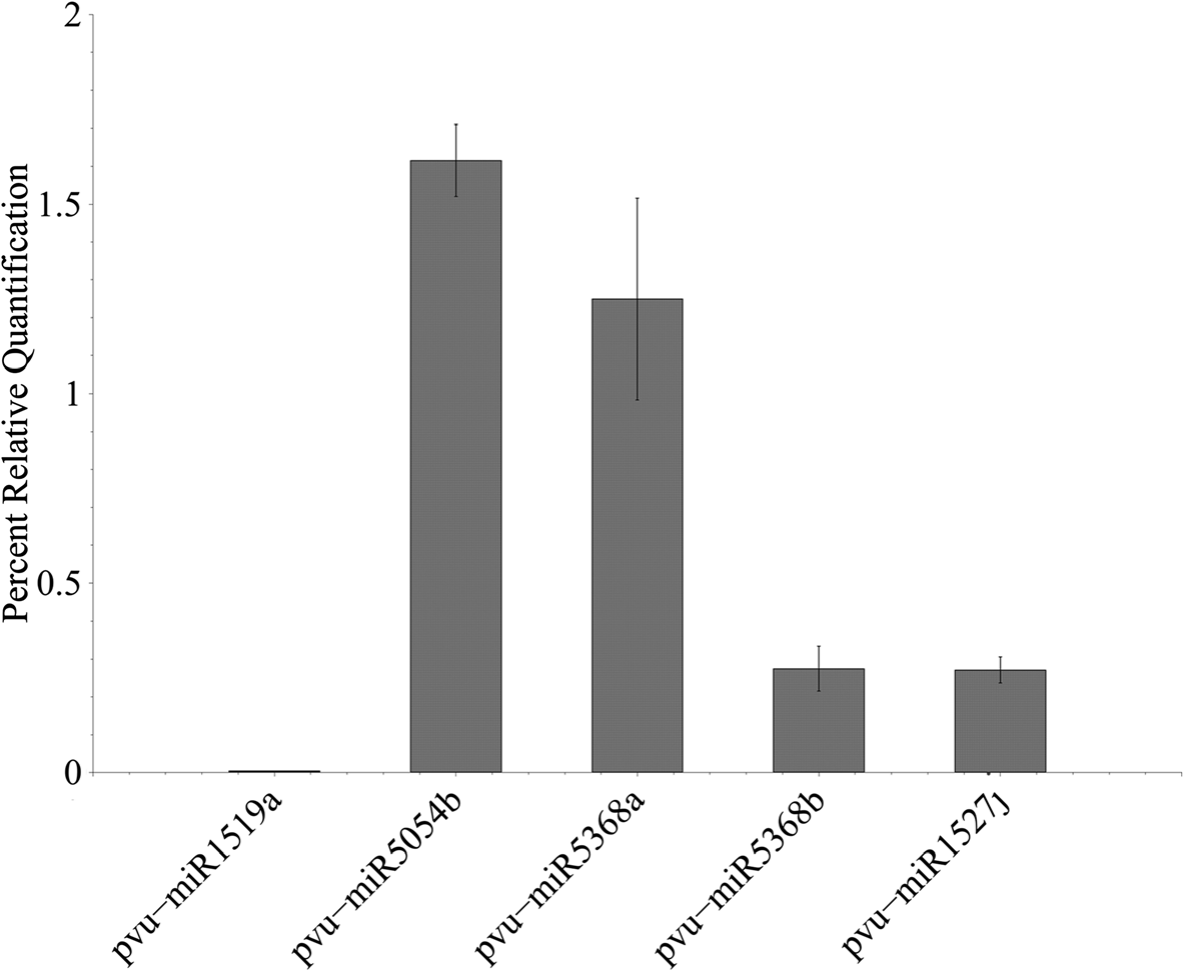
Expression profile of selected miRNAs from qRT-PCR analysis

**Fig. 7:**
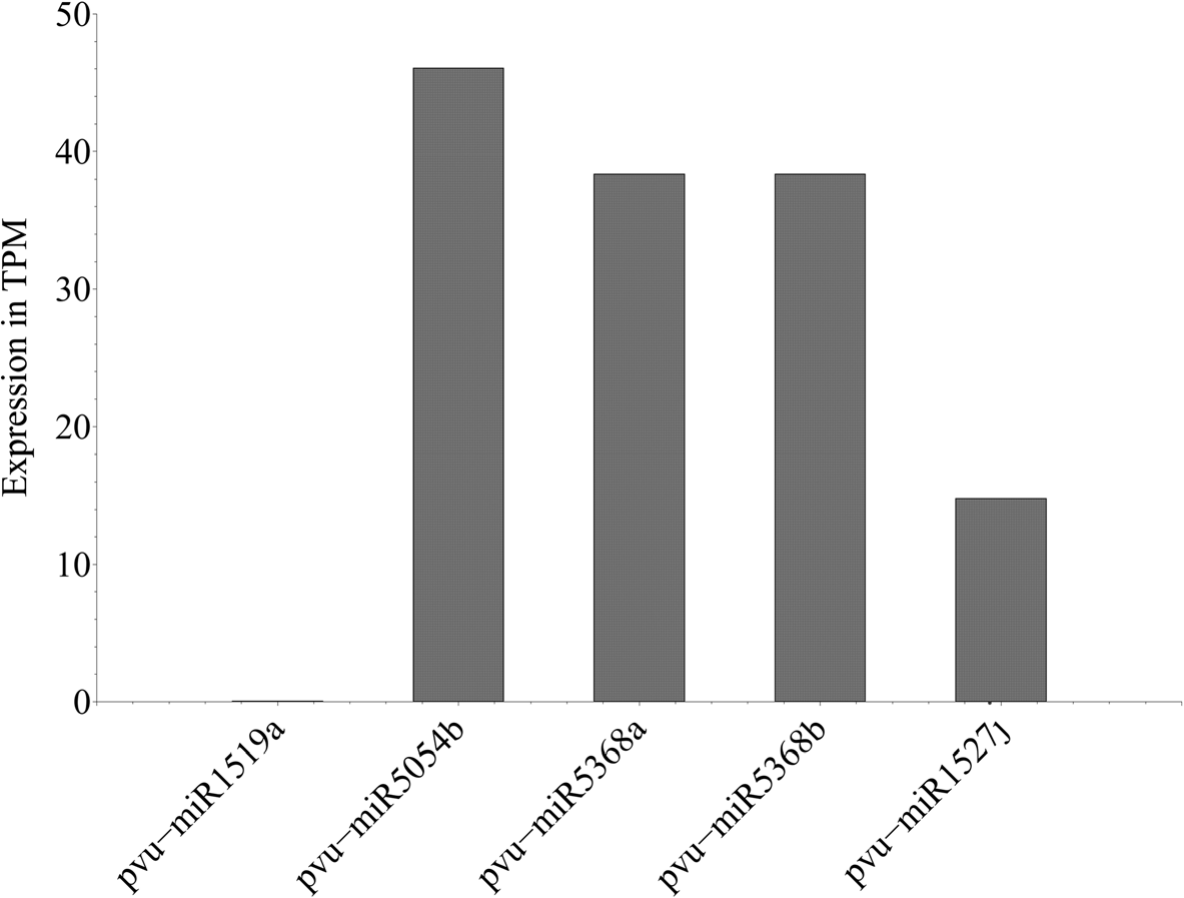
Expression profile in TPM of selected miRNAs from sequencing data

qRT-PCR was used to experimentally validate our computational method and to compare the results with the sequencing data. A total of 5 computationally predicted miRNAs were randomly chosen (Table 3) and qRT-PCR was done for these five miRNAs. C_T_ values were calculated using U6 snRNA as a normaliser gene. The relative quantity of each miRNA to U6 snRNA was expressed using the formula 2^-ΔCT^ [43], where ΔC_T_ = (C_T_ miRNA − C_T_U6 snRNA) (Fig. 6). The expression profiles obtained by qRT-PCR analysis mostly agreed with the expression values obtained from the sequencing data of these 5 miRNAs (Fig. 7). For pvu-miR1519a, in qRT-PCR, the C_T_ value obtained is quite high (34.4) indicating that it is a very low expressed miRNA and this result correlated with the sequencing data where the number of reads of this miRNA is only 2 (TPM 0.06). For pvu-miR5368b, the number of reads obtained from sequencing data is 1290 (TPM 38.4), the same value for pvu-miR5368a also, however, the relative expression obtained in qRT-PCR for pvu-miR5368b is lower than that of pvu-miR5368a. This may be due to the fact that pvu-miR5368b expression is relatively low in leaves compare to other tissues. Several studies already have established that miRNA expression can vary widely in different tissues or at different developmental stages [44, 45].

**Table 3:** Stem-loop reverse transcription primers for selected miRNAs

miRNA	miRNA Sequence	Primer sequences
pvu-miR1519a	AGUGUUGCAAGAUAGUCAUU	Reverse transcription primer: GTCGTATCCAGTGCAGGGTCCGAGGTATTCGCACTGGATACGACAATGAC
		Forward primer: CGGCGCAGTGTTGCAAGA
		Universal reverse primer: CCAGTGCAGGGTCCGAGGTA
pvu-miR5054b	UGGCGCCCACCGUGGGG	Reverse transcription primer: GTCGTATCCAGTGCAGGGTCCGAGGTATTCGCACTGGATACGACCCCCAC
		Forward primer: GGGGCCTGGCGCCCACCG
		Universal reverse primer: CCAGTGCAGGGTCCGAGGTA
pvu-miR5368a	GGACAGUCUCAGGUAGACA	Reverse transcription primer: GTCGTATCCAGTGCAGGGTCCGAGGTATTCGCACTGGATACGACTGTCTA
		Forward primer: CGGCGCCGGACAGTCTCAGG
		Universal reverse primer: CCAGTGCAGGGTCCGAGGTA
pvu-miR5368b	UGUCUACCUGAGACUGUCC	Reverse transcription primer: GTCGTATCCAGTGCAGGGTCCGAGGTATTCGCACTGGATACGACGGACAG
		Forward primer: CGGCGCCTGTCTACCTGAGA
		Universal reverse primer: CCAGTGCAGGGTCCGAGGTA
pvu-miR1527j	UAACUCAACCUUAUAAAAC	Reverse transcription primer: GTCGTATCCAGTGCAGGGTCCGAGGTATTCGCACTGGATACGACGTTTTA
		Forward primer: CGGCGCCTAACTCAACCTTA
		Universal reverse primer: CCAGTGCAGGGTCCGAGGTA

### Computational validation of the prediction method

The computational method developed in this study was used to predict the miRNAs of *A. thaliana* and the results were compared with known miRNAs of *A. thaliana* (miRBase 20). The miRNAs from Viridiplantae excluding those from *A. thaliana* and the genome of *A. thaliana* were used as the inputs for prediction pipeline. A total of 229 miRNAs (Additional file 4 Table S4) were predicted, of which 213 are already reported in miRBase 20. The same procedure was repeated for *G. max*. A total of 462 miRNAs (Additional file 5 Table S5) were predicted, of which 397 are already reported in miRBase 20. The performance of the prediction method is measured using parameters sensitivity, specificity, positive predictive value (PPV) and negative predictive value (NPV). Our computational prediction method has a high sensitivity of 0.97 as well as high specificity of 0.99 (Table 4).

**Table 4:** Statistical parameters to measure accuracy of prediction method

Parameter	*A. thaliana*	*G. max*
Sensitivity	0.97	0.97
Specificity	0.99	0.98
Positive predictive value	0.93	0.86
Negative predictive value	0.99	0.99

### Prediction of the miRNA targets in *P. vulgaris*

**Fig. 8:**
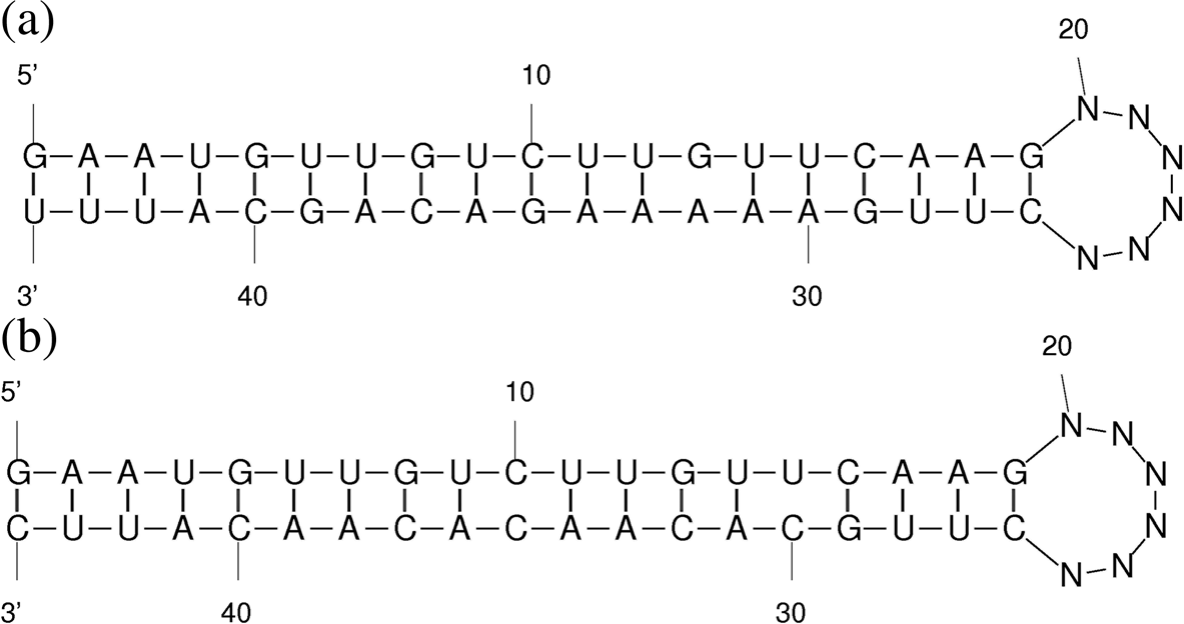
Hybridized structure of mature miRNA with its targets. The mature miRNA forms the 5′ end and the target is at the 3′ end separated by 6 nucleotides. The pvu-miR166d with its two targets: **(a)** EST 312062389 coding for UDP-N-acetylglucosamine pyrophosphorylase protein regulated by cleavage, **(b)** EST 312035414 coding for SNF1-related protein kinase regulatory subunit inhibited by translational regulation

The psRNATarget server was used to predict the miRNA targets. The default sequences of the target candidates in the server are of old version, hence the updated EST sequences of *P. vulgaris* from NCBI GenBank were used as target candidates. For 130 miRNAs that belong to 69 families, 1303 target sequences were predicted. In order to characterise the targets, BLASTX was used with the predicted target sequences as query and the entire protein sequences of Viridiplantae as subject. Using 80 % sequence identity cut-off, 318 targets for 95 miRNAs were characterised (Additional file 6 Table S6). For additional 339 targets for 80 miRNAs, the BLASTX predicted uncharacterised and hypothetical proteins. The hybridized structures of mature pvu-miR166d with its two targets, EST 312062389 coding for UDP-N-acetyl glucosamine pyrophosphorylase protein and EST 312035414 coding for SNF1-related protein kinase regulatory subunit are shown in Fig. 8.

## Discussion

In the last decade, numerous studies confirmed that plant miRNAs are directly involved in developmental processes such as seed germination, morphogenesis, floral organ identity, root development, vegetative and reproductive phase change, flowering initiation and seed production [46–51]. In addition to their important functions in organ development, plant miRNAs play a crucial role at the core of gene regulatory networks. They are involved in various biotic and abiotic stress responses, [52–54] signal transduction and protein degradation [55]. Plant miRNAs also play an important role in the biogenesis of small RNAs (siRNAs) and in the feedback regulation of siRNA pathways.

In the present study, using computational methods, we have identified 208 new miRNAs in *P. vulgaris* of which 201 are novel*.* Of these 208 predicted miRNAs, 97 were validated through small RNA sequencing. In general, computational prediction of miRNAs uses a highly constrained search space by setting fixed values to parameters like AU content, MFEI and the length of the pre-miRs [12, 13, 15, 16]. Constraining the parameters to a fixed cut-off value reduces the number of predicted miRNAs. It is already an established fact that the commonly used parameters namely the length of pre-miRs, AU content and MFEI are highly variable, ranging between 43–938, 17 %–92 % and 0.32–2.7, respectively. The distribution of ND, Npb and NQ (Fig. 2) in miRNAs is significantly different from other small RNAs, making them good candidates as prediction parameters. However, there is also an overlapping region in the distribution, which can result in false positives while predicting using single parameter. Thus using a combination of these parameters will make the prediction pipeline more robust. In the present study, instead of using the conventional computational procedure, where all the prediction parameters are set to a fixed value, we have used a 99 % probability range. Initial application of fixed cut-off values for various parameters resulted in only 26 new miRNAs in *P. vulgaris*. This low number of miRNAs prompted us to use the 99 % probability range with the anticipation of getting better prediction. After using the 99 % probability range for the first six parameters described in the ‘materials and methods’ section, 2538 pre-miRs in *P. vulgaris* were predicted, which is almost hundred times compared to the conventional method. However, it should be noted that the increased number includes both new predictions as well as false positives. False positives are eliminated by using the RNA folding parameters and conserved SSR signature.

The presence of SSRs in pre-miRNAs is already established [29–31], although their specific role in pre-miRs is still unknown. Most of the SSRs in pre-miRs have few steady characteristics, allowing their identification in pre-miRs feasible. Thus conserved SSR signatures are a potential parameter in predicting new miRNAs. In the present study, we have used the conserved SSR signatures as a prediction parameter. By using this parameter, the predicted number of 2538 *P. vulgaris* pre-miRs was reduced to 310. We have identified the SSR signatures for all the Viridiplantae miRNAs present in the miRBase 20 (Additional file 1 Table S1), and these signatures can be used for the identification of new miRNAs in any species of Viridiplantae.

Along with the SSR, we have also used NQ, ND and Npb in our prediction. After filtering the putative pre-miRs through these four parameters, the length, AU content and MFEI for the predicted pre-miRs of *P. vulgaris* vary from 55–105, 33–77 % and 0.42–1.2, respectively. These values are in agreement with known pre-miRs in Viridiplantae. These four independent parameters do not restrict the physical and thermodynamic features of pre-miRs to fixed values, and can be used for successful prediction of new miRNAs in plants.

The miRBase 20 contains 7385 mature miRNAs of Viridiplantae. Analysis of these 7385 miRNAs revealed that more than 70 % of them belong to the 13 well-studied plant species namely *Medicago truncatula, Oryza sativa, Glycine max, Brachypodium distachyon, Populus trichocarpa, Arabidopsis lyrata, Solanum tuberosum, Arabidopsis thaliana, Zea mays, Physcomitrella patens, Sorghum bicolor, Prunus persica* and *Malus domestica.* Further we find that, each of these 13 species have more than 200 mature miRNAs reported in the miRBase. In the present study, prediction of the 208 mature miRNAs in *P. vulgaris* is in accordance with this finding, thus justifying our modified computational prediction method.

In order to validate the computationally predicted miRNAs, small RNA library was prepared from the Anupam cultivar of *P. vulgaris*. The quality reads with more than 14 nucleotide length were BLAST searched with the predicted miRNAs. Out of the 208 predicted miRNAs, 97 are expressed in the sequenced sample. The read numbers for miRNAs showed high diversity, ranging from 1 to 37,259 for the expressed miRNAs. Among these miRNAs, the miR166 family had the most number of reads. For all the identified miRNAs, transcript per million (TPM) was also calculated. The dataset of known pre-miRs downloaded from the miRBase 20 contains miRNAs deposited from different cultivars of *P. vulgaris* at different developmental stages. However, the small RNA library created for sequencing is from a single cultivar of *P. vulgaris* at a particular stage of development, which makes it impossible for all the predicted 208 miRNAs to be present in the sequence library. The presence of nearly fifty percent of the predicted miRNAs in the sequencing data justifies our method followed in computational prediction of miRNAs.

Additionally, five randomly selected computationally predicted miRNAs were validated using qRT-PCR. Relative expressions obtained in the qRT-PCR mostly corroborated the sequencing data; only slight variation for pvu-miR5368b can be attributed to the fact that miRNA expression widely varies in different tissues and this particular miRNA may have relatively low expression in leaf tissues. The validation of the five randomly selected predicted miRNAs in both qRT-PCR and Illumina sequencing substantiate our computational method for the prediction of miRNAs.

All the newly predicted 208 miRNAs in *P. vulgaris* belong to 118 miRNA families. We find that of these 118 families, only 15 contain miRNAs distributed into 10 plant species. Although, these miRNA families have a wide species range, yet low number of miRNAs are present from the species of Fabaceae family (Table 5). There are 21 miRNA families containing a single miRNA from one of the species of Fabaceae, showing the under representation of miRNAs of Fabaceae in the miRBase. Fabaceae, one of the most important families in the Dicotyledonae [56], is rich in high quality protein, providing high nutritional food crops for agriculture all over the world. Our prediction of 208 new miRNAs in *P. vulgaris* as well as identification and characterisation of their targets will enrich the present knowledge of Fabaceae miRNAs, and will definitely help in deciphering the role of miRNAs in different regulatory mechanisms.

**Table 5:** Distribution of Fabaceae species in various miRNA families

miRNA Family	Number of Viridiplantae species	Number of Fabaceae species
156	48	3
159	35	2
166	42	3
167	37	4
169	36	3
171	41	4
319	34	6
395	30	3
396	42	5
398	30	2
399	30	4
408	32	5
482	23	5
529	10	1
1514	2	2
1519	1	1
1527	1	1
1533	1	1
2088	1	1
2109	2	2
2606	2	2
2610	1	1
2673	1	1

miRBase 20 contains 427 mature miRNAs of *A. thaliana* of which 220 homologs are present in other species of Viridiplantae. The rest of the known miRNAs (207) from *A. thaliana* have no known homolog in other plant species, making them difficult to predict. We have also predicted 213 miRNAs of the known homologs from a total prediction of 229 miRNAs in *A. thaliana*. Besides, we also predicted 462 miRNAs in *G. max* of which 397 exists in miRBase 20 (97 % of 408 reported miRNAs). This successful prediction not only validates our method, but also establishes that the method can be applied to predict the miRNAs in any other plant species.

The prediction method can be evaluated using various statistical parameters such as sensitivity, specificity, PPV and NPV. Sensitivity measures the proportion of miRNAs which are correctly identified by the prediction pipeline, whereas specificity measures the proportion of sequences which are correctly rejected. Our prediction method shows both high sensitivity and specificity when tested for known miRNAs of *A. thaliana* and *G. max* (Table 4). The parameters PPV and NPV measures the probability of predicted or rejected sequences to be true miRNAs or not, respectively. Higher values of PPV and sensitivity give us a high confidence for a positive prediction, while higher values of NPV and specificity give us high confidence for the rejection

Recently, numerous studies suggested that the genomic distribution of SSRs are nonrandom, and the SSRs located in gene or regulatory regions play important role in chromatin organization, regulation of gene activity, recombination, DNA replication, cell cycle, mismatch repair system [57, 58]. The transcriptome survey of several plant species showed the high abundance of di- and tri-nucleotide repeats compare to tetra-, penta- and hexa nucleotide repeats; (AT)_n_ repeat being the most frequently occurring microsatellites in plant genomes [59–63]. The microsatellites in the genomic sequences play vital role in the biogenesis of several small non-coding RNAs, of which most important are the miRNAs. Transcriptome analysis of several plants revealed that a significant percentage of the unigenes constitutes ‘SSR bearing pre-miRNA candidates’ [58], suggesting that SSRs are an important component of pre-miRs. SSRs in pre-miRs are derived from independent transcriptional units and often relate to function [32]. Variations of SSRs within pre-miRs are very critical for normal miRNA activity as expansion or contraction of SSRs in pre-miRs directly affects the corresponding miRNA products and may cause unpredicted changes [32]. These characteristics features foster exploit of SSR signature as a critical parameter in miRNA identification [32]. The number of miRNAs predicted in the traditional method is too low and we have introduced 99 % probability region for increasing the search space. However, this has increased the number of false predictions. As a result of this, the number of miRNAs predicted before the SSR filtering step for *A. thaliana* and *G. max* are 2082 and 3541, respectively. In spite of these high numbers of predictions, by using SSR the final numbers of predicted miRNAs were restricted to 229 and 462, respectively in these two species. The specificity of our prediction method improved from 0.62 to 0.99 in *A. thaliana* and 0.49 to 0.98 in *G. max*, by applying SSR filtration step. Thus SSR signatures act as an effective filtering parameter in limiting the number of false positives to acceptable limits.

The mature miRNA sequences and EST sequences of *P. vulgaris* were submitted to the psRNATarget server for the prediction of targets. The parameters were adjusted as described in ‘materials and methods’ section for better prediction. The hpsize [64] was changed according to the length of miRNA, as the server uses a value assuming the length of miRNA as 20 nucleotides. The miRNAs with length lesser than hpsize were ignored by the server pipeline. The length of the miRNAs predicted in the present study varies from 14–24 nucleotides. The sequence length of central mismatch was also changed according to the length of the miRNA. This parameter helps to predict the targets inhibited by translational regulation and has no effect on targets inhibited by cleavage of mRNA sequence [65]. Further, the maximum expectation value was set to 2.0 for stringent filtering of false positive targets predicted by the server.

In the present study, 1305 targets were predicted for 130 miRNAs. Of these 1305 targets, functional information was retrieved for 318 targets distributed in 46 miRNA families. In majority of the cases, the predicted targets in this study were in accordance with the already published reports in other plant species. Yu et al. [66] showed that miR156 family control plant development by regulating the trichome growth in *Arabidopsis*. It is already established that MYB transcription factors are the negative controllers of the trichome growth. The miR156 family targets the MYB transcription factor mRNAs, and by cleaving these transcription factors they positively control the trichome growth. We also found that the predicted pvu-miR156d target the MYB transcription factors. In the present study pvu-miR166d was predicted to target kinase mRNA, which is in agreement with the reported target kinase for miR166 family in soybean [67]. Calvino and Messing [68] established that miR169 family in Sorghum targets the carboxypeptidase mRNAs. Similarly, in the present study, pvu-miR169b was predicted to target the carboxyl-terminal-processing protease. Scarecrow-like transcription factor is already an established target for miR171 family in *Arabidopsis* [69] and *Oryza sativa* [70]. Similar results were obtained in our study where pvu*-*miR171a was predicted to bind Scarecrow-like transcription factor. ATP sulfyrylase responsible for sulphur (S) uptake and assimilation is the target for miR395 family in *Arabidopsis* [69], rice [70] and soybean [67]. Newly identified pvu*-*miR395a was also predicted to target the ATP sulfyrylase. In *Arabidopsis*, it was found that miR396 family targets the tubulin mRNAs [71]. Our prediction was in accordance with this finding, showing that pvu-miR396b targets gamma tubulin. Basic blue proteins (Plantacyanins) are validated targets for miR408 family in *Arabidopsis* and rice [70, 72]. Similar target was predicted for pvu-miR408a. The predicted target fatty acid desaturase of pvu-miR902c in our study is in agreement with the findings of Wan et al. [73] showing that the targets of miR902 are primarily involved in lipid metabolism.

## Conclusion

In this study, we have used computational method to identify new miRNAs in *P. vulgaris* and few of them were experimentally validated*.* We have used conserved SSR signatures to predict new miRNAs. We have identified 208 new miRNAs belonging to 118 different families of miRNAs in *P. vulgaris,* of which 201 are novel. We have also predicted 1305 targets for 130 of these miRNAs. We successfully predicted known miRNAs *in A. thaliana* and *G. max* using our method. Presently, numerous miRNAs from various plant species have been identified and characterized by the aid of next-generation sequencing. However, there is still inadequate information of miRNAs in many plant species. Identification of new miRNAs in all plant species and deciphering their functions is the present day challenge in biological discoveries. Wet-lab experiments have their own limitations and the alternate approach is *in silico* methods for miRNA studies. *In silico* methods can rapidly identify new miRNAs and their targets in any species. The computational approach that we have developed can be successfully applied to identify new miRNAs and their targets in any plant species, and is expected to generate an optimal framework for deciphering the biogenesis, functions, and mechanisms of plant miRNAs that are not yet discovered.

## Methods

### Data collection and preparation

The Viridiplantae pre-miRs were downloaded from the miRBase 20 (Release 20: June 2013) [74] and used as the standard dataset of known pre-miRs. The small RNAs belonging to different families were downloaded from Rfam 11 [75] for comparative analysis of various parameters. The miRBase 20 contains 24,521 pre-miRs, of which 6088 belong to Viridiplantae. Besides, we have also downloaded 125,490 Expressed Sequence Tags (ESTs) and 92,534 Genomic Survey Sequences (GSSs) of *P. vulgaris* (txid3885) from the GenBank [76]. Removal of the redundant sequences resulted in 2560 Viridiplantae pre-miRs, and 122,157 EST and GSS sequences of *P. vulgaris*. Protein sequences of *P. vulgaris* were downloaded from the protein database (http://www.ncbi.nlm.nih.gov/protein). Genomes of *A. thaliana* [77] and G. max [78] were downloaded from Phytozome [79].

### Analysis of known precursor sequences

All the downloaded 6088 Viridiplantae pre-miRs were used to calculate the length of pre-miRs sequences (L), AU content and MFEI. The structures with the minimum folding energy was generated using RNAfold [80]. The MFEI value was calculated using the Adjusted MFE (AMFE), which represents the MFE for 100 nucleotides.

$$ AMFE=\frac{-MFE}{\mathrm{L}}\times 100 $$

$$ MFEI=\frac{AMFE}{\left(G+C\right)\%} $$

The genRNAstats program [81] was used to calculate the NQ, ND and Npb for all known pre-miRs of Viridiplantae. Npb is the measure of total number of base pairs present in the RNA secondary structure per length of the sequence, and the value can range from 0.0 (no base-pairs) to 0.5 (L/2 base-pairs) [82]. The base-pairing probability distribution (BPPD) per base in a sequence were measured using NQ [83], while the base-pair distance for all the pair of structures were measured using ND [84]. Both the parameters ND and NQ were calculated from the base-pair probability p_ij_ between bases i and j.

$$ NQ=-\frac{1}{L}{\displaystyle \sum_{i<j}{p}_{ij}}.{ \log}_2\left({p}_{ij}\right) $$

$$ ND=\frac{1}{L}{\displaystyle \sum_{i<j}{p}_{ij}\left(1-{p}_{ij}\right)} $$

The miRBase 20 classified Viridiplantae miRNAs into 1892 families. We have checked the presence of conserved SSR signatures within the pre-miRs of all these families. The conserved SSR signatures were counted in all sequences for window size ranging from three to six. Due to the variable length of the pre-miRs, the SSR signatures were normalized per 100 nucleotides by the following equation.

$$ R=\frac{\mathrm{Number}\ \mathrm{of}\ \mathrm{S}\mathrm{S}\mathrm{R}\ \mathrm{signatures}}{\mathrm{L}}\times 100 $$

### Prediction of new miRNAs in *P. vulgaris*

**Fig. 9:**
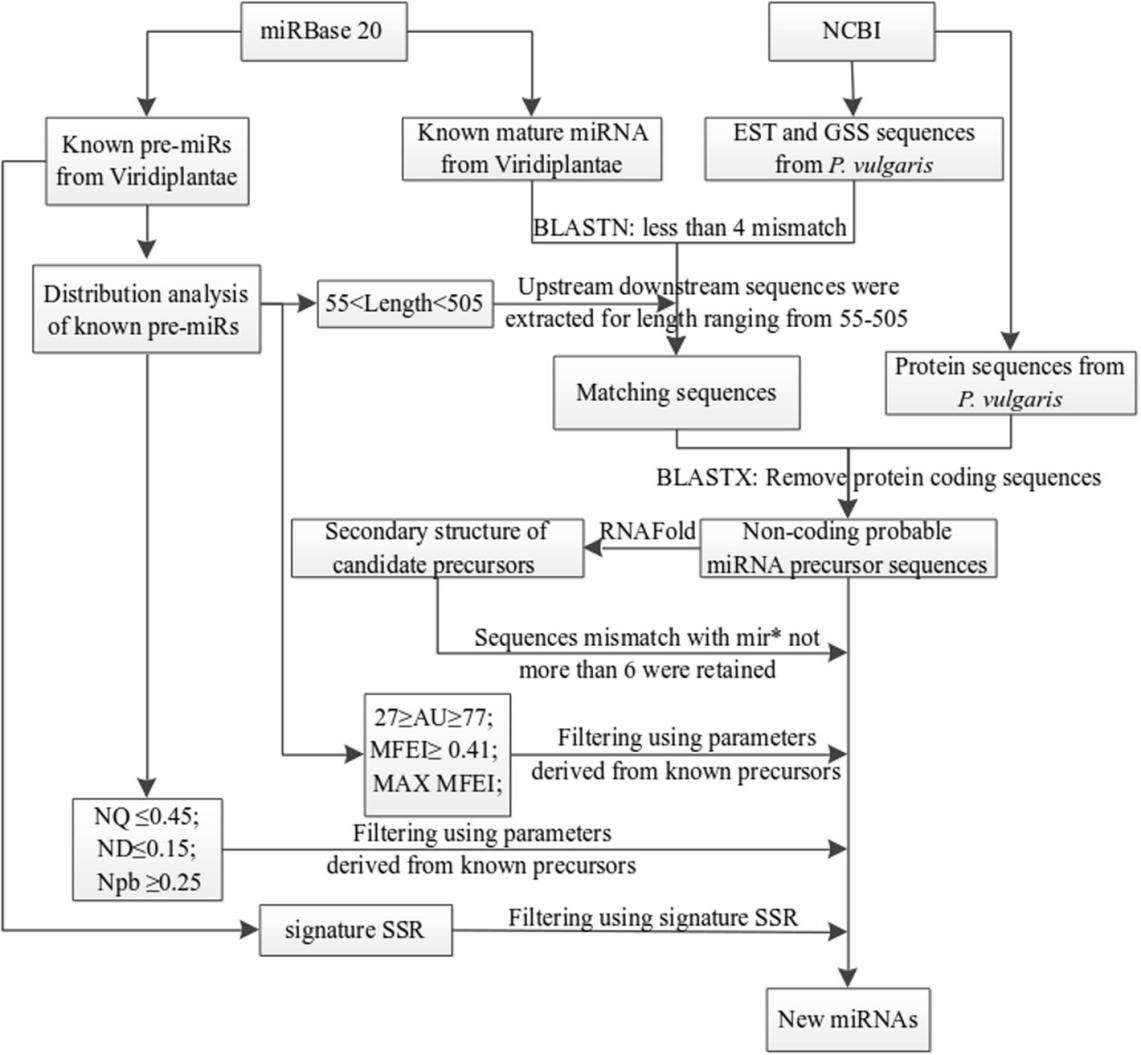
Schematic representation of the computational method followed in the prediction of new miRNAs in *P. vulgaris*

BLAST search [85] was performed using the non-redundant dataset of Viridiplantae pre-miRs as query and non-redundant dataset of EST and GSS sequences of *P. vulgaris* as subject, with an e-value cut-off of 1000, word size 7 and mismatch less than 4 [86]. The upstream and (or) downstream sequences with all possible lengths ranging from 55–505 were extracted from EST and GSS sequences that aligned with the miRNAs. In order to remove the protein coding sequences, an ungapped BLASTX with the sequence identity cut-off ≥ 80 % was performed with all the extracted sequences as query and the protein sequences of *P. vulgaris* as subject. After removal of the protein coding sequences, remaining sequences satisfying the following criteria were designated as the predicted precursor sequences: (i) formation of an appropriate stem-loop hairpin secondary structure with minimum free energy of folding and MFEI ≥ 0.41, (ii) a mature miRNA sequence located in one arm of the hairpin structure, (iii) miRNA sequence having less than 6 mismatches with the opposite miRNA* sequence on the other arm of the hairpin structure, (iv) without any loop or break in miRNA* sequence, (v) AU content of the sequences within the range 22-77 %, (vi) values for the parameters NQ, ND and Npb should be ≤ 0.45, ≤ 0.15 and ≥ 0.25, respectively and (vii) presence of SSR signature in the corresponding miRNA family with R ≥ 2.5. In case of multiple sequences resulted from a single BLAST hit, the particular sequence that fulfils all the above seven criteria along with the maximum values of MFEI and R was chosen. The above mentioned steps are presented in a schematic diagram in Fig. 9. Mature miRNAs were extracted from the predicted pre-miRs satisfying the above criteria.

### Experimental validation of predicted miRNAs of *P. vulgaris*

#### Plant material

Healthy seeds of Anupam cultivar of *P. vulgaris* were surface sterilized with 0.5 % Sodium hypochlorite and germinated in dark at 28 °C for 2 days. Germinated seeds were allowed to grow in soilrite in BOD at 24 °C for 10 days. Seedlings of 10 days old were sent in RNAlater (Sigma-Aldrich) to Genotypic-Bangalore, India, for further library preparation and sequencing.

#### Sequencing

Library preparation was performed at Genotypic Technology’s Genomics facility following certified protocols from NEXT Flex. Small RNA libraries for sequencing were constructed according to the NEXTflex™ small RNA library protocol outlined in NEXTflex™ Small RNA Sequencing Kit - 5132–02. 1.6 μg of total RNA was used as the starting material. Briefly, 3′ adaptors were ligated to the specific 3’OH group of small RNA followed by 5′ adaptor ligation. The ligated products were reverse transcribed by priming with reverse transcriptase primers. The cDNA was enriched by PCR (12 cycles) and size selection was done using 8 % polyacrylamide gel. The library was size selected in the range of 140 – 160 bp, followed by overnight gel elution and salt precipitation using glycogen, 3 M sodium acetate and absolute ethanol. The precipitate was re-suspended in resuspension buffer. The prepared library was quantified using Qubit fluorometer, and validated for quality by running an aliquot on high sensitivity Bioanalyzer Chip (Agilent). The Bioanalyzer profiles showing fragments between ~130 to ~160 bp with insert size being ~10 to ~40 bp were sent for sequencing.

#### Sequencing data analysis

The quality reads from the sequencing data were extracted and the adapter sequences were removed using cutadapt [87]. The sequences smaller than 14 nucleotides were removed. The reads were made unique for easy analysis by using fastx_collapser (http://hannonlab.cshl.edu/fastx_toolkit/index.html). The predicted miRNAs of *P. vulgaris* were BLAST searched against the sequencing data to validate the predictions.

#### Small RNA isolation

Small RNA was isolated from leaves of 10 days old seedlings of Anupam cultivar using mirPremier microRNA isolation kit (Sigma-Aldrich) according to the manufacturer’s instruction. The quality and quantity of the isolated small RNA was measured using a microvolume spectrophotometer (JENWAY 7310) and stored at −20 °C.

#### cDNA synthesis and primer design

Small RNA was reverse transcribed to cDNA using stem-loop reverse transcription primers for miRNAs as listed in Table 3 following a pulsed RT reaction [88]. Stem-loop primers, forward and reverse primers were designed according to Kramer [89]. A three step pulsed RT reaction was performed; an initial denaturation step at 80 °C for 5 min containing 20 ng of small RNA and 1 μM of each gene specific primers, followed by primer annealing incubation step at 60 °C for 5 min, followed by final addition of reaction mixture containing 500 μM dNTP, 1X buffer, RNase inhibitor and enhanced avian reverse transcriptase (Sigma-Aldrich).

#### Quantitative Real Time Polymerase Chain Reaction (qRT-PCR)

qRT-PCR reactions were carried out for the five selected miRNAs in a Bio-Rad CFX96 Real-Time PCR system using Bio-Rad iQ SYBR green supermix. Gradient PCR (50 °C-60 °C) was performed to select the ideal annealing temperature of 58 °C for the amplification of U6 snRNA (endogenous control) and selected miRNAs. The reaction mixture containing 1X SYBR green supermix, 350 nM of each gene specific forward and reverse primers and cDNA (100 ng) was then incubated at 95 °C for 2 min., followed by 40 cycles of 95 °C for 10 s and 58 °C for 20 s. Melting curve analysis was carried out to verify the specificity of each amplicons. Each amplification reaction was done in triplicate and the specificity of amplicons was confirmed by the presence of a single peak. Standard curve was prepared for U6 snRNA using a twofold dilution.

### Computational validation of the prediction method

The computational method developed in this study to predict miRNAs was validated by predicting known miRNAs in *A. thaliana* and *G. max*. The known miRNAs (excluding those from *A. thaliana*) from Viridiplantae and the genome of *A. thaliana* were used as inputs for validation. Similar procedure was followed for *G. max.* The sensitivity, specificity, PPV and NPV for the prediction was calculated from true positives (TP), true negatives (TN), false positives (FP) and false negatives (FN) using following equations.

$$ \begin{array}{l} Sensitivity=\frac{TP}{TP+FN}\\ {} Specificity=\frac{TN}{TN+FP}\end{array} $$

$$ \begin{array}{l}PPV=\frac{TP}{TP+FP}\\ {}NPV=\frac{TN}{TN+FN}\end{array} $$

### Target prediction of new miRNAs

The targets for mature miRNAs were predicted using psRNATarget server [90] by submitting the mature miRNAs as query and the EST sequences of *P. vulgaris* as subject. To reduce the number of false predictions, the maximum expectation threshold was set to a stringent value of 2.0. The nucleotides for complementarity scoring, hpsize [64] were selected as equal to the length of the mature miRNAs. The maximum energy of unpairing (UPE) the target site was set as 25 kcal [64]. The flanking length around the target site was selected as 17 nucleotides upstream and 13 nucleotides downstream [91]. Due to the variable length of the mature miRNAs, the sequence range of the central mismatch was adjusted (Table 6). To predict the function of the target sequences, BLASTX was performed against the protein database of the Viridiplantae using 80 % sequence identity cut-off.

**Table 6:** Adjusted parameters for miRNA target prediction using psRNATarget server

Length of miRNA	Length for complementarity scoring	Range of central mismatch leading to translational inhibition
14	14	6-8
15	15	7-8
16	16	7-9
17	17	8-9
18	18	8-10
19	19	9-10
20	20	9-11
21	21	10-11
22	22	10-12
23	23	11-12
24	24	11-13

### Availability of supporting data

The data set supporting the results of this article is available in the NCBI’s Gene Expression Omnibus (GEO) database [92] under accession number GSE68305 (http://www.ncbi.nlm.nih.gov/geo/query/acc.cgi?acc=GSE68305).

## Additional files

Additional file 1: Table S1. SSR signatures for various miRNA families of Viridiplantae.

Additional file 2: Table S2. Predicted miRNAs of *P. vulgaris*.

Additional file 3: Table S3. *P. vulgaris* miRNAs obtained from both computational prediction and small RNA sequencing.

Additional file 4: Table S4. Predicted miRNAs of *A. thaliana.*

Additional file 5: Table S5. Predicted miRNAs of *G. max.*

Additional file 6: Table S6. Predicted targets of *P. vulgaris* miRNAs.

## Abbreviations

miRNAs: microRNAs
SSR: Simple sequence repeat
qRT-PCR: Quantitative Real-time polymerase chain reaction
MFEI: Minimal Folding free Energy Index
pre-miRs: miRNA precursor sequences
RISC: RNA-induced silencing complex
NGS: Next-Generation Sequencing
EST: Expressed Sequence Tags
NQ: Normalised Shannon entropy
ND: Normalized base-pair distance
Npb: Normalized base-pairing propensity
PPV: Positive predictive value
NPV: Negative predictive value
TP: True positives
TN: True negatives
FP: False positives
FN: False negatives
