# Supplementary material for: Computational prediction of miRNAs and their targets in Phaseolus vulgaris using simple sequence repeat signatures
Source: BMC Plant Biol. 2015 Jun 12;15:140. doi: 10.1186/s12870-015-0516-3 (PMC4464996; doi:10.1186/s12870-015-0516-3)
Supplement: Supplementary file 1 — SSR signatures for various miRNA families of Viridiplantae. [file 12870_2015_516_MOESM1_ESM.docx]

# **Table S1: SSR signatures for various miRNA families of Viridiplantae.**

| miRNA family | Number of pre-miRs | Signature SSR | R^a^ | miRNA family | Number of pre-miRs | Signature SSR | R^a^ | miRNA family | Number of pre-miRs | Signature SSR | R^a^ |
| --- | --- | --- | --- | --- | --- | --- | --- | --- | --- | --- | --- |
| 156 | 288 | AGA | 3.20 | 1162 | 1 | CGG | 4.90 | 5537 | 1 | AAU | 5.38 |
| 157 | 12 | AGA | 4.19 | 1163 | 1 | GCG | 4.00 | 5538 | 1 | AGG | 5.69 |
| 158 | 4 | UGU | 3.83 | 1164 | 1 | UGC | 4.27 | 5540 | 1 | CGC | 4.08 |
| 159 | 86 | UUG | 3.24 | 1165 | 1 | CGC | 2.96 | 5541 | 1 | AAA | 4.64 |
| 160 | 136 | AUG | 4.01 | 1166 | 1 | GCC | 7.26 | 5542 | 1 | UAC | 5.92 |
| 161 | 3 | UUU | 3.95 | 1167 | 1 | GCA | 6.74 | 5543 | 1 | AAU | 4.90 |
| 162 | 40 | AUC | 3.90 | 1168 | 1 | CCU | 4.66 | 5544 | 1 | UUG | 4.88 |
| 163 | 2 | GAA | 3.16 | 1169 | 1 | AGC | 4.17 | 5555 | 1 | UAU | 5.26 |
| 164 | 112 | GCA | 3.85 | 1170 | 1 | UCA | 4.82 | 5556 | 1 | UCC | 4.96 |
| 165 | 4 | AUG | 4.89 | 1171 | 1 | GUG | 8.02 | 5557 | 1 | UUG | 4.76 |
| 166 | 245 | UUC | 3.54 | 1172 | 1 | UGC | 3.68 | 5558 | 1 | AUU | 6.98 |
| 167 | 157 | UGA | 3.42 | 1173 | 1 | UGC | 4.51 | 5560 | 1 | UCA | 5.41 |
| 168 | 46 | CGG | 3.74 | 1211 | 1 | GCA | 5.36 | 5561 | 1 | AAU | 6.74 |
| 169 | 342 | UUG | 3.67 | 1212 | 1 | UGC | 5.62 | 5562 | 1 | CAA | 4.91 |
| 170 | 2 | CUC | 6.83 | 1214 | 1 | UUU | 4.30 | 5563 | 1 | AAC | 4.95 |
| 171 | 226 | UUG | 3.62 | 1215 | 1 | GCU | 3.94 | 5566 | 1 | CUC | 3.77 |
| 172 | 154 | AUG | 3.85 | 1216 | 1 | UGU | 5.70 | 5567 | 1 | AUU | 4.67 |
| 173 | 3 | AAG | 4.97 | 1217 | 1 | UUG | 5.41 | 5569 | 1 | AUA | 6.07 |
| 319 | 98 | CUU | 2.94 | 1218 | 1 | ACA | 4.35 | 5570 | 1 | UGU | 5.66 |
| 390 | 65 | AUC | 3.58 | 1221 | 1 | UGG | 5.65 | 5573 | 1 | GAA | 4.27 |
| 391 | 3 | UCU | 2.95 | 1309 | 1 | UGA | 3.75 | 5574 | 1 | UUA | 5.33 |
| 393 | 62 | AUC | 4.87 | 1315 | 1 | AGG | 5.73 | 5575 | 1 | AAU | 6.06 |
| 394 | 46 | UCU | 3.77 | 1316 | 1 | AAU | 5.50 | 5576 | 1 | AAA | 7.86 |
| 395 | 220 | ACU | 3.21 | 1320 | 1 | AUU | 7.29 | 5577 | 1 | AAU | 3.45 |
| 396 | 124 | CUU | 4.39 | 1423 | 1 | CAC | 3.68 | 5578 | 1 | CAU | 10.67 |
| 397 | 38 | UGA | 3.64 | 1424 | 1 | UCC | 4.00 | 5595 | 1 | AUC | 4.96 |
| 398 | 58 | UCA | 2.71 | 1425 | 1 | AAU | 4.90 | 5628 | 1 | AUA | 6.16 |
| 399 | 200 | UGC | 3.77 | 1426 | 1 | AAA | 6.15 | 5629 | 1 | UAA | 7.16 |
| 400 | 2 | AUU | 4.22 | 1427 | 1 | GCG | 7.00 | 5631 | 1 | AUA | 8.94 |
| 402 | 2 | CUU | 4.04 | 1429 | 1 | UGU | 5.83 | 5632 | 1 | AAA | 4.38 |
| 403 | 33 | AUC | 3.73 | 1430 | 1 | UGG | 6.34 | 5633 | 1 | UAU | 4.78 |
| 405 | 3 | UAA | 5.52 | 1431 | 1 | UUA | 3.75 | 5634 | 1 | UUU | 3.98 |
| 408 | 47 | AGA | 3.28 | 1438 | 1 | AAU | 7.49 | 5636 | 1 | UUA | 5.13 |
| 413 | 2 | AGU | 3.43 | 1439 | 1 | AAU | 5.23 | 5637 | 1 | CUC | 5.13 |
| 414 | 3 | CAU | 8.78 | 1441 | 1 | UUU | 5.00 | 5639 | 1 | GGU | 3.66 |
| 415 | 2 | AGA | 5.16 | 1442 | 1 | AUC | 3.78 | 5640 | 1 | AUU | 3.47 |
| 416 | 2 | GUU | 4.79 | 1445 | 1 | AAU | 5.38 | 5641 | 1 | UUC | 5.09 |
| 417 | 2 | AAU | 5.92 | 1447 | 1 | AUU | 5.17 | 5644 | 1 | AUC | 4.83 |
| 418 | 2 | AAU | 5.25 | 1448 | 1 | UAA | 3.53 | 5646 | 1 | AGG | 5.71 |
| 419 | 3 | AUG | 5.46 | 1449 | 1 | UAA | 8.70 | 5647 | 1 | UUU | 3.81 |
| 426 | 2 | AAA | 5.41 | 1450 | 1 | UUG | 4.64 | 5648 | 1 | UUU | 4.20 |
| 437 | 29 | UUU | 4.05 | 1517 | 1 | UUU | 5.04 | 5650 | 1 | UUU | 6.02 |
| 439 | 9 | UGU | 7.11 | 1518 | 1 | ACA | 5.07 | 5651 | 1 | AUA | 4.94 |
| 444 | 16 | UGC | 5.66 | 1519 | 1 | UUU | 3.75 | 5652 | 1 | AUC | 4.02 |
| 447 | 3 | UUG | 4.17 | 1522 | 1 | UAA | 9.57 | 5653 | 1 | UUG | 6.98 |
| 472 | 5 | UUU | 3.42 | 1524 | 1 | UCC | 4.76 | 5655 | 1 | AGA | 4.65 |
| 473 | 3 | GCU | 4.05 | 1525 | 1 | UAA | 5.47 | 5656 | 1 | UUU | 4.58 |
| 474 | 3 | UGG | 5.06 | 1526 | 1 | GAU | 6.32 | 5657 | 1 | AAC | 4.71 |
| 475 | 4 | CAU | 4.52 | 1527 | 1 | AAC | 2.90 | 5658 | 1 | UGA | 7.45 |
| 476 | 3 | AUU | 5.21 | 1528 | 1 | AUU | 6.79 | 5659 | 1 | UUC | 6.78 |
| 477 | 45 | CUU | 3.14 | 1529 | 1 | UUA | 9.45 | 5660 | 1 | ACC | 3.08 |
| 478 | 18 | UUA | 5.68 | 1530 | 1 | UAU | 10.74 | 5661 | 1 | UCU | 7.41 |
| 479 | 6 | UCA | 4.88 | 1531 | 1 | CUU | 6.09 | 5662 | 1 | AAU | 4.53 |
| 481 | 4 | UAA | 3.52 | 1532 | 1 | AGC | 5.26 | 5663 | 1 | AUA | 4.46 |
| 482 | 63 | UUC | 4.29 | 1533 | 1 | UAU | 7.14 | 5664 | 1 | UAU | 5.14 |
| 528 | 6 | CCU | 4.79 | 1534 | 1 | AUU | 6.17 | 5665 | 1 | AAU | 5.85 |
| 529 | 16 | UCU | 3.77 | 1536 | 1 | AUG | 4.56 | 5666 | 1 | AAA | 4.49 |
| 530 | 23 | UGC | 5.18 | 1847 | 1 | ACA | 4.55 | 5667 | 1 | UUC | 5.68 |
| 531 | 3 | GCC | 7.69 | 1848 | 1 | CGC | 7.94 | 5668 | 1 | AAG | 4.42 |
| 533 | 5 | CUC | 4.34 | 1849 | 1 | CUA | 7.78 | 5669 | 1 | UGG | 7.04 |
| 534 | 2 | UUG | 4.97 | 1850 | 1 | UCA | 4.51 | 5670 | 1 | CAU | 5.15 |
| 535 | 22 | UGU | 3.78 | 1851 | 1 | GGC | 6.37 | 5671 | 1 | UGA | 5.88 |
| 536 | 7 | UUG | 3.27 | 1852 | 1 | AUU | 5.19 | 5672 | 1 | AAU | 5.26 |
| 537 | 4 | UGA | 4.86 | 1853 | 1 | AUU | 4.49 | 5673 | 1 | GAA | 6.54 |
| 538 | 3 | AUG | 5.30 | 1854 | 1 | AUU | 7.41 | 5675 | 1 | AAU | 4.78 |
| 771 | 2 | AUG | 5.51 | 1855 | 1 | CUC | 7.27 | 5676 | 1 | CCU | 4.11 |
| 773 | 3 | UUG | 4.36 | 1856 | 1 | GCA | 4.70 | 5677 | 1 | AAG | 4.02 |
| 774 | 4 | AUU | 6.49 | 1857 | 1 | AAA | 5.38 | 5678 | 1 | AAU | 4.13 |
| 781 | 3 | UAA | 5.40 | 1859 | 1 | UGA | 6.19 | 5679 | 1 | UGA | 6.33 |
| 810 | 2 | AUA | 4.58 | 1860 | 1 | UUU | 4.73 | 5711 | 1 | AUA | 3.70 |
| 812 | 22 | UUA | 4.97 | 1864 | 1 | GGG | 4.69 | 5712 | 1 | AUU | 6.34 |
| 814 | 3 | ACA | 3.59 | 1865 | 1 | ACU | 4.17 | 5713 | 1 | GUU | 3.36 |
| 815 | 3 | GAG | 4.84 | 1866 | 1 | AUU | 7.06 | 5714 | 1 | CAA | 4.24 |
| 818 | 6 | UAU | 4.68 | 1868 | 1 | AAA | 4.14 | 5715 | 1 | AAG | 6.45 |
| 820 | 3 | GGA | 4.81 | 1869 | 1 | AGG | 5.07 | 5716 | 1 | UUC | 4.37 |
| 821 | 8 | AAA | 4.22 | 1870 | 1 | CAU | 4.31 | 5717 | 1 | UCU | 5.36 |
| 822 | 2 | CAU | 4.56 | 1871 | 1 | AUC | 5.19 | 5718 | 1 | AAC | 4.27 |
| 823 | 2 | AAU | 3.79 | 1872 | 1 | UGU | 6.41 | 5719 | 1 | AUC | 4.90 |
| 824 | 5 | AUU | 2.99 | 1873 | 1 | AUG | 4.06 | 5720 | 1 | AUA | 4.83 |
| 825 | 2 | AAG | 4.45 | 1874 | 1 | AUA | 3.97 | 5721 | 1 | UUU | 5.06 |
| 827 | 17 | CAU | 5.91 | 1875 | 1 | UUG | 4.76 | 5722 | 1 | AUU | 6.02 |
| 828 | 16 | AUG | 4.01 | 1876 | 1 | UGG | 7.01 | 5723 | 1 | AUU | 4.29 |
| 829 | 2 | UGA | 4.94 | 1877 | 1 | AAA | 4.98 | 5724 | 1 | AAC | 2.90 |
| 831 | 2 | AAG | 3.64 | 1879 | 1 | AUG | 4.00 | 5725 | 1 | UUA | 5.37 |
| 833 | 3 | CAA | 4.47 | 1880 | 1 | AUU | 3.88 | 5726 | 1 | AAG | 3.51 |
| 834 | 2 | AGC | 4.43 | 1881 | 1 | AAG | 5.62 | 5740 | 1 | UAA | 4.00 |
| 835 | 2 | UCU | 3.46 | 1916 | 1 | UGA | 3.74 | 5742 | 1 | GAU | 5.73 |
| 837 | 2 | UUU | 6.35 | 1917 | 1 | UAA | 6.11 | 5744 | 1 | UAA | 4.45 |
| 838 | 2 | UCU | 4.81 | 1918 | 1 | AAU | 6.00 | 5746 | 1 | GUU | 7.59 |
| 839 | 2 | UGA | 3.40 | 2055 | 1 | AAG | 5.00 | 5747 | 1 | ACA | 6.25 |
| 840 | 2 | UUU | 3.61 | 2077 | 1 | UGA | 4.03 | 5748 | 1 | AAG | 4.90 |
| 841 | 3 | AAA | 4.36 | 2078 | 1 | CUG | 3.98 | 5749 | 1 | UCA | 3.09 |
| 842 | 2 | AUG | 4.74 | 2079 | 1 | UAA | 6.50 | 5750 | 1 | UAU | 4.67 |
| 844 | 2 | CUU | 4.63 | 2080 | 1 | UUC | 4.65 | 5751 | 1 | AUU | 4.78 |
| 845 | 10 | AUU | 3.37 | 2081 | 1 | CAG | 5.74 | 5753 | 1 | AUU | 6.73 |
| 846 | 2 | UGA | 4.14 | 2082 | 1 | AGA | 4.58 | 5754 | 1 | AUG | 4.03 |
| 847 | 2 | UCU | 5.37 | 2083 | 1 | UUC | 3.12 | 5755 | 1 | AAU | 4.64 |
| 848 | 2 | CUU | 4.43 | 2084 | 1 | GGA | 4.39 | 5756 | 1 | UCA | 5.95 |
| 851 | 2 | GAC | 3.79 | 2085 | 1 | UCC | 4.39 | 5757 | 1 | AAU | 6.54 |
| 852 | 2 | AAG | 7.01 | 2087 | 1 | UUU | 4.53 | 5758 | 1 | ACU | 4.66 |
| 853 | 2 | CUU | 3.98 | 2088 | 1 | UCU | 5.48 | 5759 | 1 | AAU | 4.85 |
| 854 | 6 | GAG | 4.25 | 2089 | 1 | AUU | 4.91 | 5760 | 1 | UUA | 9.21 |
| 856 | 2 | AGA | 3.80 | 2090 | 1 | AUU | 4.85 | 5762 | 1 | UCA | 5.07 |
| 857 | 3 | AAA | 4.95 | 2091 | 1 | GAG | 8.14 | 5764 | 1 | UUG | 4.08 |
| 858 | 6 | UUU | 3.82 | 2092 | 1 | CCA | 4.85 | 5765 | 1 | AAC | 3.61 |
| 859 | 2 | GAU | 5.15 | 2093 | 1 | AUU | 6.90 | 5766 | 1 | GAA | 4.58 |
| 860 | 3 | AAU | 4.16 | 2094 | 1 | UGG | 5.56 | 5767 | 1 | ACA | 4.50 |
| 861 | 2 | AUG | 5.76 | 2095 | 1 | AUA | 6.90 | 5768 | 1 | UUC | 5.81 |
| 862 | 4 | UUC | 4.19 | 2096 | 1 | CGC | 3.38 | 5769 | 1 | AUU | 4.12 |
| 868 | 2 | AUA | 3.76 | 2097 | 1 | GGA | 5.65 | 5771 | 1 | GGA | 7.41 |
| 869 | 2 | UUG | 3.33 | 2098 | 1 | GAG | 4.17 | 5772 | 1 | AAU | 4.24 |
| 898 | 2 | UGC | 4.09 | 2099 | 1 | AGC | 6.67 | 5773 | 1 | AAA | 5.78 |
| 902 | 12 | GAU | 3.80 | 2100 | 1 | AAG | 4.58 | 5775 | 1 | UCU | 5.34 |
| 904 | 2 | UUG | 4.55 | 2101 | 1 | CAA | 4.67 | 5776 | 1 | UCA | 4.62 |
| 946 | 3 | UGU | 4.44 | 2102 | 1 | CCG | 6.42 | 5777 | 1 | AAU | 4.08 |
| 947 | 3 | GCA | 5.42 | 2103 | 1 | CCG | 5.48 | 5778 | 1 | UCG | 5.69 |
| 948 | 2 | UUC | 7.26 | 2104 | 1 | CGG | 7.62 | 5779 | 1 | CCA | 4.02 |
| 949 | 3 | GAA | 4.55 | 2105 | 1 | AUU | 9.64 | 5780 | 1 | UGA | 7.03 |
| 950 | 4 | UUU | 4.07 | 2106 | 1 | UUC | 4.65 | 5781 | 1 | UGA | 6.10 |
| 951 | 3 | UUU | 4.26 | 2107 | 1 | AUC | 7.41 | 5782 | 1 | UUC | 4.26 |
| 952 | 5 | AUU | 3.73 | 2120 | 1 | UCU | 5.48 | 5783 | 1 | ACG | 7.14 |
| 1023 | 5 | UUG | 4.31 | 2122 | 1 | GCG | 3.77 | 5784 | 1 | AAU | 5.45 |
| 1024 | 2 | UCA | 3.68 | 2199 | 1 | AUA | 5.56 | 5785 | 1 | UGU | 4.93 |
| 1026 | 2 | UCU | 4.13 | 2218 | 1 | AUU | 5.41 | 5786 | 1 | AUG | 5.00 |
| 1027 | 2 | AGA | 5.63 | 2589 | 1 | AUU | 4.92 | 5788 | 1 | GAU | 6.03 |
| 1028 | 3 | UCU | 3.03 | 2591 | 1 | AUU | 6.34 | 5789 | 1 | UUC | 4.62 |
| 1030 | 10 | UGC | 5.32 | 2594 | 1 | UUC | 5.06 | 5790 | 1 | ACU | 4.82 |
| 1031 | 2 | UUG | 4.68 | 2595 | 1 | UUA | 5.00 | 5791 | 1 | GCA | 4.70 |
| 1033 | 5 | AUG | 5.38 | 2596 | 1 | UUU | 5.21 | 5792 | 1 | UCG | 6.49 |
| 1063 | 8 | GAA | 2.55 | 2597 | 1 | AUU | 7.48 | 5793 | 1 | CUU | 5.13 |
| 1078 | 2 | AUU | 5.62 | 2598 | 1 | UUU | 5.95 | 5794 | 1 | AAA | 2.75 |
| 1082 | 2 | CUC | 5.88 | 2599 | 1 | ACA | 5.23 | 5795 | 1 | CGA | 4.11 |
| 1094 | 3 | CAC | 4.49 | 2601 | 1 | UUU | 5.99 | 5796 | 1 | UUG | 4.68 |
| 1095 | 2 | UUG | 4.53 | 2603 | 1 | CAA | 6.01 | 5797 | 1 | UGC | 6.06 |
| 1119 | 2 | GUG | 6.15 | 2604 | 1 | UUA | 4.88 | 5798 | 1 | UUG | 3.82 |
| 1120 | 2 | UCC | 3.89 | 2605 | 1 | UUA | 8.48 | 5799 | 1 | UUU | 6.45 |
| 1122 | 3 | ACA | 3.38 | 2607 | 1 | AUG | 7.08 | 5800 | 1 | AUC | 5.38 |
| 1127 | 2 | AUU | 4.47 | 2608 | 1 | UCU | 5.66 | 5801 | 1 | AUC | 6.33 |
| 1128 | 2 | UAU | 3.50 | 2611 | 1 | UUG | 4.73 | 5802 | 1 | GGA | 6.25 |
| 1130 | 2 | UAA | 4.44 | 2612 | 1 | UAU | 5.30 | 5803 | 1 | AUA | 4.60 |
| 1134 | 2 | CAA | 4.10 | 2613 | 1 | GGU | 8.72 | 5804 | 1 | UGC | 5.51 |
| 1135 | 2 | GGA | 3.74 | 2614 | 1 | UCG | 7.45 | 5805 | 1 | GGC | 4.18 |
| 1139 | 2 | CAU | 4.40 | 2616 | 1 | UUU | 4.07 | 5806 | 1 | AAG | 3.70 |
| 1140 | 2 | UUU | 4.95 | 2620 | 1 | UGA | 3.76 | 5807 | 1 | UAA | 3.97 |
| 1144 | 2 | GGC | 4.79 | 2621 | 1 | AUU | 5.70 | 5808 | 1 | GAU | 7.59 |
| 1151 | 2 | CGG | 7.32 | 2622 | 1 | AUU | 4.55 | 5809 | 1 | GCG | 6.35 |
| 1161 | 2 | ACA | 6.59 | 2623 | 1 | UGU | 5.65 | 5810 | 1 | AUG | 4.69 |
| 1219 | 4 | CCU | 3.83 | 2624 | 1 | CGA | 6.45 | 5811 | 1 | AAA | 3.90 |
| 1220 | 2 | CUC | 3.90 | 2625 | 1 | CGU | 5.49 | 5812 | 1 | AUU | 8.06 |
| 1222 | 5 | GUG | 2.93 | 2626 | 1 | UUA | 5.14 | 5813 | 1 | AAG | 3.70 |
| 1223 | 10 | GCU | 2.82 | 2627 | 1 | AAC | 5.10 | 5814 | 1 | AGU | 4.63 |
| 1310 | 4 | GGC | 4.37 | 2628 | 1 | AUU | 4.76 | 5815 | 1 | AUG | 8.04 |
| 1311 | 3 | CAG | 3.86 | 2631 | 1 | AUU | 6.27 | 5816 | 1 | UAG | 7.14 |
| 1312 | 2 | AAU | 3.65 | 2633 | 1 | UAA | 4.24 | 5817 | 1 | UUU | 3.95 |
| 1313 | 2 | UGG | 4.37 | 2634 | 1 | UCA | 4.85 | 5818 | 1 | UUG | 5.08 |
| 1314 | 2 | UUA | 4.12 | 2635 | 1 | UAU | 5.88 | 5819 | 1 | GGC | 6.35 |
| 1319 | 2 | UAA | 5.83 | 2636 | 1 | AUU | 4.17 | 5820 | 1 | AUU | 5.95 |
| 1428 | 7 | AUU | 3.48 | 2637 | 1 | UAU | 7.26 | 5821 | 1 | GCG | 6.07 |
| 1432 | 4 | CCG | 4.46 | 2639 | 1 | AAU | 4.12 | 5822 | 1 | GCA | 4.81 |
| 1435 | 3 | AAA | 5.45 | 2640 | 1 | AUU | 4.48 | 5823 | 1 | AAU | 5.61 |
| 1436 | 2 | UAC | 4.23 | 2641 | 1 | AAA | 5.19 | 5824 | 1 | UUA | 7.46 |
| 1437 | 2 | CGC | 5.62 | 2642 | 1 | UUU | 5.49 | 5825 | 1 | UUA | 6.03 |
| 1440 | 2 | UGG | 3.98 | 2644 | 1 | ACA | 3.65 | 5826 | 1 | CUC | 5.12 |
| 1444 | 5 | UAU | 5.09 | 2645 | 1 | AUU | 5.24 | 5827 | 1 | UUG | 3.41 |
| 1446 | 7 | UCU | 4.11 | 2648 | 1 | AUU | 5.07 | 5828 | 1 | UAA | 5.41 |
| 1507 | 10 | CAU | 3.84 | 2649 | 1 | AAG | 4.76 | 5829 | 1 | UCA | 4.62 |
| 1508 | 4 | UUG | 5.01 | 2650 | 1 | UUU | 6.18 | 5830 | 1 | UUU | 5.13 |
| 1509 | 5 | UCU | 4.01 | 2651 | 1 | AUU | 8.16 | 5831 | 1 | UAA | 8.33 |
| 1510 | 6 | UGU | 3.92 | 2654 | 1 | AUU | 7.41 | 5832 | 1 | CAU | 5.49 |
| 1511 | 3 | CAU | 4.31 | 2657 | 1 | AUU | 5.41 | 5833 | 1 | CUC | 11.67 |
| 1512 | 3 | AAU | 5.58 | 2658 | 1 | AUU | 5.26 | 5834 | 1 | GAA | 5.08 |
| 1513 | 3 | AUC | 4.69 | 2660 | 1 | AGC | 9.68 | 5835 | 1 | AAA | 4.21 |
| 1514 | 3 | UUU | 5.48 | 2661 | 1 | AUU | 5.07 | 5836 | 1 | AUG | 4.49 |
| 1515 | 3 | UUU | 5.41 | 2662 | 1 | AAA | 5.83 | 5837 | 1 | AUG | 5.21 |
| 1516 | 4 | CUU | 4.29 | 2663 | 1 | UGG | 9.65 | 5995 | 1 | AUA | 4.96 |
| 1520 | 18 | UGA | 5.56 | 2665 | 1 | GAA | 6.19 | 5996 | 1 | AAC | 4.00 |
| 1521 | 2 | AUU | 5.61 | 2666 | 1 | UUU | 5.18 | 5997 | 1 | CUU | 4.17 |
| 1523 | 2 | UCA | 4.81 | 2668 | 1 | AAU | 7.50 | 5999 | 1 | AAU | 5.76 |
| 1535 | 2 | CUU | 4.38 | 2672 | 1 | UAU | 6.15 | 6021 | 1 | UCA | 4.27 |
| 1846 | 5 | CCG | 8.24 | 2674 | 1 | AUG | 4.42 | 6028 | 1 | AUG | 3.82 |
| 1858 | 2 | GGA | 3.80 | 2675 | 1 | UUU | 7.51 | 6029 | 1 | UUG | 6.03 |
| 1861 | 15 | GUU | 4.25 | 2677 | 1 | AAA | 5.88 | 6030 | 1 | ACC | 3.54 |
| 1862 | 7 | GUA | 3.52 | 2678 | 1 | AGA | 3.73 | 6031 | 1 | AGC | 4.23 |
| 1863 | 5 | UGA | 2.94 | 2864 | 1 | UGC | 4.95 | 6032 | 1 | UGU | 6.00 |
| 1878 | 2 | AAA | 4.04 | 2865 | 1 | AUC | 4.95 | 6033 | 1 | AAC | 3.64 |
| 1882 | 8 | AUC | 3.67 | 2866 | 1 | AAC | 3.96 | 6034 | 1 | UAU | 6.30 |
| 1883 | 2 | CCG | 3.59 | 2867 | 1 | UGU | 5.94 | 6035 | 1 | UUU | 6.59 |
| 1885 | 2 | UCU | 4.00 | 2868 | 1 | UUG | 4.94 | 6036 | 1 | AUA | 4.52 |
| 1886 | 10 | GAU | 6.28 | 2869 | 1 | AGG | 5.59 | 6103 | 1 | UGU | 6.84 |
| 1887 | 2 | AGU | 5.56 | 2870 | 1 | ACU | 4.97 | 6104 | 1 | AUA | 8.45 |
| 1888 | 2 | UUA | 6.46 | 2872 | 1 | GAU | 3.89 | 6106 | 1 | CAA | 4.76 |
| 1919 | 5 | UUC | 3.59 | 2874 | 1 | UGU | 7.92 | 6107 | 1 | UGU | 5.45 |
| 2086 | 3 | UUU | 4.49 | 2875 | 1 | UAU | 6.17 | 6109 | 1 | CAU | 8.89 |
| 2108 | 2 | UGU | 7.30 | 2876 | 1 | UUG | 5.52 | 6110 | 1 | UCA | 5.74 |
| 2109 | 2 | GAG | 6.10 | 2877 | 1 | UGC | 3.32 | 6111 | 1 | AUG | 3.75 |
| 2111 | 47 | UUA | 3.69 | 2878 | 1 | AUA | 6.93 | 6112 | 1 | UUU | 6.03 |
| 2112 | 2 | AAA | 5.23 | 2879 | 1 | AAU | 3.65 | 6113 | 1 | CGA | 4.94 |
| 2118 | 36 | UCC | 3.78 | 2880 | 1 | GUA | 4.44 | 6114 | 1 | UUU | 4.35 |
| 2119 | 3 | UUU | 6.49 | 2905 | 1 | UGU | 4.94 | 6115 | 1 | CAU | 5.71 |
| 2121 | 2 | UCC | 3.50 | 2913 | 1 | GCA | 5.94 | 6116 | 1 | UCA | 6.70 |
| 2275 | 9 | UUG | 4.04 | 2916 | 1 | CCA | 4.35 | 6117 | 1 | AAC | 5.10 |
| 2585 | 4 | AUU | 6.06 | 2918 | 1 | AAG | 4.24 | 6118 | 1 | AUU | 5.17 |
| 2586 | 2 | GAC | 7.12 | 2919 | 1 | GGG | 6.88 | 6138 | 1 | AAU | 5.99 |
| 2587 | 7 | CAU | 6.15 | 2920 | 1 | CAA | 6.19 | 6139 | 1 | AUU | 8.87 |
| 2588 | 2 | ACA | 5.30 | 2921 | 1 | UAA | 7.92 | 6141 | 1 | AUU | 6.67 |
| 2590 | 10 | AAU | 6.76 | 2922 | 1 | AAU | 4.29 | 6142 | 1 | CUA | 3.95 |
| 2592 | 66 | AUU | 3.76 | 2923 | 1 | AUA | 5.14 | 6144 | 1 | ACU | 4.41 |
| 2593 | 5 | CAU | 5.64 | 2924 | 1 | GCC | 8.33 | 6147 | 1 | AUG | 3.85 |
| 2600 | 5 | UUG | 5.61 | 2925 | 1 | CUU | 6.67 | 6150 | 1 | AAG | 7.41 |
| 2602 | 2 | AAU | 5.31 | 2926 | 1 | CAC | 6.67 | 6153 | 1 | UGA | 5.77 |
| 2606 | 5 | AAU | 4.42 | 2927 | 1 | CGC | 6.00 | 6155 | 1 | AUU | 4.92 |
| 2609 | 2 | AUG | 5.25 | 2928 | 1 | UUU | 6.00 | 6156 | 1 | AUG | 4.67 |
| 2610 | 2 | UUG | 3.76 | 2929 | 1 | AAU | 5.22 | 6157 | 1 | UAG | 6.25 |
| 2615 | 3 | UUU | 7.93 | 2930 | 1 | GCG | 5.56 | 6159 | 1 | AAU | 4.85 |
| 2617 | 3 | UUA | 6.55 | 2931 | 1 | UAU | 6.49 | 6160 | 1 | UAU | 5.26 |
| 2618 | 2 | UUA | 8.69 | 2932 | 1 | AUG | 4.29 | 6162 | 1 | AUU | 6.58 |
| 2619 | 2 | CAA | 3.36 | 2934 | 1 | UUU | 6.61 | 6163 | 1 | AUG | 5.83 |
| 2629 | 8 | GUU | 4.98 | 2936 | 1 | UUC | 4.44 | 6166 | 1 | UCU | 4.62 |
| 2630 | 25 | AAU | 6.14 | 2937 | 1 | AUG | 3.31 | 6167 | 1 | ACC | 5.00 |
| 2632 | 3 | AUU | 5.23 | 2938 | 1 | UUU | 6.00 | 6168 | 1 | CUG | 5.31 |
| 2638 | 2 | AAU | 4.83 | 2939 | 1 | ACA | 4.80 | 6169 | 1 | AAU | 5.63 |
| 2643 | 2 | AAU | 4.31 | 2947 | 1 | AUG | 4.27 | 6170 | 1 | UCU | 5.71 |
| 2646 | 2 | AUU | 5.34 | 2948 | 1 | UUG | 4.61 | 6171 | 1 | AUU | 5.63 |
| 2647 | 3 | UUG | 4.12 | 3433 | 1 | AUG | 4.94 | 6172 | 1 | UAA | 5.71 |
| 2652 | 13 | CAU | 3.67 | 3435 | 1 | UGA | 4.64 | 6173 | 1 | AGC | 3.57 |
| 2653 | 4 | UGA | 4.89 | 3436 | 1 | UUU | 6.98 | 6174 | 1 | CAA | 4.38 |
| 2655 | 15 | UAA | 8.61 | 3437 | 1 | CAA | 3.73 | 6175 | 1 | AAG | 3.57 |
| 2656 | 5 | UUG | 6.29 | 3438 | 1 | UUU | 6.44 | 6176 | 1 | UGC | 7.46 |
| 2659 | 11 | UUC | 4.88 | 3439 | 1 | UAA | 4.29 | 6177 | 1 | AAG | 3.99 |
| 2664 | 2 | AAA | 4.98 | 3441 | 1 | GAA | 4.43 | 6178 | 1 | AAG | 4.08 |
| 2667 | 2 | AUC | 7.41 | 3442 | 1 | UUU | 6.59 | 6179 | 1 | UUG | 4.30 |
| 2669 | 2 | AGU | 7.27 | 3443 | 1 | AAU | 5.93 | 6180 | 1 | GAA | 6.12 |
| 2670 | 7 | UUU | 4.09 | 3445 | 1 | AAG | 4.57 | 6181 | 1 | CGG | 6.58 |
| 2671 | 10 | UAA | 5.58 | 3446 | 1 | GAA | 3.78 | 6182 | 1 | CAA | 3.45 |
| 2673 | 2 | UUG | 5.15 | 3447 | 1 | UGA | 5.00 | 6183 | 1 | AGU | 5.26 |
| 2676 | 6 | AAU | 6.99 | 3448 | 1 | UUG | 5.05 | 6184 | 1 | CGG | 10.31 |
| 2679 | 3 | ACU | 4.48 | 3449 | 1 | AGA | 5.41 | 6185 | 1 | ACG | 3.74 |
| 2680 | 5 | UCU | 5.24 | 3476 | 1 | AAC | 4.39 | 6186 | 1 | UCU | 7.74 |
| 2863 | 3 | AAU | 4.53 | 3508 | 1 | UCU | 4.79 | 6187 | 1 | AAC | 4.94 |
| 2871 | 2 | UAG | 4.25 | 3509 | 1 | UGA | 6.16 | 6188 | 1 | UGG | 5.77 |
| 2873 | 3 | UUU | 6.10 | 3510 | 1 | UUG | 5.48 | 6189 | 1 | UGC | 8.23 |
| 2907 | 4 | GCC | 6.30 | 3511 | 1 | AAA | 4.82 | 6190 | 1 | UCU | 4.27 |
| 2912 | 2 | UCU | 4.81 | 3512 | 1 | UUA | 4.26 | 6191 | 1 | AUU | 7.50 |
| 2933 | 2 | AAU | 6.25 | 3513 | 1 | UUG | 8.33 | 6192 | 1 | UGU | 7.02 |
| 2949 | 3 | UUG | 4.98 | 3514 | 1 | UUA | 6.25 | 6193 | 1 | UGC | 3.61 |
| 2950 | 3 | UGG | 5.65 | 3515 | 1 | AAA | 4.60 | 6194 | 1 | GCC | 4.60 |
| 3434 | 2 | UAA | 3.41 | 3516 | 1 | UUC | 5.66 | 6195 | 1 | CGU | 4.83 |
| 3440 | 3 | UUG | 3.83 | 3517 | 1 | ACC | 5.95 | 6196 | 1 | GAG | 5.88 |
| 3444 | 2 | AUC | 5.58 | 3518 | 1 | UAA | 5.00 | 6197 | 1 | ACA | 4.35 |
| 3522 | 3 | GCU | 5.54 | 3519 | 1 | AAU | 4.81 | 6198 | 1 | UUG | 5.56 |
| 3627 | 8 | UGU | 3.30 | 3520 | 1 | UAU | 6.96 | 6199 | 1 | UCA | 5.41 |
| 3629 | 3 | UUU | 4.92 | 3521 | 1 | UUG | 6.98 | 6200 | 1 | CAU | 5.24 |
| 3630 | 2 | UGA | 4.90 | 3623 | 1 | AAG | 4.65 | 6201 | 1 | UGA | 5.56 |
| 3631 | 4 | UAA | 5.61 | 3624 | 1 | UCU | 4.46 | 6202 | 1 | UGU | 6.19 |
| 3633 | 2 | UUC | 5.14 | 3625 | 1 | UUG | 4.52 | 6203 | 1 | UUC | 3.68 |
| 3701 | 2 | UUG | 3.86 | 3626 | 1 | AGU | 4.00 | 6204 | 1 | CUG | 5.38 |
| 3704 | 3 | AGA | 3.60 | 3628 | 1 | AUU | 3.33 | 6205 | 1 | GUU | 7.23 |
| 3709 | 2 | AUU | 7.29 | 3632 | 1 | AAG | 3.77 | 6206 | 1 | GGC | 5.88 |
| 3712 | 2 | AUC | 7.25 | 3634 | 1 | AUG | 3.67 | 6207 | 1 | GGC | 5.56 |
| 3932 | 2 | CUU | 5.92 | 3635 | 1 | AUU | 4.55 | 6208 | 1 | CAG | 4.72 |
| 3980 | 2 | GGC | 5.21 | 3636 | 1 | AUU | 5.67 | 6209 | 1 | AUG | 4.00 |
| 4221 | 2 | AGU | 4.42 | 3637 | 1 | AUU | 6.93 | 6210 | 1 | AAC | 4.49 |
| 4227 | 2 | AUU | 6.59 | 3638 | 1 | AUA | 5.85 | 6211 | 1 | CAA | 3.82 |
| 4228 | 2 | CGU | 3.47 | 3639 | 1 | AAA | 5.14 | 6212 | 1 | ACA | 5.62 |
| 4239 | 2 | UUU | 5.30 | 3640 | 1 | UAU | 5.00 | 6213 | 1 | AUU | 5.05 |
| 4240 | 2 | UUU | 4.16 | 3693 | 1 | CAA | 4.76 | 6214 | 1 | CGG | 4.02 |
| 4243 | 2 | AUU | 5.31 | 3694 | 1 | AUU | 8.45 | 6218 | 1 | AAA | 3.76 |
| 4245 | 2 | AUU | 5.55 | 3695 | 1 | UUG | 3.57 | 6219 | 1 | CCG | 4.82 |
| 4248 | 3 | AAA | 5.86 | 3696 | 1 | GAU | 3.94 | 6220 | 1 | AUA | 5.61 |
| 4343 | 2 | CUU | 5.05 | 3697 | 1 | AAG | 4.21 | 6221 | 1 | AGC | 4.92 |
| 4352 | 2 | AAU | 4.49 | 3698 | 1 | UGC | 4.90 | 6222 | 1 | AGC | 5.05 |
| 4359 | 2 | GUU | 5.66 | 3699 | 1 | AGA | 5.19 | 6223 | 1 | UUC | 5.56 |
| 4364 | 2 | GAA | 7.49 | 3700 | 1 | UGA | 5.13 | 6225 | 1 | AAU | 5.26 |
| 4368 | 2 | AAG | 5.38 | 3702 | 1 | AUG | 4.67 | 6226 | 1 | GAU | 6.02 |
| 4371 | 3 | UGA | 5.62 | 3703 | 1 | UUG | 4.88 | 6227 | 1 | UAU | 4.38 |
| 4372 | 2 | AAU | 6.62 | 3705 | 1 | UAU | 5.93 | 6228 | 1 | AUU | 4.94 |
| 4374 | 2 | AAG | 4.93 | 3706 | 1 | AUG | 5.62 | 6229 | 1 | GGG | 5.81 |
| 4376 | 4 | GCA | 3.76 | 3707 | 1 | AUU | 5.15 | 6230 | 1 | AAA | 3.98 |
| 4378 | 2 | UCU | 4.89 | 3708 | 1 | AUC | 3.16 | 6231 | 1 | UGG | 4.67 |
| 4380 | 2 | AUC | 4.92 | 3710 | 1 | CUU | 5.19 | 6233 | 1 | UUG | 5.07 |
| 4387 | 5 | UCA | 6.39 | 3711 | 1 | ACC | 4.51 | 6235 | 1 | UUU | 4.67 |
| 4393 | 2 | AAA | 3.99 | 3933 | 1 | AAU | 4.55 | 6245 | 1 | AUA | 5.00 |
| 4401 | 2 | AAG | 4.55 | 3946 | 1 | UGA | 4.61 | 6246 | 1 | GGA | 8.33 |
| 4413 | 2 | AUU | 7.12 | 3947 | 1 | UUA | 7.98 | 6247 | 1 | AGC | 3.88 |
| 4414 | 3 | GCU | 4.47 | 3948 | 1 | UUA | 7.19 | 6248 | 1 | AGG | 4.85 |
| 4415 | 2 | AUC | 4.27 | 3949 | 1 | GUU | 5.65 | 6250 | 1 | AUC | 3.97 |
| 4416 | 3 | UUG | 3.72 | 3950 | 1 | UUU | 6.96 | 6251 | 1 | CCA | 5.14 |
| 5014 | 2 | ACA | 6.38 | 3951 | 1 | UCU | 6.08 | 6252 | 1 | UAA | 6.98 |
| 5020 | 3 | UGA | 5.59 | 3952 | 1 | AAG | 4.29 | 6253 | 1 | AUU | 5.06 |
| 5037 | 7 | AAG | 4.31 | 3953 | 1 | UGC | 3.96 | 6254 | 1 | UAU | 4.44 |
| 5038 | 2 | AUU | 7.31 | 3954 | 1 | UUC | 5.75 | 6255 | 1 | GUU | 5.88 |
| 5048 | 2 | AUU | 4.00 | 3979 | 1 | AAG | 5.26 | 6256 | 1 | ACA | 4.12 |
| 5049 | 7 | AUU | 3.60 | 3981 | 1 | AUA | 5.56 | 6257 | 1 | UAA | 7.14 |
| 5079 | 2 | AUU | 4.19 | 3982 | 1 | UCC | 3.20 | 6258 | 1 | CUU | 4.65 |
| 5143 | 2 | UGU | 4.27 | 4222 | 1 | UUG | 4.82 | 6259 | 1 | UUU | 5.56 |
| 5148 | 3 | AUG | 7.73 | 4223 | 1 | AAA | 5.52 | 6260 | 1 | CUC | 4.90 |
| 5157 | 2 | CUU | 5.38 | 4224 | 1 | AAA | 4.41 | 6261 | 1 | AAU | 4.05 |
| 5163 | 2 | AUU | 4.27 | 4225 | 1 | AUU | 5.33 | 6262 | 1 | AUU | 4.84 |
| 5167 | 2 | AGA | 3.72 | 4226 | 1 | UUG | 4.82 | 6263 | 1 | AAA | 5.61 |
| 5174 | 5 | UUU | 3.18 | 4229 | 1 | UUG | 4.90 | 6264 | 1 | AUG | 5.77 |
| 5175 | 2 | UUC | 3.96 | 4230 | 1 | AAU | 6.72 | 6265 | 1 | AAU | 6.86 |
| 5179 | 2 | UUG | 3.20 | 4231 | 1 | AAG | 3.39 | 6269 | 1 | CAU | 4.21 |
| 5180 | 2 | AGU | 5.09 | 4232 | 1 | AUU | 4.14 | 6270 | 1 | AUU | 4.51 |
| 5181 | 3 | UUA | 3.74 | 4233 | 1 | UGU | 4.88 | 6271 | 1 | UCU | 6.50 |
| 5185 | 13 | UUC | 5.20 | 4234 | 1 | AUU | 4.93 | 6272 | 1 | AAA | 6.67 |
| 5205 | 4 | GGA | 4.33 | 4235 | 1 | UGU | 5.59 | 6273 | 1 | AAA | 6.50 |
| 5206 | 2 | UCA | 4.03 | 4236 | 1 | AAU | 6.04 | 6275 | 1 | CUU | 5.61 |
| 5208 | 4 | UGU | 5.02 | 4237 | 1 | AUA | 6.35 | 6276 | 1 | UUG | 7.34 |
| 5211 | 2 | UGA | 3.69 | 4238 | 1 | UUU | 6.56 | 6277 | 1 | UGU | 5.21 |
| 5216 | 2 | AGU | 6.87 | 4241 | 1 | CAU | 3.24 | 6278 | 1 | AAU | 5.10 |
| 5224 | 2 | CGU | 3.87 | 4242 | 1 | GAA | 4.42 | 6279 | 1 | AAC | 4.58 |
| 5225 | 8 | CUU | 3.09 | 4244 | 1 | UUG | 7.48 | 6280 | 1 | UUG | 6.67 |
| 5229 | 2 | GUU | 4.49 | 4246 | 1 | UUU | 5.41 | 6281 | 1 | GAG | 6.88 |
| 5235 | 2 | UGG | 4.46 | 4247 | 1 | AAU | 7.14 | 6282 | 1 | AAA | 2.92 |
| 5236 | 5 | UUG | 5.22 | 4249 | 1 | UUU | 5.23 | 6283 | 1 | AAA | 3.91 |
| 5241 | 3 | AUU | 5.18 | 4250 | 1 | UUG | 5.60 | 6284 | 1 | UUU | 4.12 |
| 5267 | 15 | UAG | 4.84 | 4340 | 1 | CUG | 4.62 | 6285 | 1 | AAG | 6.17 |
| 5268 | 2 | AAG | 4.46 | 4341 | 1 | AAC | 3.45 | 6286 | 1 | AUU | 5.10 |
| 5269 | 2 | CAU | 4.91 | 4342 | 1 | UUU | 4.48 | 6287 | 1 | AAG | 5.19 |
| 5270 | 2 | UUU | 6.17 | 4344 | 1 | AAG | 5.04 | 6289 | 1 | GAA | 5.71 |
| 5271 | 5 | GUU | 10.73 | 4345 | 1 | CUU | 5.32 | 6290 | 1 | UGA | 5.21 |
| 5272 | 6 | UUG | 7.88 | 4346 | 1 | AAG | 3.39 | 6292 | 1 | AUC | 6.67 |
| 5274 | 2 | AUA | 4.57 | 4347 | 1 | AAG | 5.67 | 6293 | 1 | AUA | 4.63 |
| 5281 | 6 | AAU | 4.11 | 4348 | 1 | AUG | 6.21 | 6294 | 1 | AAU | 5.88 |
| 5284 | 8 | AUU | 4.34 | 4349 | 1 | AUA | 4.43 | 6295 | 1 | UUU | 3.64 |
| 5285 | 3 | UUG | 6.61 | 4350 | 1 | AUU | 7.50 | 6296 | 1 | AUA | 5.34 |
| 5286 | 2 | ACA | 6.21 | 4351 | 1 | UUC | 6.06 | 6299 | 1 | AUU | 8.25 |
| 5287 | 2 | AAU | 5.50 | 4353 | 1 | AGU | 5.08 | 6300 | 1 | AUA | 6.37 |
| 5289 | 2 | ACU | 6.97 | 4354 | 1 | AUC | 5.33 | 6421 | 1 | AGA | 5.04 |
| 5291 | 3 | GAU | 9.80 | 4355 | 1 | AAU | 5.00 | 6422 | 1 | UAU | 6.12 |
| 5292 | 2 | GAU | 7.25 | 4356 | 1 | AAA | 5.00 | 6423 | 1 | CGG | 9.21 |
| 5294 | 3 | UCA | 4.54 | 4357 | 1 | UCG | 4.55 | 6424 | 1 | UGC | 4.55 |
| 5295 | 4 | GGC | 4.55 | 4358 | 1 | AUU | 4.22 | 6427 | 1 | UCA | 4.81 |
| 5298 | 4 | GAU | 7.11 | 4360 | 1 | CGU | 7.50 | 6428 | 1 | CUC | 4.96 |
| 5303 | 14 | GGA | 3.13 | 4361 | 1 | AAG | 6.35 | 6429 | 1 | UCA | 4.88 |
| 5304 | 2 | CAU | 6.59 | 4362 | 1 | UUU | 4.55 | 6430 | 1 | CUU | 5.04 |
| 5337 | 2 | UUU | 4.57 | 4363 | 1 | AUC | 5.22 | 6431 | 1 | AAU | 6.12 |
| 5380 | 2 | UUA | 8.03 | 4365 | 1 | GAA | 7.29 | 6432 | 1 | UUU | 7.62 |
| 5387 | 2 | GGU | 4.11 | 4366 | 1 | AAA | 4.20 | 6434 | 1 | UUG | 4.49 |
| 5512 | 2 | AAU | 5.22 | 4367 | 1 | AAU | 4.89 | 6435 | 1 | UCA | 4.63 |
| 5516 | 2 | UGG | 4.28 | 4369 | 1 | GGA | 7.59 | 6436 | 1 | UGG | 4.94 |
| 5534 | 2 | CGA | 4.49 | 4370 | 1 | GAC | 6.06 | 6441 | 1 | ACG | 4.12 |
| 5539 | 2 | GCU | 6.58 | 4373 | 1 | CGU | 5.77 | 6442 | 1 | AGG | 5.71 |
| 5554 | 3 | AUG | 5.71 | 4375 | 1 | CAC | 7.35 | 6443 | 1 | AAA | 4.27 |
| 5559 | 2 | AUU | 4.65 | 4377 | 1 | AUG | 6.55 | 6444 | 1 | UUA | 6.97 |
| 5564 | 3 | GCC | 4.10 | 4379 | 1 | UAU | 4.76 | 6446 | 1 | UUA | 3.15 |
| 5565 | 7 | AAU | 4.26 | 4381 | 1 | AUU | 4.35 | 6447 | 1 | UUG | 4.93 |
| 5568 | 7 | UAU | 4.00 | 4382 | 1 | AUA | 4.09 | 6448 | 1 | AUU | 3.45 |
| 5630 | 2 | UUC | 4.17 | 4383 | 1 | AUC | 7.09 | 6449 | 1 | GUU | 5.68 |
| 5635 | 4 | AAC | 5.75 | 4384 | 1 | AAU | 4.55 | 6451 | 1 | AUU | 7.69 |
| 5638 | 2 | AAA | 3.53 | 4385 | 1 | AAU | 7.29 | 6452 | 1 | CAA | 7.63 |
| 5642 | 2 | UGU | 3.22 | 4386 | 1 | AGA | 5.45 | 6454 | 1 | UAA | 4.49 |
| 5643 | 2 | UCU | 5.42 | 4388 | 1 | UUU | 5.00 | 6455 | 1 | AAU | 7.93 |
| 5645 | 6 | AAA | 5.09 | 4389 | 1 | CGG | 5.44 | 6456 | 1 | GAU | 5.43 |
| 5649 | 2 | AUA | 7.43 | 4390 | 1 | UCG | 7.58 | 6458 | 1 | UUU | 4.52 |
| 5654 | 3 | CUU | 4.06 | 4391 | 1 | AAG | 5.04 | 6460 | 1 | AUU | 6.08 |
| 5674 | 2 | CAA | 5.57 | 4392 | 1 | GAA | 5.45 | 6461 | 1 | AGU | 4.80 |
| 5741 | 5 | AUU | 5.51 | 4394 | 1 | AAA | 5.56 | 6463 | 1 | CAU | 7.53 |
| 5743 | 2 | AAA | 6.79 | 4395 | 1 | GCU | 5.50 | 6464 | 1 | UAU | 8.47 |
| 5745 | 2 | CAU | 5.36 | 4396 | 1 | UAG | 5.17 | 6465 | 1 | UAA | 8.18 |
| 5752 | 2 | AAU | 5.04 | 4397 | 1 | GAA | 3.18 | 6466 | 1 | AAU | 5.88 |
| 5761 | 2 | UUG | 4.06 | 4398 | 1 | UUU | 6.15 | 6467 | 1 | GCU | 5.00 |
| 5763 | 3 | UAU | 5.92 | 4399 | 1 | UUA | 4.64 | 6468 | 1 | CCA | 4.64 |
| 5770 | 2 | UUG | 3.55 | 4400 | 1 | GAA | 9.55 | 6469 | 1 | UUC | 3.57 |
| 5774 | 2 | CAG | 5.56 | 4402 | 1 | AUU | 5.68 | 6470 | 1 | UUG | 5.80 |
| 5998 | 2 | UUG | 5.33 | 4403 | 1 | ACA | 7.69 | 6471 | 1 | AAA | 4.00 |
| 6019 | 2 | UUG | 5.22 | 4404 | 1 | AUC | 4.84 | 6472 | 1 | AAU | 3.75 |
| 6020 | 2 | CUU | 3.84 | 4405 | 1 | UUA | 4.76 | 6473 | 1 | CAU | 4.17 |
| 6022 | 2 | AAC | 3.48 | 4406 | 1 | AGA | 4.84 | 6474 | 1 | AUC | 3.81 |
| 6023 | 2 | AAU | 3.83 | 4407 | 1 | AUU | 4.58 | 6475 | 1 | AAU | 4.40 |
| 6024 | 3 | UCU | 3.94 | 4408 | 1 | CCA | 4.96 | 6477 | 1 | UUC | 6.50 |
| 6025 | 6 | UUG | 4.40 | 4409 | 1 | GUU | 6.56 | 6478 | 1 | UUG | 5.07 |
| 6026 | 2 | UUG | 5.58 | 4410 | 1 | UGA | 7.04 | 6479 | 1 | CAA | 6.03 |
| 6027 | 2 | GAA | 4.26 | 4411 | 1 | AUU | 8.93 | 6480 | 1 | AAC | 4.90 |
| 6105 | 2 | UGU | 4.26 | 4412 | 1 | UAU | 5.68 | 6482 | 1 | UGG | 5.38 |
| 6108 | 7 | GAU | 4.02 | 4992 | 1 | AAU | 4.55 | 6483 | 1 | AUU | 6.03 |
| 6135 | 11 | AUU | 4.59 | 4993 | 1 | GAU | 7.14 | 6484 | 1 | AAU | 7.41 |
| 6136 | 2 | UAU | 3.42 | 4994 | 1 | AUC | 7.69 | 6485 | 1 | AUU | 5.56 |
| 6137 | 2 | AUA | 5.90 | 4995 | 1 | AAG | 4.44 | 6725 | 1 | GAU | 4.72 |
| 6140 | 4 | UUG | 4.03 | 4996 | 1 | UUU | 4.21 | 7126 | 1 | CGC | 3.97 |
| 6143 | 2 | UGA | 4.90 | 4997 | 1 | CAU | 4.55 | 7128 | 1 | AAU | 6.25 |
| 6145 | 6 | UGC | 3.28 | 4998 | 1 | CUU | 4.62 | 7129 | 1 | AUC | 8.06 |
| 6146 | 2 | UCA | 5.56 | 5012 | 1 | ACU | 4.03 | 7130 | 1 | AUG | 5.56 |
| 6148 | 2 | GUU | 6.30 | 5013 | 1 | UCU | 4.40 | 7485 | 1 | AUU | 4.01 |
| 6149 | 3 | UAU | 5.19 | 5015 | 1 | UGU | 8.09 | 7487 | 1 | AAA | 5.84 |
| 6151 | 9 | AUU | 6.89 | 5016 | 1 | AAU | 4.21 | 7488 | 1 | AUU | 4.91 |
| 6152 | 2 | AUU | 5.80 | 5017 | 1 | AAU | 6.18 | 7489 | 1 | AUA | 4.83 |
| 6154 | 2 | GGU | 6.83 | 5018 | 1 | AGU | 4.00 | 7490 | 1 | AAA | 5.06 |
| 6158 | 3 | GAG | 3.58 | 5019 | 1 | UUU | 4.96 | 7491 | 1 | AAU | 4.88 |
| 6161 | 4 | UAU | 6.34 | 5021 | 1 | AGA | 6.43 | 7493 | 1 | AAU | 7.54 |
| 6164 | 2 | UUU | 5.39 | 5022 | 1 | AUG | 5.13 | 7494 | 1 | UUU | 7.62 |
| 6217 | 2 | UAU | 7.12 | 5023 | 1 | UUG | 6.67 | 7497 | 1 | AUG | 4.97 |
| 6224 | 3 | AUA | 4.28 | 5024 | 1 | AGA | 4.44 | 7498 | 1 | CAC | 5.08 |
| 6232 | 2 | AUA | 3.84 | 5025 | 1 | UAU | 4.52 | 7499 | 1 | UAA | 8.22 |
| 6234 | 2 | UUG | 3.81 | 5026 | 1 | AAA | 4.66 | 7500 | 1 | UCG | 9.84 |
| 6249 | 2 | GCC | 8.54 | 5027 | 1 | AAA | 4.97 | 7501 | 1 | AAA | 6.50 |
| 6266 | 3 | AAA | 7.65 | 5028 | 1 | AUC | 3.31 | 7502 | 1 | UUA | 7.03 |
| 6267 | 3 | AUU | 3.49 | 5029 | 1 | GAU | 5.24 | 7503 | 1 | AAU | 7.18 |
| 6274 | 2 | AUA | 5.57 | 5030 | 1 | GAA | 10.77 | 7505 | 1 | CUU | 5.56 |
| 6288 | 3 | UUA | 6.11 | 5031 | 1 | UAA | 9.93 | 7506 | 1 | AUG | 6.93 |
| 6291 | 3 | UAU | 4.00 | 5032 | 1 | GAA | 5.96 | 7507 | 1 | AUU | 5.43 |
| 6297 | 2 | GCG | 5.67 | 5033 | 1 | AAA | 5.96 | 7508 | 1 | CUU | 7.27 |
| 6425 | 5 | AUG | 3.94 | 5034 | 1 | AAA | 3.31 | 7509 | 1 | UUU | 6.06 |
| 6426 | 2 | GAA | 8.16 | 5035 | 1 | AAA | 4.64 | 7511 | 1 | AUA | 4.58 |
| 6437 | 2 | GCU | 9.76 | 5036 | 1 | AUA | 4.64 | 7512 | 1 | AUG | 6.82 |
| 6438 | 2 | UUG | 4.92 | 5039 | 1 | AUU | 6.62 | 7513 | 1 | AUU | 6.80 |
| 6439 | 2 | UGA | 4.18 | 5040 | 1 | AUA | 5.96 | 7514 | 1 | AUG | 4.21 |
| 6440 | 4 | CGA | 6.14 | 5041 | 1 | UUG | 3.97 | 7516 | 1 | AUU | 3.62 |
| 6445 | 2 | AAU | 5.25 | 5042 | 1 | AAG | 4.64 | 7517 | 1 | ACC | 4.20 |
| 6450 | 2 | GUC | 3.83 | 5043 | 1 | ACC | 4.64 | 7518 | 1 | UUU | 5.96 |
| 6457 | 2 | AGA | 4.63 | 5044 | 1 | AAA | 3.31 | 7519 | 1 | AAA | 4.88 |
| 6459 | 2 | AAG | 4.85 | 5050 | 1 | ACC | 3.36 | 7520 | 1 | AUC | 5.32 |
| 6462 | 6 | AAG | 3.23 | 5051 | 1 | UUU | 5.71 | 7521 | 1 | UGU | 4.95 |
| 6476 | 3 | AUG | 5.05 | 5052 | 1 | CGG | 4.57 | 7522 | 1 | UUU | 6.45 |
| 7120 | 2 | AUU | 5.52 | 5053 | 1 | UCC | 4.50 | 7524 | 1 | GUC | 6.67 |
| 7121 | 8 | UGG | 4.06 | 5054 | 1 | GAU | 5.15 | 7525 | 1 | UUU | 4.05 |
| 7122 | 5 | UCU | 4.91 | 5055 | 1 | CUG | 4.98 | 7527 | 1 | GCG | 6.49 |
| 7123 | 2 | CUA | 3.83 | 5056 | 1 | UUC | 5.56 | 7528 | 1 | UUG | 6.98 |
| 7124 | 2 | AUU | 6.57 | 5057 | 1 | AAA | 7.89 | 7529 | 1 | UUU | 3.45 |
| 7125 | 2 | CAA | 4.67 | 5058 | 1 | UUU | 6.12 | 7530 | 1 | CCU | 3.67 |
| 7127 | 2 | AAU | 5.49 | 5059 | 1 | CUC | 5.26 | 7531 | 1 | AAA | 5.03 |
| 7484 | 2 | AUU | 4.95 | 5060 | 1 | CGG | 4.90 | 7534 | 1 | UUG | 6.32 |
| 7486 | 2 | CUU | 5.81 | 5061 | 1 | AUC | 3.33 | 7535 | 1 | UGU | 5.75 |
| 7492 | 3 | UUU | 4.28 | 5062 | 1 | AUU | 4.03 | 7537 | 1 | UCA | 3.79 |
| 7495 | 2 | AAC | 4.74 | 5063 | 1 | CUU | 6.41 | 7538 | 1 | GGA | 6.25 |
| 7496 | 2 | AUU | 6.21 | 5064 | 1 | CAU | 3.74 | 7539 | 1 | UUU | 7.64 |
| 7504 | 2 | AAU | 6.68 | 5065 | 1 | UUC | 4.19 | 7541 | 1 | GCC | 5.20 |
| 7510 | 2 | GCG | 6.41 | 5066 | 1 | CUU | 4.95 | 7542 | 1 | UUG | 5.84 |
| 7523 | 2 | CUG | 4.43 | 5067 | 1 | ACU | 4.65 | 7543 | 1 | UUU | 4.40 |
| 7526 | 8 | GCU | 5.36 | 5068 | 1 | ACC | 6.58 | 7544 | 1 | AAU | 7.41 |
| 7532 | 2 | GGU | 5.29 | 5069 | 1 | UAU | 6.17 | 7545 | 1 | AUU | 4.92 |
| 7533 | 2 | AAU | 4.59 | 5070 | 1 | AAC | 4.03 | 7546 | 1 | UGG | 7.19 |
| 7536 | 2 | AGA | 5.06 | 5071 | 1 | AUA | 6.06 | 7692 | 1 | UUU | 3.77 |
| 7540 | 2 | UGA | 5.16 | 5072 | 1 | UCG | 5.26 | 7693 | 1 | UCC | 3.74 |
| 7696 | 4 | UUC | 5.09 | 5073 | 1 | AAU | 5.61 | 7694 | 1 | UAG | 4.79 |
| 7708 | 2 | UUU | 4.03 | 5074 | 1 | CCG | 11.76 | 7695 | 1 | AAA | 3.49 |
| 7717 | 3 | AUU | 5.35 | 5075 | 1 | CCG | 6.35 | 7697 | 1 | GAU | 5.86 |
| 7723 | 2 | UUG | 3.89 | 5076 | 1 | UUG | 4.37 | 7698 | 1 | UAG | 4.20 |
| 7724 | 2 | AUG | 4.75 | 5077 | 1 | UCG | 5.49 | 7699 | 1 | AAU | 7.09 |
| 7725 | 2 | CAU | 3.81 | 5078 | 1 | GCA | 3.91 | 7700 | 1 | UGG | 2.97 |
| 7726 | 2 | AAU | 4.11 | 5080 | 1 | ACA | 4.49 | 7701 | 1 | AAU | 4.35 |
| 7729 | 2 | UGG | 8.33 | 5081 | 1 | AUA | 4.00 | 7707 | 1 | GGC | 3.91 |
| 7748 | 2 | AUA | 4.74 | 5082 | 1 | GCG | 6.47 | 7709 | 1 | GGC | 4.11 |
| 7768 | 2 | GCG | 9.90 | 5083 | 1 | UUC | 5.26 | 7710 | 1 | CUA | 4.37 |
| 7784 | 2 | GUA | 8.13 | 5084 | 1 | GUA | 4.65 | 7711 | 1 | AUU | 4.56 |
| 7817 | 2 | UCU | 4.35 | 5085 | 1 | UUU | 5.47 | 7712 | 1 | UAA | 4.41 |
| 7980 | 2 | UGA | 5.95 | 5086 | 1 | AAA | 4.76 | 7713 | 1 | AUG | 3.59 |
| 7982 | 2 | AUA | 6.82 | 5137 | 1 | UUU | 4.20 | 7714 | 1 | UAU | 5.75 |
| 7984 | 4 | AUA | 5.65 | 5138 | 1 | UAG | 5.26 | 7715 | 1 | AAG | 4.69 |
| 7990 | 2 | UUU | 6.10 | 5139 | 1 | UAU | 6.16 | 7716 | 1 | GAG | 4.47 |
| 7991 | 3 | AAU | 8.51 | 5140 | 1 | UGG | 3.79 | 7718 | 1 | AUU | 4.14 |
| 7993 | 4 | UUA | 7.32 | 5141 | 1 | UGA | 4.32 | 7719 | 1 | UUG | 4.32 |
| 7994 | 2 | AUA | 8.68 | 5142 | 1 | UCA | 6.02 | 7720 | 1 | UCG | 4.46 |
| 7996 | 3 | AUU | 6.84 | 5144 | 1 | CCG | 4.26 | 7721 | 1 | AUA | 6.25 |
| 7997 | 3 | UGC | 3.38 | 5145 | 1 | AAA | 4.88 | 7722 | 1 | UCC | 5.61 |
| 8001 | 2 | AUU | 7.21 | 5146 | 1 | ACC | 5.95 | 7727 | 1 | AGU | 6.12 |
| 8005 | 3 | UUA | 10.63 | 5147 | 1 | CAU | 3.55 | 7728 | 1 | AUU | 3.81 |
| 8007 | 2 | UUU | 5.84 | 5149 | 1 | UUU | 3.99 | 7730 | 1 | UUA | 3.13 |
| 8008 | 2 | UUU | 6.36 | 5150 | 1 | AGC | 6.33 | 7731 | 1 | ACA | 3.40 |
| 8011 | 2 | AAA | 5.23 | 5151 | 1 | UGA | 5.37 | 7732 | 1 | CGG | 6.67 |
| 8024 | 2 | UUG | 8.34 | 5152 | 1 | AAA | 4.63 | 7733 | 1 | AUG | 3.77 |
| 8032 | 7 | GGU | 5.95 | 5153 | 1 | AUA | 4.37 | 7734 | 1 | AAC | 3.50 |
| 8038 | 2 | UUG | 6.06 | 5154 | 1 | GCA | 4.96 | 7735 | 1 | CGG | 4.32 |
| 8041 | 2 | AUU | 6.34 | 5155 | 1 | UUU | 4.81 | 7736 | 1 | UGA | 5.03 |
| 8139 | 5 | CUG | 5.17 | 5156 | 1 | GCA | 3.50 | 7737 | 1 | UUU | 3.10 |
| 384 | 1 | CCA | 6.67 | 5158 | 1 | AUG | 4.14 | 7738 | 1 | UUU | 4.88 |
| 401 | 1 | AAA | 3.60 | 5159 | 1 | AAC | 5.26 | 7739 | 1 | CUG | 5.24 |
| 404 | 1 | GUU | 7.33 | 5160 | 1 | AUU | 5.53 | 7740 | 1 | AUG | 4.07 |
| 406 | 1 | AAA | 5.50 | 5161 | 1 | CCA | 4.71 | 7741 | 1 | CAU | 4.00 |
| 407 | 1 | AAA | 6.08 | 5162 | 1 | AUU | 6.59 | 7742 | 1 | AUU | 4.62 |
| 420 | 1 | UUU | 7.00 | 5164 | 1 | ACU | 3.61 | 7743 | 1 | UUU | 5.80 |
| 435 | 1 | UUG | 4.55 | 5165 | 1 | AAU | 4.82 | 7744 | 1 | CCA | 3.10 |
| 438 | 1 | UAU | 4.84 | 5166 | 1 | AAU | 3.53 | 7745 | 1 | AAG | 6.98 |
| 440 | 1 | GAU | 5.00 | 5169 | 1 | UUG | 4.76 | 7746 | 1 | AUU | 3.37 |
| 443 | 1 | AUA | 6.08 | 5170 | 1 | AAU | 3.47 | 7747 | 1 | UGU | 4.20 |
| 480 | 1 | UUG | 5.71 | 5171 | 1 | AUU | 5.84 | 7749 | 1 | GAG | 6.16 |
| 775 | 1 | UUU | 6.50 | 5172 | 1 | AUC | 3.08 | 7750 | 1 | CAU | 4.19 |
| 776 | 1 | GAU | 5.08 | 5173 | 1 | AUA | 3.87 | 7751 | 1 | UGG | 6.10 |
| 777 | 1 | UAA | 4.63 | 5176 | 1 | AUG | 6.76 | 7752 | 1 | AUG | 4.07 |
| 778 | 1 | ACA | 3.62 | 5177 | 1 | GUU | 4.14 | 7753 | 1 | UCU | 5.35 |
| 779 | 1 | UGA | 6.13 | 5178 | 1 | GCC | 3.27 | 7754 | 1 | GGA | 4.07 |
| 780 | 1 | AUC | 5.17 | 5182 | 1 | AAU | 3.72 | 7755 | 1 | UGU | 4.23 |
| 782 | 1 | AAA | 5.41 | 5183 | 1 | AUA | 5.26 | 7756 | 1 | UUG | 8.60 |
| 816 | 1 | AUG | 5.19 | 5184 | 1 | UUU | 4.56 | 7757 | 1 | UAG | 3.18 |
| 817 | 1 | UCA | 6.02 | 5198 | 1 | GAG | 6.45 | 7758 | 1 | AAU | 3.66 |
| 826 | 1 | UAU | 4.49 | 5199 | 1 | UUG | 4.86 | 7759 | 1 | UGG | 3.57 |
| 830 | 1 | AGA | 4.10 | 5200 | 1 | AAG | 6.90 | 7760 | 1 | GGC | 5.71 |
| 832 | 1 | AAG | 4.50 | 5201 | 1 | UGA | 4.44 | 7761 | 1 | CCA | 3.92 |
| 836 | 1 | UUU | 4.15 | 5202 | 1 | UUA | 6.00 | 7762 | 1 | CAA | 3.83 |
| 843 | 1 | CAU | 4.39 | 5203 | 1 | AUA | 5.97 | 7763 | 1 | GAU | 5.77 |
| 849 | 1 | AUU | 4.76 | 5204 | 1 | CUU | 5.81 | 7764 | 1 | AUC | 5.41 |
| 850 | 1 | AUA | 4.52 | 5207 | 1 | UUU | 4.31 | 7765 | 1 | UCC | 2.90 |
| 855 | 1 | UUU | 5.15 | 5209 | 1 | UUG | 6.09 | 7766 | 1 | GGC | 9.46 |
| 863 | 1 | AUA | 6.60 | 5210 | 1 | UUG | 5.98 | 7767 | 1 | AGC | 6.31 |
| 864 | 1 | UGA | 6.52 | 5212 | 1 | UGG | 4.71 | 7769 | 1 | GGC | 7.32 |
| 865 | 1 | AAU | 7.04 | 5213 | 1 | UUC | 6.19 | 7770 | 1 | AGA | 4.84 |
| 866 | 1 | AUU | 3.97 | 5214 | 1 | AUU | 5.05 | 7771 | 1 | UUG | 5.11 |
| 867 | 1 | ACA | 5.08 | 5215 | 1 | CUU | 5.81 | 7772 | 1 | GGA | 5.15 |
| 870 | 1 | AAU | 4.71 | 5217 | 1 | UUG | 4.61 | 7773 | 1 | ACG | 5.38 |
| 893 | 1 | CUU | 5.00 | 5218 | 1 | AGA | 7.04 | 7774 | 1 | UGG | 6.25 |
| 894 | 1 | AAU | 6.82 | 5219 | 1 | AGG | 4.76 | 7775 | 1 | UUA | 5.60 |
| 895 | 1 | UUG | 4.50 | 5221 | 1 | AAU | 5.00 | 7776 | 1 | AUG | 4.08 |
| 897 | 1 | CUU | 5.38 | 5222 | 1 | UUU | 4.72 | 7777 | 1 | UGU | 6.62 |
| 899 | 1 | UUC | 3.60 | 5223 | 1 | AAU | 4.81 | 7778 | 1 | CGC | 5.44 |
| 900 | 1 | AGA | 5.00 | 5226 | 1 | UUC | 8.14 | 7779 | 1 | AUG | 4.08 |
| 901 | 1 | UAA | 6.33 | 5227 | 1 | AAU | 5.02 | 7780 | 1 | UUU | 5.00 |
| 903 | 1 | CGG | 3.77 | 5228 | 1 | AAA | 3.90 | 7781 | 1 | UCC | 2.90 |
| 905 | 1 | GCC | 3.41 | 5230 | 1 | UUG | 5.56 | 7782 | 1 | AUG | 4.62 |
| 906 | 1 | GAU | 3.47 | 5231 | 1 | AAG | 4.76 | 7783 | 1 | AUG | 4.74 |
| 907 | 1 | CGC | 6.01 | 5232 | 1 | CAU | 6.11 | 7785 | 1 | AUG | 7.50 |
| 908 | 1 | UCA | 3.04 | 5233 | 1 | UGG | 6.06 | 7786 | 1 | UGU | 7.34 |
| 909 | 1 | GGC | 4.46 | 5234 | 1 | AUU | 4.58 | 7787 | 1 | GGU | 4.46 |
| 910 | 1 | CCG | 7.05 | 5237 | 1 | AUU | 5.98 | 7812 | 1 | AAU | 5.41 |
| 911 | 1 | AUG | 8.43 | 5238 | 1 | AAA | 4.44 | 7813 | 1 | UUG | 4.47 |
| 912 | 1 | GGC | 4.26 | 5239 | 1 | AGA | 3.60 | 7814 | 1 | AAA | 4.74 |
| 913 | 1 | CUU | 3.21 | 5240 | 1 | AAU | 9.72 | 7815 | 1 | CAA | 4.92 |
| 914 | 1 | UGC | 3.47 | 5242 | 1 | CAU | 6.06 | 7816 | 1 | AAU | 5.83 |
| 915 | 1 | CGC | 4.20 | 5243 | 1 | AAU | 5.41 | 7818 | 1 | AUU | 5.17 |
| 916 | 1 | GCC | 3.22 | 5244 | 1 | AAC | 4.04 | 7819 | 1 | AAU | 5.73 |
| 917 | 1 | GCC | 3.12 | 5245 | 1 | ACA | 4.29 | 7820 | 1 | AAU | 5.00 |
| 918 | 1 | GCG | 3.35 | 5246 | 1 | UUU | 7.14 | 7821 | 1 | CAU | 5.36 |
| 919 | 1 | CGC | 3.40 | 5247 | 1 | UCU | 5.80 | 7822 | 1 | AUU | 6.31 |
| 1025 | 1 | UGU | 4.29 | 5248 | 1 | UUG | 5.00 | 7823 | 1 | AUU | 6.22 |
| 1029 | 1 | UUG | 5.13 | 5249 | 1 | UUU | 4.90 | 7824 | 1 | AAU | 5.22 |
| 1032 | 1 | CCA | 5.51 | 5250 | 1 | AAU | 4.31 | 7825 | 1 | AAU | 4.78 |
| 1034 | 1 | UGC | 4.14 | 5251 | 1 | UUG | 3.85 | 7826 | 1 | AAU | 5.05 |
| 1035 | 1 | UGU | 5.29 | 5252 | 1 | ACU | 6.32 | 7827 | 1 | AAU | 5.15 |
| 1036 | 1 | AUU | 4.52 | 5253 | 1 | GAU | 4.60 | 7828 | 1 | AAU | 5.41 |
| 1037 | 1 | CUA | 6.25 | 5254 | 1 | AAG | 5.32 | 7829 | 1 | UGG | 4.27 |
| 1038 | 1 | CAU | 5.83 | 5255 | 1 | AUG | 5.68 | 7830 | 1 | UUG | 5.17 |
| 1039 | 1 | CUU | 4.62 | 5256 | 1 | AAU | 4.92 | 7831 | 1 | ACC | 3.40 |
| 1040 | 1 | UUC | 4.23 | 5257 | 1 | AAG | 4.51 | 7832 | 1 | UGU | 7.14 |
| 1041 | 1 | AAA | 4.23 | 5258 | 1 | UCA | 4.55 | 7833 | 1 | CUA | 5.75 |
| 1042 | 1 | UUG | 3.76 | 5259 | 1 | AUU | 4.26 | 7834 | 1 | AAU | 5.21 |
| 1043 | 1 | GAA | 5.43 | 5260 | 1 | CAA | 8.16 | 7835 | 1 | GGA | 5.88 |
| 1044 | 1 | UUU | 6.06 | 5261 | 1 | AAU | 6.06 | 7836 | 1 | AAA | 4.32 |
| 1045 | 1 | GCA | 4.14 | 5262 | 1 | AUU | 4.71 | 7837 | 1 | AAA | 5.42 |
| 1046 | 1 | UCA | 5.00 | 5263 | 1 | AAC | 5.77 | 7838 | 1 | AAU | 3.95 |
| 1047 | 1 | CCU | 4.14 | 5264 | 1 | UUG | 5.50 | 7839 | 1 | AUU | 4.92 |
| 1048 | 1 | UGU | 4.83 | 5265 | 1 | GUU | 5.36 | 7840 | 1 | AAG | 4.76 |
| 1049 | 1 | UCG | 4.32 | 5266 | 1 | CCA | 6.49 | 7841 | 1 | UUU | 5.13 |
| 1050 | 1 | UCA | 4.64 | 5273 | 1 | UUA | 6.41 | 7842 | 1 | AAG | 5.26 |
| 1051 | 1 | GAA | 3.55 | 5275 | 1 | AUG | 3.11 | 7979 | 1 | AAG | 4.08 |
| 1052 | 1 | UUG | 5.20 | 5276 | 1 | UGG | 5.05 | 7981 | 1 | AAA | 4.33 |
| 1053 | 1 | ACU | 6.61 | 5277 | 1 | AAG | 9.01 | 7983 | 1 | UAA | 10.00 |
| 1054 | 1 | CUG | 4.08 | 5278 | 1 | AAA | 5.50 | 7985 | 1 | UCG | 5.77 |
| 1055 | 1 | UGU | 3.90 | 5279 | 1 | UUA | 7.22 | 7986 | 1 | UAA | 9.20 |
| 1056 | 1 | AAA | 4.14 | 5280 | 1 | AAU | 6.08 | 7987 | 1 | GAU | 8.77 |
| 1057 | 1 | AGA | 5.17 | 5282 | 1 | AUU | 7.09 | 7988 | 1 | AAU | 4.69 |
| 1058 | 1 | UGU | 3.52 | 5283 | 1 | UAU | 8.74 | 7989 | 1 | AAU | 6.06 |
| 1059 | 1 | AUC | 2.79 | 5288 | 1 | GAA | 5.63 | 7992 | 1 | AAA | 4.29 |
| 1060 | 1 | GAA | 4.14 | 5290 | 1 | UUU | 5.75 | 7995 | 1 | UAA | 6.47 |
| 1061 | 1 | AUG | 4.55 | 5293 | 1 | GAA | 9.09 | 7998 | 1 | AAG | 4.03 |
| 1062 | 1 | UUG | 4.89 | 5296 | 1 | AAU | 5.45 | 7999 | 1 | ACG | 6.00 |
| 1064 | 1 | GGG | 4.35 | 5297 | 1 | UAU | 7.46 | 8000 | 1 | UUU | 7.02 |
| 1065 | 1 | CUG | 4.25 | 5299 | 1 | AAU | 7.78 | 8002 | 1 | AAA | 5.21 |
| 1066 | 1 | UAC | 4.20 | 5300 | 1 | UGA | 4.15 | 8003 | 1 | AAA | 6.45 |
| 1067 | 1 | UUG | 4.76 | 5301 | 1 | UUG | 4.79 | 8004 | 1 | UUU | 6.35 |
| 1068 | 1 | CAA | 4.41 | 5302 | 1 | UUU | 5.58 | 8006 | 1 | UAU | 5.12 |
| 1069 | 1 | UAU | 5.74 | 5338 | 1 | AUU | 5.26 | 8009 | 1 | UUU | 7.14 |
| 1070 | 1 | AAC | 4.46 | 5339 | 1 | AGC | 3.08 | 8010 | 1 | AAU | 5.39 |
| 1071 | 1 | GAA | 4.44 | 5340 | 1 | AUU | 6.82 | 8012 | 1 | GCG | 4.42 |
| 1072 | 1 | AAU | 7.77 | 5368 | 1 | AGA | 4.14 | 8013 | 1 | UUU | 6.76 |
| 1073 | 1 | AAU | 3.98 | 5369 | 1 | UGA | 5.22 | 8014 | 1 | UGU | 9.72 |
| 1074 | 1 | AAC | 6.92 | 5370 | 1 | AUU | 4.58 | 8015 | 1 | AAA | 5.48 |
| 1075 | 1 | UUG | 5.63 | 5371 | 1 | CUC | 4.04 | 8016 | 1 | UAU | 5.71 |
| 1076 | 1 | UGU | 4.41 | 5372 | 1 | UGU | 6.15 | 8017 | 1 | UCA | 4.76 |
| 1077 | 1 | AAA | 3.29 | 5373 | 1 | AGA | 7.27 | 8018 | 1 | AUC | 5.45 |
| 1079 | 1 | UGU | 3.97 | 5374 | 1 | AAU | 4.09 | 8019 | 1 | AAA | 4.55 |
| 1080 | 1 | GAU | 5.94 | 5375 | 1 | AAU | 4.71 | 8020 | 1 | ACA | 5.75 |
| 1081 | 1 | UCU | 4.62 | 5376 | 1 | UUC | 4.79 | 8021 | 1 | UUA | 4.57 |
| 1083 | 1 | CAU | 5.26 | 5377 | 1 | UUC | 4.10 | 8022 | 1 | AAU | 10.53 |
| 1084 | 1 | AUG | 3.42 | 5378 | 1 | AAA | 5.86 | 8023 | 1 | AAA | 5.30 |
| 1085 | 1 | GAU | 3.96 | 5379 | 1 | AUU | 5.95 | 8025 | 1 | AAU | 7.25 |
| 1086 | 1 | AGC | 5.88 | 5381 | 1 | CGC | 5.45 | 8026 | 1 | AAU | 5.13 |
| 1087 | 1 | UUU | 4.15 | 5382 | 1 | AAA | 3.64 | 8027 | 1 | UAU | 7.84 |
| 1088 | 1 | UCU | 5.21 | 5383 | 1 | GGC | 5.45 | 8028 | 1 | AUU | 5.59 |
| 1089 | 1 | AUC | 4.92 | 5384 | 1 | CCG | 8.18 | 8029 | 1 | AUU | 6.67 |
| 1090 | 1 | CUC | 4.00 | 5385 | 1 | CCG | 4.55 | 8030 | 1 | UUG | 7.02 |
| 1091 | 1 | UGC | 4.68 | 5386 | 1 | GUC | 5.45 | 8031 | 1 | UUG | 6.90 |
| 1092 | 1 | AUG | 3.85 | 5388 | 1 | AAA | 9.09 | 8033 | 1 | AAA | 8.57 |
| 1093 | 1 | CCA | 5.56 | 5389 | 1 | CAG | 4.55 | 8034 | 1 | AAA | 5.23 |
| 1096 | 1 | UCU | 4.04 | 5484 | 1 | UUG | 4.45 | 8035 | 1 | AUU | 3.64 |
| 1097 | 1 | UUG | 3.41 | 5485 | 1 | UUG | 4.24 | 8036 | 1 | AUU | 6.52 |
| 1098 | 1 | ACG | 3.96 | 5486 | 1 | UUG | 5.75 | 8037 | 1 | CUU | 5.41 |
| 1099 | 1 | AAG | 3.73 | 5487 | 1 | AUG | 5.00 | 8039 | 1 | AUC | 5.13 |
| 1100 | 1 | UUC | 4.39 | 5488 | 1 | AUU | 4.69 | 8040 | 1 | AAU | 7.59 |
| 1101 | 1 | UGC | 5.21 | 5489 | 1 | UGC | 3.52 | 8042 | 1 | UGA | 5.83 |
| 1102 | 1 | UGC | 6.73 | 5490 | 1 | AGG | 4.67 | 8043 | 1 | AAA | 5.60 |
| 1103 | 1 | AAG | 3.03 | 5491 | 1 | UUG | 4.27 | 8044 | 1 | AUG | 5.88 |
| 1104 | 1 | UUC | 6.19 | 5492 | 1 | AAU | 5.20 | 8045 | 1 | AUU | 6.74 |
| 1105 | 1 | AGC | 5.22 | 5493 | 1 | CCA | 3.89 | 8046 | 1 | AAU | 8.43 |
| 1106 | 1 | AAA | 6.09 | 5494 | 1 | UUU | 4.24 | 8047 | 1 | UUU | 5.75 |
| 1107 | 1 | CUG | 5.56 | 5495 | 1 | CUU | 4.17 | 8048 | 1 | GAU | 8.70 |
| 1108 | 1 | AGC | 3.36 | 5496 | 1 | CGU | 4.65 | 8049 | 1 | AUG | 7.59 |
| 1109 | 1 | AUU | 4.27 | 5497 | 1 | UCU | 5.07 | 8050 | 1 | AGG | 4.85 |
| 1110 | 1 | AGG | 4.08 | 5498 | 1 | UUG | 8.28 | 8051 | 1 | AUU | 6.98 |
| 1111 | 1 | UGU | 3.62 | 5499 | 1 | UUC | 5.60 | 8122 | 1 | UUC | 4.17 |
| 1112 | 1 | ACU | 4.21 | 5500 | 1 | GAU | 5.68 | 8123 | 1 | UUG | 5.10 |
| 1113 | 1 | AAG | 5.43 | 5501 | 1 | AGG | 4.10 | 8124 | 1 | AGC | 5.04 |
| 1114 | 1 | AUC | 3.88 | 5502 | 1 | UAC | 4.93 | 8125 | 1 | AUG | 5.19 |
| 1115 | 1 | AGC | 4.81 | 5503 | 1 | AUU | 5.08 | 8126 | 1 | UUU | 4.81 |
| 1117 | 1 | ACC | 4.24 | 5504 | 1 | UUG | 5.10 | 8127 | 1 | CAA | 3.60 |
| 1118 | 1 | UUU | 5.00 | 5505 | 1 | UUU | 3.85 | 8128 | 1 | CGA | 4.62 |
| 1121 | 1 | UUA | 5.10 | 5506 | 1 | UGA | 5.41 | 8129 | 1 | UAU | 6.17 |
| 1123 | 1 | UGA | 4.59 | 5507 | 1 | AUG | 3.67 | 8130 | 1 | UUG | 3.61 |
| 1124 | 1 | ACG | 3.88 | 5508 | 1 | UCU | 5.23 | 8131 | 1 | UCA | 5.56 |
| 1125 | 1 | AAU | 4.26 | 5509 | 1 | AUU | 4.66 | 8132 | 1 | AUU | 5.45 |
| 1129 | 1 | CCA | 5.00 | 5510 | 1 | UCA | 5.60 | 8133 | 1 | UCC | 4.94 |
| 1131 | 1 | GGU | 5.00 | 5511 | 1 | UGG | 4.69 | 8134 | 1 | AAA | 5.94 |
| 1133 | 1 | AGU | 3.33 | 5513 | 1 | AUA | 4.79 | 8135 | 1 | AAA | 7.94 |
| 1136 | 1 | GUA | 4.67 | 5514 | 1 | CUU | 5.77 | 8136 | 1 | AAU | 7.32 |
| 1137 | 1 | AGU | 6.08 | 5515 | 1 | GUU | 5.65 | 8137 | 1 | AAA | 6.15 |
| 1138 | 1 | UCU | 5.15 | 5517 | 1 | AAU | 4.85 | 8138 | 1 | AUA | 4.90 |
| 1142 | 1 | CCC | 6.25 | 5518 | 1 | AUU | 4.29 | 8140 | 1 | AAU | 4.90 |
| 1143 | 1 | UAU | 5.59 | 5519 | 1 | GCU | 5.71 | 8141 | 1 | UAG | 5.33 |
| 1145 | 1 | GUC | 4.31 | 5521 | 1 | UGA | 3.80 | 8142 | 1 | UUA | 5.21 |
| 1146 | 1 | ACA | 6.06 | 5522 | 1 | UCC | 4.42 | 8143 | 1 | AUC | 6.78 |
| 1147 | 1 | GCC | 7.07 | 5523 | 1 | AUU | 4.76 | 8144 | 1 | AUU | 6.12 |
| 1148 | 1 | UCC | 4.29 | 5524 | 1 | AAU | 4.23 | 8145 | 1 | AUU | 4.81 |
| 1149 | 1 | GUC | 4.39 | 5525 | 1 | AUG | 3.85 | 8146 | 1 | AUU | 8.45 |
| 1150 | 1 | CGC | 4.27 | 5526 | 1 | CUA | 4.74 | 8147 | 1 | UGA | 9.26 |
| 1152 | 1 | CUU | 5.38 | 5527 | 1 | AUC | 3.56 | 8148 | 1 | CUG | 8.77 |
| 1153 | 1 | AUU | 3.73 | 5528 | 1 | AUC | 4.11 | 8149 | 1 | UAG | 5.63 |
| 1154 | 1 | UGU | 4.13 | 5529 | 1 | AUU | 4.53 | 8150 | 1 | UGA | 5.48 |
| 1155 | 1 | AGG | 5.00 | 5530 | 1 | AUG | 6.78 | 8151 | 1 | AUA | 5.75 |
| 1156 | 1 | CAG | 4.87 | 5531 | 1 | UGA | 5.68 | 8152 | 1 | ACU | 6.33 |
| 1157 | 1 | CAG | 5.84 | 5532 | 1 | UAU | 4.55 | 8153 | 1 | GUA | 6.32 |
| 1158 | 1 | AGC | 4.22 | 5533 | 1 | UGC | 3.62 | 8154 | 1 | CUC | 8.33 |
| 1159 | 1 | GCC | 4.78 | 5535 | 1 | UUU | 5.79 | 8155 | 1 | AUA | 4.55 |
| 1160 | 1 | AGC | 3.66 | 5536 | 1 | UAU | 5.48 |  |  |  |  |

^a^SSR signature repeats per 100 nucleotides of precursor miRNA. R value is reported as average for miRNA families with more than one member.
