# Supplementary material for: Computational prediction of miRNAs and their targets in Phaseolus vulgaris using simple sequence repeat signatures
Source: BMC Plant Biol. 2015 Jun 12;15:140. doi: 10.1186/s12870-015-0516-3 (PMC4464996; doi:10.1186/s12870-015-0516-3)
Supplement: Supplementary file 2 — Predicted miRNAs of P. vulgaris. [file 12870_2015_516_MOESM2_ESM.docx]

# Table S2 - Predicted miRNAs of *P. vulgaris*.

| Family | Member miRNA(s) | Mature Sequence | SSR^a^ | LM^b^ | LP^c^ | AU^d^ | MFEI^e^ | Npb^f^ | NQ^g^ | ND^h^ | R^i^ |
| --- | --- | --- | --- | --- | --- | --- | --- | --- | --- | --- | --- |
| 156 | miR156a | GACAGAAGAGAGAAAGCAG | AGA | 19 | 143 | 58.74 | 1.02 | 0.37 | 0.14 | 0.06 | 7.69 |
|  | miR156b | GUGCUCUCUCUCUUCUGUCAAC |  | 22 | 110 | 56.36 | 1.06 | 0.35 | 0.14 | 0.05 | 4.55 |
|  | miR156c | GAUGUCAGAUAGAGCAAUGAGC |  | 22 | 324 | 64.20 | 0.68 | 0.30 | 0.28 | 0.08 | 4.01 |
|  | miR156d | UGGCAGAAGGAAAGGGAGC |  | 19 | 67 | 58.21 | 0.76 | 0.33 | 0.11 | 0.04 | 4.48 |
| 159 | miR159a* | UUGGACUGAAGGGAGCUCCU | UUG | 20 | 206 | 60.19 | 0.97 | 0.37 | 0.18 | 0.07 | 2.91 |
|  | miR159b | GACUUAGAGUGCUGGGAACU |  | 20 | 413 | 62.95 | 0.74 | 0.32 | 0.32 | 0.11 | 2.91 |
|  | miR159c | UUGCAUAUCUCCGGAGCUU |  | 19 | 454 | 54.41 | 0.69 | 0.32 | 0.24 | 0.09 | 2.86 |
|  | miR159d | ACUUGGUGUGCAAGGAACUC |  | 20 | 55 | 56.36 | 0.80 | 0.31 | 0.06 | 0.02 | 3.64 |
| 165 | miR165 | GAAUGUUGUCUGGCUCGAGG | AUG | 20 | 124 | 54.84 | 0.84 | 0.35 | 0.14 | 0.05 | 3.23 |
| 166 | miR166a* | UCGGACCAGGCUUCAUUCCCC | UUC | 21 | 125 | 54.40 | 0.83 | 0.34 | 0.14 | 0.05 | 4.80 |
|  | miR166b* | GGAAUGUUGUCUGGCUCGAGG |  | 21 | 159 | 53.46 | 0.82 | 0.35 | 0.14 | 0.05 | 3.77 |
|  | miR166c* | UCGGACCAGGCUUCAUUCCC |  | 20 | 440 | 61.14 | 0.61 | 0.31 | 0.47 | 0.14 | 2.50 |
|  | miR166d | GAAUGUUGUCUUGUUCAAG |  | 19 | 133 | 61.65 | 0.47 | 0.29 | 0.37 | 0.13 | 6.02 |
| 167 | miR167a | GGUCAUGCUGUGACAGCCUCACU | UGA | 23 | 144 | 52.78 | 0.81 | 0.33 | 0.14 | 0.05 | 3.47 |
|  | miR167b | AGAUCAUGUGGCUGCUUCAC |  | 20 | 64 | 57.81 | 1.13 | 0.34 | 0.03 | 0.01 | 3.13 |
|  | miR167c | GAAGCUGCAGGAUGACCU |  | 18 | 164 | 50.00 | 0.83 | 0.37 | 0.20 | 0.08 | 3.66 |
|  | miR167d* | UGAAGCUGCCAGCAUGAUCUG |  | 21 | 105 | 55.24 | 1.11 | 0.35 | 0.05 | 0.02 | 3.81 |
|  | miR167e | GGUGGAGCUUCAACAUGAUC |  | 20 | 396 | 57.07 | 0.55 | 0.28 | 0.33 | 0.10 | 2.53 |
| 169 | miR169a | UGGGGAAGUCAUCCUUGGCU | UUG | 20 | 55 | 56.36 | 0.45 | 0.33 | 0.27 | 0.09 | 5.45 |
|  | miR169b | GCAGUCUCCUUGGAUA |  | 16 | 412 | 50.73 | 0.68 | 0.33 | 0.32 | 0.10 | 2.91 |
|  | miR169c | UGGCAAUCCAUCCUUGGCU |  | 19 | 425 | 59.53 | 0.72 | 0.33 | 0.46 | 0.13 | 3.29 |
|  | miR169d | UUAGCCAAGAAUGGAUG |  | 17 | 498 | 66.67 | 0.73 | 0.29 | 0.45 | 0.13 | 4.02 |
| 171 | miR171a | UUGAGCCGCGUCAAUAUCUC | UUG | 20 | 83 | 57.83 | 1.10 | 0.40 | 0.07 | 0.03 | 3.61 |
|  | miR171b | UGUUGGCAUGGAGCAAUCAAA |  | 21 | 215 | 56.28 | 0.67 | 0.32 | 0.29 | 0.09 | 4.65 |
|  | miR171c | GAUAUUGGCGCGGCUCAAUC |  | 20 | 447 | 57.05 | 0.69 | 0.28 | 0.46 | 0.15 | 3.13 |
|  | miR171d | AGGUAUUGGCGCGCCUCAAU |  | 20 | 97 | 56.70 | 1.00 | 0.38 | 0.06 | 0.03 | 4.12 |
|  | miR171e | UCGGAUUGAAGAACGCCAAU |  | 20 | 79 | 68.35 | 0.82 | 0.37 | 0.11 | 0.04 | 5.06 |
| 319 | miR319a | UGAAUGGAUGAUUUGGCAG | CUU | 19 | 228 | 57.89 | 0.62 | 0.35 | 0.38 | 0.12 | 2.63 |
|  | miR319b | GAGCUUGCUUGAGUCCAUU |  | 19 | 132 | 56.82 | 0.67 | 0.35 | 0.29 | 0.10 | 3.79 |
|  | miR319c* | UUGGACUGAAGGGAGCUCCUUC |  | 22 | 410 | 63.17 | 0.60 | 0.27 | 0.44 | 0.13 | 4.39 |
| 395 | miR395a | UGAAGCGUUUGGAGGAACUC | ACU | 20 | 110 | 49.09 | 0.68 | 0.37 | 0.04 | 0.02 | 2.73 |
| 396 | miR396a | UCCACGACCUUCUUGAAU | CUU | 18 | 383 | 55.61 | 0.62 | 0.30 | 0.28 | 0.09 | 2.87 |
|  | miR396b | GUUCAAGAAAGCCUGUGGA |  | 19 | 421 | 51.78 | 0.69 | 0.33 | 0.17 | 0.06 | 2.85 |
| 398 | miR398a | AGGGGUGACCUGAGAACACA | UCA | 20 | 106 | 61.32 | 0.79 | 0.29 | 0.11 | 0.03 | 3.77 |
| 399 | miR399a | UGCCAAAGGAGAGUUGCCC | UGC | 19 | 125 | 52.00 | 1.01 | 0.38 | 0.20 | 0.08 | 3.20 |
| 408 | miR408a | CAUGCACUGCCUCUUCCCUG | AGA | 20 | 113 | 51.33 | 0.81 | 0.33 | 0.08 | 0.03 | 6.19 |
| 419 | miR419a | UGAUGAAUGAUGAGGAUGU | AUG | 19 | 58 | 58.62 | 0.48 | 0.28 | 0.18 | 0.06 | 8.62 |
| 477 | miR477a | UCUUCUUCAAAGCCUUCU | CUU | 18 | 104 | 57.69 | 0.56 | 0.35 | 0.33 | 0.11 | 5.77 |
|  | miR477b | UGGAAGCCUUGUGGGAGA |  | 18 | 271 | 59.41 | 0.76 | 0.32 | 0.36 | 0.11 | 2.95 |
| 482 | miR482a | UUUCCUAUCCCUCCCAUUCC | UUC | 20 | 505 | 60.99 | 0.59 | 0.31 | 0.32 | 0.10 | 4.55 |
| 529 | miR529a | GGGUAGUGCUCUCUCCCUUCU | UCU | 21 | 252 | 63.89 | 0.70 | 0.31 | 0.29 | 0.10 | 3.17 |
|  | miR529b | GGGUUGUGCUCUCUCUCUUCU |  | 21 | 85 | 54.12 | 1.04 | 0.34 | 0.14 | 0.05 | 4.71 |
|  | miR529c | GGUUUUGCUCUCUGUCUUCU |  | 20 | 399 | 53.38 | 0.73 | 0.35 | 0.35 | 0.11 | 4.26 |
| 771 | miR771a | CAUGAAGAUAUGAGGAGCC | AUG | 19 | 326 | 60.43 | 0.68 | 0.33 | 0.25 | 0.07 | 4.29 |
| 773 | miR773a | CAACAGUUUUUUCAAAGAUA | UUG | 20 | 502 | 65.94 | 0.67 | 0.31 | 0.39 | 0.12 | 3.19 |
| 774 | miR774a | CUGAGAGCAAGAUUUUGGUG | AUU | 20 | 491 | 54.79 | 0.68 | 0.32 | 0.36 | 0.12 | 2.65 |
| 829 | miR829a | CCAUCAUUUGGUAUCAGAGCU | UGA | 21 | 469 | 67.80 | 0.66 | 0.28 | 0.47 | 0.14 | 2.99 |
|  | miR829b | GCUCUGAUACCAAAUGAUGGA |  | 21 | 88 | 60.23 | 0.66 | 0.34 | 0.28 | 0.12 | 5.68 |
| 831 | miR831a | AAGAGGUAAAGGAGAUGAG | AAG | 19 | 340 | 60.29 | 0.68 | 0.34 | 0.42 | 0.12 | 4.71 |
| 846 | miR846a | UUCACGGACUUCAAAUCAGA | UGA | 20 | 498 | 68.27 | 0.70 | 0.31 | 0.34 | 0.11 | 3.01 |
| 848 | miR848a | CAAUCCCAUGACAAA | CUU | 15 | 146 | 43.15 | 0.68 | 0.32 | 0.19 | 0.07 | 4.11 |
|  | miR848b | GGCAGUCCCAUGUCA |  | 15 | 314 | 48.09 | 0.72 | 0.34 | 0.24 | 0.08 | 3.18 |
|  | miR848c | UUUGACAAGGGAUUGC |  | 16 | 189 | 62.43 | 0.61 | 0.30 | 0.33 | 0.11 | 3.17 |
|  | miR848d | UUGACAUGGGUUUGC |  | 15 | 199 | 55.78 | 0.65 | 0.32 | 0.41 | 0.12 | 2.51 |
|  | miR848e | GCAAUCUCAUGUCAA |  | 15 | 142 | 66.20 | 0.79 | 0.30 | 0.27 | 0.09 | 4.23 |
|  | miR848f | UGACAUGGGAUUGC |  | 14 | 221 | 66.52 | 0.74 | 0.32 | 0.33 | 0.10 | 2.71 |
|  | miR848g | UUUGACAAGGGAUUG |  | 15 | 318 | 56.92 | 0.68 | 0.30 | 0.37 | 0.12 | 2.52 |
| 861 | miR861a | GAUGGAUAUGUCUUCUAGAA | AUG | 20 | 57 | 56.14 | 0.85 | 0.39 | 0.02 | 0.01 | 5.26 |
| 863 | miR863a | UUAUUUCUUGUUGAUCUCA | AUA | 19 | 497 | 72.23 | 0.75 | 0.31 | 0.44 | 0.13 | 3.02 |
| 866 | miR866a | AAGGAACGGAGUUUGUUAA | AUU | 19 | 114 | 65.79 | 0.80 | 0.35 | 0.16 | 0.06 | 2.63 |
|  | miR866b | AAAAUCCAUCUUUGAAGA |  | 18 | 126 | 71.43 | 0.74 | 0.33 | 0.13 | 0.05 | 3.17 |
| 902 | miR902a | UUAUGAUGUGAUUCUUCAU | GAU | 19 | 381 | 57.48 | 0.64 | 0.35 | 0.36 | 0.11 | 2.89 |
|  | miR902b | CUAUGUUUCAGAUCCUUCU |  | 19 | 112 | 58.04 | 0.79 | 0.33 | 0.27 | 0.09 | 3.57 |
|  | miR902c | GUUAUGAUGGAGUUCUUCG |  | 19 | 393 | 57.51 | 0.73 | 0.31 | 0.41 | 0.12 | 3.56 |
| 919 | miR919a | AUCUCGGUCGAGCAUCUCGAU |  | 21 | 395 | 38.23 | 0.68 | 0.32 | 0.33 | 0.10 | 2.53 |
| 1029 | miR1029a | GUAUGUUUGGUUGAGAGA | UUG | 18 | 159 | 64.15 | 0.74 | 0.33 | 0.12 | 0.04 | 4.40 |
| 1030 | miR1030a | GGUGCAGGUGCGGAUGCAG | UGC | 19 | 184 | 48.91 | 0.64 | 0.30 | 0.40 | 0.13 | 6.52 |
| 1043 | miR1043a | CCUUCAACUUCAUCCGUG | GAA | 18 | 93 | 53.76 | 0.64 | 0.33 | 0.09 | 0.03 | 4.30 |
| 1044 | miR1044a | UUGUAGUGUAUAUUUGUU | UUU | 18 | 494 | 70.45 | 0.86 | 0.33 | 0.37 | 0.11 | 5.06 |
| 1051 | miR1051a | GUUCAAGUAAAAAGGAAGA | GAA | 19 | 423 | 57.21 | 0.79 | 0.32 | 0.28 | 0.09 | 3.31 |
| 1052 | miR1052a | UUCCUUUGCUUGAUUGUGGU | UUG | 20 | 452 | 66.15 | 0.63 | 0.29 | 0.44 | 0.13 | 4.42 |
| 1075 | miR1075a | UGUUUCUGUCAUGAUUUCUAC | UUG | 21 | 437 | 60.64 | 0.55 | 0.26 | 0.47 | 0.14 | 2.97 |
| 1099 | miR1099a | UAUAGCAACGGUGUUUUUGUC | AAG | 21 | 382 | 52.88 | 0.64 | 0.29 | 0.32 | 0.11 | 3.66 |
| 1134 | miR1134a | UCUUCCUCUUCUUGUUGUUGUU | CAA | 22 | 251 | 64.94 | 0.71 | 0.25 | 0.28 | 0.09 | 3.19 |
| 1217 | miR1217a | AUUUGAAGCCUGAUAUCAAG | UUG | 20 | 378 | 57.41 | 0.71 | 0.31 | 0.37 | 0.11 | 2.91 |
| 1428 | miR1428a | CGUUUUGAAAAUUCACAGGC | AUU | 20 | 353 | 66.57 | 0.53 | 0.26 | 0.33 | 0.10 | 4.53 |
| 1441 | miR1441a | CCGGAUGUGGGCAAAGGUUU | UUU | 20 | 242 | 49.59 | 0.75 | 0.34 | 0.19 | 0.06 | 4.96 |
| 1514 | miR1514a | AUGUCCAUUUGAAAAAUGAA | UUU | 20 | 281 | 65.48 | 0.72 | 0.33 | 0.29 | 0.09 | 4.27 |
|  | miR1514b | UUCAUUUUGAAAAUAGGCAUU |  | 21 | 80 | 68.75 | 1.12 | 0.36 | 0.14 | 0.05 | 5.00 |
| 1519 | miR1519a | AGUGUUGCAAGAUAGUCAUU | UUU | 20 | 65 | 67.69 | 0.70 | 0.31 | 0.03 | 0.01 | 3.08 |
| 1527 | miR1527a | GGUUUUAUAAGGUUGAGUU | AAC | 19 | 203 | 65.02 | 0.70 | 0.34 | 0.29 | 0.09 | 2.96 |
|  | miR1527b | UAACUCAACCUUACAAAA |  | 18 | 360 | 76.94 | 0.78 | 0.31 | 0.20 | 0.06 | 2.50 |
|  | miR1527c | UAACUCAACCUUAUAAAACC |  | 20 | 323 | 68.11 | 0.75 | 0.33 | 0.43 | 0.14 | 3.41 |
|  | miR1527d | UAACUCAACCUCAAAAAACC |  | 20 | 489 | 65.03 | 0.65 | 0.27 | 0.39 | 0.12 | 2.86 |
|  | miR1527e | UAACUCAACCUCACAAAAC |  | 19 | 409 | 66.26 | 0.63 | 0.31 | 0.44 | 0.14 | 3.67 |
|  | miR1527f | UUUUGUAAGGUUAAGUUA |  | 18 | 132 | 72.73 | 0.58 | 0.33 | 0.34 | 0.11 | 3.03 |
|  | miR1527g | UAACUCAAUCUUACAAAAC |  | 19 | 255 | 69.41 | 0.83 | 0.33 | 0.18 | 0.06 | 2.75 |
|  | miR1527h | AACUCAACCUUACAAAA |  | 17 | 55 | 70.91 | 0.67 | 0.31 | 0.31 | 0.13 | 5.45 |
|  | miR1527i | UAACUCAACCUCAUAAAACC |  | 20 | 164 | 54.88 | 1.19 | 0.41 | 0.09 | 0.04 | 3.66 |
|  | miR1527j | UAACUCAACCUUAUAAAAC |  | 19 | 55 | 72.73 | 1.07 | 0.29 | 0.05 | 0.02 | 7.27 |
| 1533 | miR1533a | UAAUAAAAAUAAUAAU | UAU | 16 | 291 | 76.63 | 0.80 | 0.34 | 0.48 | 0.14 | 3.78 |
|  | miR1533b | AUAAUAAUAAUAAUAAU |  | 17 | 483 | 73.29 | 0.80 | 0.33 | 0.34 | 0.11 | 2.69 |
|  | miR1533c | AUUAUUAUUUUUAUUAU |  | 17 | 142 | 76.76 | 0.74 | 0.33 | 0.24 | 0.08 | 8.45 |
|  | miR1533d | UUAUUAUUUUUAUUAU |  | 16 | 438 | 76.94 | 0.71 | 0.29 | 0.34 | 0.11 | 6.62 |
|  | miR1533e | AUAAUAAUAAUAAUGAUGA |  | 19 | 210 | 69.52 | 0.79 | 0.34 | 0.46 | 0.14 | 5.24 |
|  | miR1533f | AUAAUAAAAAUAAUAAU |  | 17 | 474 | 74.68 | 0.82 | 0.33 | 0.28 | 0.09 | 3.80 |
|  | miR1533g | UCAUUAUUAAUUUUAUUA |  | 18 | 101 | 74.26 | 0.71 | 0.34 | 0.07 | 0.02 | 8.91 |
|  | miR1533h | AUUAUCAUUUUUAUUAU |  | 17 | 505 | 76.24 | 0.72 | 0.30 | 0.30 | 0.09 | 4.55 |
|  | miR1533i | AUAAUAAAAAUAAUAA |  | 16 | 499 | 73.55 | 0.60 | 0.26 | 0.39 | 0.12 | 3.21 |
|  | miR1533j | AUUAUUAUUUUUAUUA |  | 16 | 203 | 76.35 | 1.06 | 0.34 | 0.24 | 0.09 | 7.88 |
|  | miR1533k | AUUAAUAUUUUUAUUAU |  | 17 | 352 | 75.00 | 0.85 | 0.31 | 0.24 | 0.07 | 6.25 |
|  | miR1533l | AUUAUUAUUAUUAUUAU |  | 17 | 502 | 69.72 | 0.73 | 0.33 | 0.30 | 0.10 | 4.98 |
|  | miR1533m | UCAUUAUUUUUUAUAUUAU |  | 19 | 498 | 76.31 | 1.07 | 0.33 | 0.28 | 0.09 | 6.22 |
|  | miR1533n | CAUUAUUAUUAUUAUUAU |  | 18 | 395 | 60.51 | 0.58 | 0.32 | 0.29 | 0.09 | 3.29 |
|  | miR1533o | UCAUUAUUUUUAUUAUUAU |  | 19 | 391 | 65.47 | 0.69 | 0.31 | 0.33 | 0.11 | 3.07 |
| 1846 | miR1846a | UGGUCCCGGCCUUCUCGU | CCG | 18 | 125 | 44.00 | 0.56 | 0.27 | 0.41 | 0.14 | 4.80 |
| 1860 | miR1860a | AGAUCUGUAGGCUGGUUUUC | UUU | 20 | 338 | 59.47 | 0.75 | 0.32 | 0.22 | 0.08 | 2.66 |
| 1888 | miR1888a | AAGUUAAGAAUUGAGAAGAA | UUA | 20 | 113 | 70.80 | 1.00 | 0.35 | 0.13 | 0.05 | 2.65 |
| 1916 | miR1916a | UGAGAUGUCUAAGUGAAA | UGA | 18 | 86 | 76.74 | 0.68 | 0.35 | 0.18 | 0.07 | 3.49 |
| 2082 | miR2082a | UGUGUGUUCUUCUUCUUCUU | AGA | 20 | 98 | 59.18 | 0.76 | 0.32 | 0.31 | 0.11 | 4.08 |
| 2088 | miR2088a | UAGACCUGGAUUUCAUUGGA | UCU | 20 | 287 | 62.37 | 0.73 | 0.32 | 0.28 | 0.09 | 3.48 |
| 2095 | miR2095a | CUUCCAUGAAUGAUAAGUAU | AUA | 20 | 471 | 60.51 | 0.60 | 0.27 | 0.35 | 0.10 | 2.76 |
| 2105 | miR2105a | UUGUGUUGUGUAUGAUUCAU | AUU | 20 | 256 | 53.91 | 0.66 | 0.34 | 0.20 | 0.06 | 2.73 |
| 2109 | miR2109a | UCAGAGGCGAUGACACUCG | GAG | 19 | 193 | 55.44 | 0.74 | 0.31 | 0.37 | 0.12 | 2.59 |
| 2606 | miR2606a | AAAAACACCUAAGGAAUUG | AAU | 19 | 256 | 70.31 | 0.77 | 0.37 | 0.25 | 0.10 | 3.52 |
|  | miR2606b | ACAAUGCCUUAUGUGCUUUU |  | 20 | 263 | 62.74 | 0.74 | 0.35 | 0.24 | 0.09 | 2.66 |
| 2610 | miR2610a | AGAUUGAGAUUUCUAUGGCU | UUG | 20 | 121 | 71.90 | 0.54 | 0.33 | 0.43 | 0.14 | 4.96 |
| 2673 | miR2673a | UCUUCCUCUUCCUCUCCCAC | UUG | 20 | 342 | 52.34 | 0.62 | 0.32 | 0.36 | 0.11 | 2.92 |
|  | miR2673b | GAAGAGGAAGAGGAAGAGG |  | 19 | 217 | 58.53 | 0.71 | 0.35 | 0.47 | 0.15 | 2.76 |
| 2873 | miR2873a | CAAAUGAAGCUGUGUUUGGA | UUU | 20 | 204 | 68.63 | 0.86 | 0.35 | 0.16 | 0.05 | 6.37 |
| 2934 | miR2934a | CCAAGGAGUUUUCAGAAAGA | UUU | 20 | 204 | 69.61 | 1.07 | 0.36 | 0.13 | 0.04 | 5.88 |
| 2938 | miR2938a | GAUCUUUUCAAAGGGUUCCAG | UUU | 21 | 86 | 60.47 | 0.80 | 0.29 | 0.15 | 0.06 | 6.98 |
| 3440 | miR3440a | GGAUUGGUAAGAGAACCG | UUG | 18 | 354 | 57.06 | 0.62 | 0.31 | 0.45 | 0.14 | 2.54 |
|  | miR3440b | GCUUCCCUUGAACAAUCCA |  | 19 | 103 | 48.54 | 0.71 | 0.34 | 0.09 | 0.04 | 3.88 |
|  | miR3440c | UGGAUUGUUCAAGGGAAGC |  | 19 | 100 | 48.00 | 0.84 | 0.38 | 0.08 | 0.03 | 4.00 |
| 3442 | miR3442a | ACCAUGUUUAACUCUGAA | UUU | 18 | 484 | 64.67 | 0.70 | 0.30 | 0.27 | 0.09 | 2.89 |
|  | miR3442b | CCAUGUUUUGAAUCUGAAUU |  | 20 | 481 | 68.61 | 0.97 | 0.37 | 0.16 | 0.06 | 3.95 |
| 3444 | miR3444a | UUGGGAGCUCUGAUGAGAUC | AUC | 20 | 140 | 52.14 | 0.64 | 0.31 | 0.25 | 0.09 | 4.29 |
| 3630 | miR3630a | UGGGAAUCUCUUUGAUGCUU | UGA | 20 | 500 | 55.20 | 0.71 | 0.32 | 0.35 | 0.12 | 4.00 |
| 3633 | miR3633a | UCUCCUACCAAUCCAUUCC | UUC | 19 | 249 | 56.22 | 0.69 | 0.32 | 0.36 | 0.12 | 2.81 |
| 3711 | miR3711a | GGCCCUCCUUCUAACGCC | ACC | 18 | 411 | 40.15 | 0.56 | 0.28 | 0.33 | 0.11 | 2.92 |
| 3954 | miR3954a | UGACCUUGAUUUCUCUGUC | UUC | 19 | 299 | 65.89 | 0.84 | 0.35 | 0.25 | 0.09 | 3.01 |
| 3979 | miR3979a | UUCAAGGGAGAGAGAGA | AAG | 17 | 85 | 67.06 | 0.81 | 0.33 | 0.10 | 0.03 | 3.53 |
| 4224 | miR4224a | AGAGAUGAGGAGGCUGAGUUG | AAA | 21 | 438 | 51.37 | 0.71 | 0.34 | 0.39 | 0.13 | 2.51 |
| 4225 | miR4225a | UUGUUUAAGCCAUCGAUUC | AUU | 19 | 387 | 66.93 | 0.68 | 0.30 | 0.34 | 0.10 | 3.36 |
| 4243 | miR4243a | UGAAAUUAUGGAAUUCGUAC | AUU | 20 | 333 | 73.27 | 0.59 | 0.25 | 0.36 | 0.11 | 4.50 |
| 4245 | miR4245a | AUUGUCAGAAUAAUACUUUG | AUU | 20 | 402 | 68.91 | 0.70 | 0.31 | 0.47 | 0.14 | 3.23 |
| 4246 | miR4246a | UAAGUAAUGAACAUUGGAUUU | UUU | 21 | 179 | 70.39 | 0.65 | 0.33 | 0.28 | 0.10 | 3.35 |
| 4345 | miR4345a | AAUCUUUGUAAGAUCAGUCUU | CUU | 21 | 193 | 55.44 | 0.70 | 0.36 | 0.33 | 0.10 | 2.59 |
|  | miR4345b | AAGACUGAUCUUACAAAGAUU |  | 21 | 284 | 58.45 | 0.64 | 0.32 | 0.26 | 0.08 | 3.17 |
| 4413 | miR4413a | GUGACUUACACUGCUCUUA | AUU | 19 | 179 | 59.22 | 0.64 | 0.32 | 0.41 | 0.13 | 3.35 |
| 5014 | miR5014a | AUGUUGUACAAGACUAAGU | ACA | 19 | 279 | 65.23 | 0.53 | 0.28 | 0.47 | 0.14 | 3.58 |
| 5021 | miR5021a | GAGAAGAAGAAGAAGAA | AGA | 17 | 254 | 48.43 | 0.61 | 0.27 | 0.46 | 0.14 | 3.54 |
|  | miR5021b | UUUCUUCUUCUUCUUCU |  | 17 | 145 | 61.38 | 0.72 | 0.33 | 0.38 | 0.12 | 4.83 |
|  | miR5021c | AGAAGAAGAAGAAGAAA |  | 17 | 123 | 52.03 | 0.66 | 0.33 | 0.26 | 0.09 | 5.69 |
|  | miR5021d | UUCUUCUUCUUCUUCUCA |  | 18 | 87 | 63.22 | 0.71 | 0.30 | 0.27 | 0.11 | 3.45 |
|  | miR5021e | GAGAAGAAGAAGAAGAAA |  | 18 | 296 | 52.03 | 0.65 | 0.33 | 0.34 | 0.11 | 7.09 |
|  | miR5021f | GAAGAAGAAGAAGAAAA |  | 17 | 114 | 64.91 | 0.84 | 0.39 | 0.17 | 0.06 | 7.89 |
|  | miR5021g | AGAAGAAGAAGAAGAAAA |  | 18 | 296 | 56.08 | 0.78 | 0.36 | 0.42 | 0.13 | 4.73 |
|  | miR5021h | UUCUUCUUCUUCUUCUC |  | 17 | 405 | 53.09 | 0.76 | 0.34 | 0.20 | 0.07 | 3.70 |
|  | miR5021i | UUUUCUUCUUCUUCUUC |  | 17 | 247 | 70.45 | 0.71 | 0.31 | 0.41 | 0.13 | 3.24 |
|  | miR5021j | UUUUCUUCUUCUUCUUCU |  | 18 | 147 | 53.06 | 0.70 | 0.31 | 0.16 | 0.05 | 4.08 |
| 5041 | miR5041a | UUGAGCAAGUUGAAGAUG | UUG | 18 | 136 | 60.29 | 0.69 | 0.34 | 0.35 | 0.11 | 5.15 |
| 5054 | miR5054a | CCCCACGGUGGGCGCCA | GAU | 17 | 57 | 43.86 | 0.62 | 0.35 | 0.06 | 0.02 | 3.51 |
|  | miR5054b | UGGCGCCCACCGUGGGG |  | 17 | 393 | 46.06 | 0.65 | 0.31 | 0.27 | 0.09 | 2.54 |
|  | miR5054c | CCCCACGGUGGGCGCC |  | 16 | 95 | 42.11 | 0.42 | 0.27 | 0.26 | 0.08 | 3.16 |
| 5057 | miR5057a | UGUCAAAAUGAGUUGAAAU | AAA | 19 | 364 | 75.27 | 0.72 | 0.32 | 0.27 | 0.09 | 3.30 |
| 5083 | miR5083a | AGACUACAAUUAUCUGAUC | UUC | 19 | 441 | 69.84 | 0.69 | 0.31 | 0.32 | 0.10 | 2.72 |
| 5140 | miR5140a | GCUGGUGAAGAUUUGG | UGG | 16 | 476 | 57.56 | 0.74 | 0.32 | 0.24 | 0.08 | 3.15 |
| 5169 | miR5169a | UUGACCUAGUUUAUAGAACA | UUG | 20 | 145 | 62.76 | 0.69 | 0.35 | 0.34 | 0.12 | 2.76 |
| 5176 | miR5176a | UAUGCCAUGUUGUCCCAUAU | AUG | 20 | 75 | 70.67 | 0.79 | 0.35 | 0.09 | 0.04 | 5.33 |
| 5177 | miR5177a | CUGUUUGUUUUACACCCUCA | GUU | 20 | 89 | 52.81 | 0.50 | 0.31 | 0.14 | 0.05 | 3.37 |
| 5179 | miR5179a | UUUUGCACAAGACCGAGCAAC | UUG | 21 | 483 | 64.80 | 0.67 | 0.30 | 0.30 | 0.10 | 2.69 |
| 5213 | miR5213a | UUCAGAGGGGAAGACACACGU | UUC | 21 | 103 | 55.34 | 0.66 | 0.33 | 0.11 | 0.04 | 3.88 |
| 5248 | miR5248a | AUGCAUGCCAACUAAAAA | UUG | 18 | 131 | 67.18 | 0.53 | 0.31 | 0.47 | 0.14 | 3.82 |
| 5255 | miR5255a | ACUUGAUAGAGGAAAUGGG | AUG | 19 | 469 | 58.64 | 0.69 | 0.34 | 0.38 | 0.12 | 2.56 |
| 5261 | miR5261a | GCCAAAGCCAUCUACAAU | AAU | 18 | 226 | 63.27 | 0.64 | 0.30 | 0.40 | 0.13 | 3.54 |
|  | miR5261b | AGCCAAAGCAAUCUUCAAUGA |  | 21 | 59 | 66.10 | 0.60 | 0.32 | 0.20 | 0.08 | 3.39 |
| 5264 | miR5264a | UUGAUCAAGGCCUUGGCAU | UUG | 19 | 446 | 56.95 | 0.66 | 0.31 | 0.35 | 0.11 | 2.91 |
| 5281 | miR5281a | UCUUAUAAUAAGGACCGGAGGGAG | AAU | 24 | 301 | 68.44 | 0.77 | 0.31 | 0.37 | 0.13 | 3.32 |
| 5298 | miR5298a | UGAUGGAGAUGAUCAUGAAGAU | GAU | 22 | 230 | 61.74 | 0.70 | 0.34 | 0.17 | 0.06 | 6.52 |
| 5368 | miR5368a* | GGACAGUCUCAGGUAGACA | AGA | 19 | 171 | 44.44 | 0.77 | 0.35 | 0.32 | 0.11 | 3.51 |
|  | miR5368b | UGUCUACCUGAGACUGUCC |  | 19 | 223 | 46.64 | 0.53 | 0.30 | 0.46 | 0.14 | 2.69 |
| 5555 | miR5555a | UCUAAGAGUGGAAUAUGACU | UAU | 20 | 433 | 67.21 | 0.79 | 0.34 | 0.37 | 0.12 | 2.77 |
| 5558 | miR5558a | UUUUCUAAUUCUAAUUCUA | AUU | 19 | 283 | 57.95 | 0.58 | 0.30 | 0.17 | 0.06 | 4.59 |
|  | miR5558b | GAUAAACUUACAAUUGGAAAA |  | 21 | 204 | 68.63 | 0.77 | 0.31 | 0.46 | 0.15 | 3.43 |
| 5562 | miR5562a | GUGGAGAAGCCUGCAAC | CAA | 17 | 96 | 53.13 | 0.79 | 0.35 | 0.07 | 0.03 | 4.17 |
| 5654 | miR5654a | AAAUCCCAACAGCUUCCA | CUU | 18 | 116 | 55.17 | 0.56 | 0.28 | 0.25 | 0.09 | 5.17 |
|  | miR5654b | UGGAAGAGGUUGGGAUUU |  | 18 | 434 | 53.69 | 0.64 | 0.29 | 0.24 | 0.08 | 3.23 |
| 5662 | miR5662a | GAGGUGACCAUUGCAGAUG | AAU | 19 | 111 | 56.76 | 0.57 | 0.33 | 0.38 | 0.12 | 2.70 |
| 5674 | miR5674a | GAUAAUGGAUAACACAAUUA | CAA | 20 | 408 | 69.12 | 0.65 | 0.28 | 0.39 | 0.12 | 2.94 |
| 5675 | miR5675a | UAGAGACAACAACAAGGGAA | AAU | 20 | 355 | 62.25 | 0.78 | 0.34 | 0.21 | 0.07 | 2.82 |
| 5721 | miR5721a | AAAAUGGAAUGACAAAUGGA | UUU | 20 | 497 | 64.59 | 0.72 | 0.28 | 0.34 | 0.09 | 2.62 |
|  | miR5721b | AAAAAUGGAGUGAGAAAU |  | 18 | 155 | 67.74 | 0.76 | 0.34 | 0.27 | 0.09 | 3.23 |
|  | miR5721c | UCCAUUUGUCAUUCCAUUUU |  | 20 | 328 | 67.38 | 0.52 | 0.27 | 0.28 | 0.08 | 3.05 |
| 5741 | miR5741a | UAGGGACCAAAUUAAUGGUU | AUU | 20 | 231 | 69.70 | 0.58 | 0.30 | 0.45 | 0.14 | 3.03 |
| 5773 | miR5773a | UUUUAUAAGGUUGAGUUAGGU | AAA | 21 | 471 | 70.49 | 0.80 | 0.32 | 0.24 | 0.09 | 2.76 |
| 5778 | miR5778a | CGACGACCUCUUCGUCGGCAUC | UCG | 22 | 113 | 47.79 | 0.97 | 0.39 | 0.03 | 0.01 | 4.42 |
| 5820 | miR5820a | UGGCUGAGAUUGAUGGAGGAA | AUU | 21 | 237 | 55.27 | 0.67 | 0.32 | 0.34 | 0.11 | 3.38 |
| 5998 | miR5998a | AGUUUGGGUUUUGUUUUGU | UUG | 19 | 233 | 56.65 | 0.56 | 0.32 | 0.44 | 0.13 | 6.44 |
|  | miR5998b | AGUUUUUGUUUUGUUUUGU |  | 19 | 134 | 73.13 | 0.73 | 0.35 | 0.42 | 0.15 | 5.22 |
| 6027 | miR6027a | UUAUGGAUAGCAGAAGGAUU | GAA | 20 | 171 | 59.65 | 0.53 | 0.29 | 0.30 | 0.08 | 5.26 |
| 6034 | miR6034a | UGAUGUUUAUAGCUUUGGG | UAU | 19 | 439 | 63.55 | 0.66 | 0.31 | 0.39 | 0.12 | 2.96 |
|  | miR6034b | CUGAUGUCUAUAGCUUUGG |  | 19 | 75 | 56.00 | 0.61 | 0.32 | 0.02 | 0.01 | 4.00 |
|  | miR6034c | UCUGAUGUCUAUAGUUUUGG |  | 20 | 150 | 62.00 | 0.70 | 0.31 | 0.23 | 0.08 | 4.67 |
|  | miR6034d | UGAUGUAUAUAGUUUUGGG |  | 19 | 132 | 60.61 | 0.77 | 0.31 | 0.19 | 0.07 | 3.79 |
| 6114 | miR6114a | UGAAAGUGACCAUGGACGUG | UUU | 20 | 400 | 71.50 | 0.63 | 0.31 | 0.38 | 0.12 | 5.75 |
| 6167 | miR6167a | UACCCAGGUGGAAGCUUU | ACC | 18 | 80 | 50.00 | 0.65 | 0.33 | 0.17 | 0.06 | 3.75 |
| 6169 | miR6169a | GUAUUUCUCUUUUUUCUCU | AAU | 19 | 263 | 68.82 | 0.67 | 0.27 | 0.14 | 0.04 | 4.56 |
|  | miR6169b | AGAGAGAAAAGAGAAAUA |  | 18 | 360 | 61.94 | 0.75 | 0.31 | 0.41 | 0.13 | 3.33 |
| 6171 | miR6171a | UGUGGAUUGCUUGAAGGCUU | AUU | 20 | 387 | 70.80 | 0.57 | 0.28 | 0.45 | 0.13 | 4.39 |
| 6196 | miR6196a | ACGAGGAGAUGGAGAGGA | GAG | 18 | 324 | 52.16 | 0.67 | 0.28 | 0.21 | 0.07 | 5.56 |
| 6214 | miR6214a | CGACGACGACGAGCACGA | CGG | 18 | 168 | 33.33 | 0.62 | 0.31 | 0.11 | 0.04 | 2.98 |
| 6470 | miR6470a | UUUUCUAAUAUGGUAUCAGAG | UUG | 21 | 245 | 71.84 | 0.69 | 0.33 | 0.32 | 0.10 | 3.67 |
|  | miR6470b | UUUUUUAAUAUGGUAUCAGAG |  | 21 | 416 | 74.52 | 0.75 | 0.33 | 0.45 | 0.14 | 2.64 |
|  | miR6470c | UCUGAAAUCAUAUUAAAAA |  | 19 | 363 | 65.56 | 0.71 | 0.31 | 0.26 | 0.09 | 4.96 |
| 6479 | miR6479a | GCAGUAGUAUUCUCAUUG | CAA | 18 | 318 | 71.70 | 0.79 | 0.33 | 0.36 | 0.12 | 2.83 |
| 6484 | miR6484a | UAAUGGGUUCUGCACAGAUG | AAU | 20 | 279 | 61.29 | 0.54 | 0.29 | 0.26 | 0.08 | 3.94 |

^a^Conserved SSR signature sequence of the miRNA family.
^b^Length of mature miRNA sequence.
^c^Length of precursor miRNA.
^d^Percentage AU content of precursor miRNA.
^e^Minimum Folding Energy Index of precursor miRNA.
^f^Normalized base-pairing propensity of precursor miRNA.
^g^Normalised Shannon entropy of precursor miRNA.
^h^Normalized base-pair distance of precursor miRNA.
^i^SSR signature repeats per 100 nucleotides of precursor miRNA.
*Non-novel miRNA
