# Supplementary material for: Computational prediction of miRNAs and their targets in Phaseolus vulgaris using simple sequence repeat signatures
Source: BMC Plant Biol. 2015 Jun 12;15:140. doi: 10.1186/s12870-015-0516-3 (PMC4464996; doi:10.1186/s12870-015-0516-3)
Supplement: Supplementary file 3 — P. vulgaris miRNAs obtained from both computational prediction and small RNA sequencing. [file 12870_2015_516_MOESM3_ESM.docx]

# **Table S3: *P. vulgaris* miRNAs obtained from both computational prediction and small RNA sequencing**

| miRNA | Family | miRNA sequence | miRNA Length | Reads | TPM^a^ |
| --- | --- | --- | --- | --- | --- |
| pvu-miR166c | 166 | UCGGACCAGGCUUCAUUCCC | 20 | 37259 | 1108.81 |
| pvu-miR166a | 166 | UCGGACCAGGCUUCAUUCCCC | 21 | 36085 | 1073.87 |
| pvu-miR5054c | 5054 | CCCCACGGUGGGCGCC | 16 | 5613 | 167.04 |
| pvu-miR1514b | 1514 | UUCAUUUUGAAAAUAGGCAUU | 21 | 2504 | 74.5179 |
| pvu-miR165a | 165 | GAAUGUUGUCUGGCUCGAGG | 20 | 1715 | 51.0376 |
| pvu-miR166b | 166 | GGAAUGUUGUCUGGCUCGAGG | 21 | 1659 | 49.3711 |
| pvu-miR5054a | 5054 | CCCCACGGUGGGCGCCA | 17 | 1548 | 46.0678 |
| pvu-miR5054b | 5054 | UGGCGCCCACCGUGGGG | 17 | 1548 | 46.0678 |
| pvu-miR159a | 159 | UUGGACUGAAGGGAGCUCCU | 20 | 1350 | 40.1754 |
| pvu-miR5368a | 5368 | GGACAGUCUCAGGUAGACA | 19 | 1290 | 38.3898 |
| pvu-miR5368b | 5368 | UGUCUACCUGAGACUGUCC | 19 | 1290 | 38.3898 |
| pvu-miR171c | 171 | GAUAUUGGCGCGGCUCAAUC | 20 | 777 | 23.1232 |
| pvu-miR167b | 167 | AGAUCAUGUGGCUGCUUCAC | 20 | 757 | 22.528 |
| pvu-miR167d | 167 | UGAAGCUGCCAGCAUGAUCUG | 21 | 694 | 20.6531 |
| pvu-miR3711a | 3711 | GGCCCUCCUUCUAACGCC | 18 | 667 | 19.8496 |
| pvu-miR398a | 398 | AGGGGUGACCUGAGAACACA | 20 | 528 | 15.713 |
| pvu-miR1527j | 1527 | UAACUCAACCUUAUAAAAC | 19 | 497 | 14.7905 |
| pvu-miR5083 | 5083 | AGACUACAAUUAUCUGAUC | 19 | 403 | 11.9931 |
| pvu-miR1527a | 1527 | GGUUUUAUAAGGUUGAGUU | 19 | 364 | 10.8325 |
| pvu-miR1527e | 1527 | UAACUCAACCUCACAAAAC | 19 | 349 | 10.3861 |
| pvu-miR1527h | 1527 | AACUCAACCUUACAAAA | 17 | 307 | 9.13618 |
| pvu-miR1527b | 1527 | UAACUCAACCUUACAAAA | 18 | 294 | 8.74931 |
| pvu-miR171a | 171 | UUGAGCCGCGUCAAUAUCUC | 20 | 274 | 8.15412 |
| pvu-miR167c | 167 | GAAGCUGCAGGAUGACCU | 18 | 265 | 7.88628 |
| pvu-miR1527c | 1527 | UAACUCAACCUUAUAAAACC | 20 | 258 | 7.67797 |
| pvu-miR1527i | 1527 | UAACUCAACCUCAUAAAACC | 20 | 220 | 6.5471 |
| pvu-miR1527d | 1527 | UAACUCAACCUCAAAAAACC | 20 | 151 | 4.49369 |
| pvu-miR395a | 395 | UGAAGCGUUUGGAGGAACUC | 19 | 139 | 4.13658 |
| pvu-miR167a | 167 | GGUCAUGCUGUGACAGCCUCACU | 23 | 127 | 3.77946 |
| pvu-miR1527g | 1527 | UAACUCAAUCUUACAAAAC | 19 | 110 | 3.27355 |
| pvu-miR5021a | 5021 | GAGAAGAAGAAGAAGAA | 17 | 94 | 2.7974 |
| pvu-miR5021h | 5021 | UUCUUCUUCUUCUUCUC | 17 | 94 | 2.7974 |
| pvu-miR6470b | 6470 | UUUUUUAAUAUGGUAUCAGAG | 21 | 79 | 2.35101 |
| pvu-miR5773a | 5773 | UUUUAUAAGGUUGAGUUAGGU | 21 | 69 | 2.05341 |
| pvu-miR6470a | 6470 | UUUUCUAAUAUGGUAUCAGAG | 21 | 68 | 2.02365 |
| pvu-miR6470c | 6470 | UCUGAAAUCAUAUUAAAAA | 19 | 58 | 1.72605 |
| pvu-miR5021f | 5021 | GAAGAAGAAGAAGAAAA | 17 | 49 | 1.45822 |
| pvu-miR5021i | 5021 | UUUUCUUCUUCUUCUUC | 17 | 49 | 1.45822 |
| pvu-miR1527f | 1527 | UUUUGUAAGGUUAAGUUA | 18 | 46 | 1.36894 |
| pvu-miR5264a | 5264 | UUGAUCAAGGCCUUGGCAU | 19 | 41 | 1.22014 |
| pvu-miR5021c | 5021 | AGAAGAAGAAGAAGAAA | 17 | 37 | 1.1011 |
| pvu-miR5021b | 5021 | UUUCUUCUUCUUCUUCU | 17 | 36 | 1.07134 |
| pvu-miR399a | 399 | UGCCAAAGGAGAGUUGCCC | 19 | 34 | 1.01182 |
| pvu-miR1533i | 1533 | AUAAUAAAAAUAAUAA | 16 | 33 | 0.982065 |
| pvu-miR2673b | 2673 | GAAGAGGAAGAGGAAGAGG | 19 | 31 | 0.922546 |
| pvu-miR1533d | 1533 | UUAUUAUUUUUAUUAU | 16 | 28 | 0.833268 |
| pvu-miR1533f | 1533 | AUAAUAAAAAUAAUAAU | 17 | 27 | 0.803508 |
| pvu-miR2610a | 2610 | AGAUUGAGAUUUCUAUGGCU | 20 | 27 | 0.803508 |
| pvu-miR1533c | 1533 | AUUAUUAUUUUUAUUAU | 17 | 22 | 0.65471 |
| pvu-miR1533a | 1533 | UAAUAAAAAUAAUAAU | 16 | 21 | 0.624951 |
| pvu-miR1533j | 1533 | AUUAUUAUUUUUAUUA | 16 | 21 | 0.624951 |
| pvu-miR5021g | 5021 | AGAAGAAGAAGAAGAAAA | 18 | 21 | 0.624951 |
| pvu-miR5021j | 5021 | UUUUCUUCUUCUUCUUCU | 18 | 20 | 0.595191 |
| pvu-miR829b | 829 | GCUCUGAUACCAAAUGAUGGA | 21 | 18 | 0.535672 |
| pvu-miR1533k | 1533 | AUUAAUAUUUUUAUUAU | 17 | 14 | 0.416634 |
| pvu-miR319c | 319 | UUGGACUGAAGGGAGCUCCUUC | 22 | 14 | 0.416634 |
| pvu-miR5558a | 5558 | UUUUCUAAUUCUAAUUCUA | 19 | 13 | 0.386874 |
| pvu-miR829a | 829 | CCAUCAUUUGGUAUCAGAGCU | 21 | 12 | 0.357115 |
| pvu-miR5021d | 5021 | UUCUUCUUCUUCUUCUCA | 18 | 10 | 0.297596 |
| pvu-miR5021e | 5021 | GAGAAGAAGAAGAAGAAA | 18 | 10 | 0.297596 |
| pvu-miR848a | 848 | CAAUCCCAUGACAAA | 15 | 8 | 0.238076 |
| pvu-miR5721a | 5721 | AAAAUGGAAUGACAAAUGGA | 20 | 7 | 0.208317 |
| pvu-miR5721c | 5721 | UCCAUUUGUCAUUCCAUUUU | 20 | 7 | 0.208317 |
| pvu-miR3979 | 3979 | UUCAAGGGAGAGAGAGA | 17 | 6 | 0.178557 |
| pvu-miR477a | 477 | UCUUCUUCAAAGCCUUCU | 18 | 6 | 0.178557 |
| pvu-miR1533o | 1533 | UCAUUAUUUUUAUUAUUAU | 19 | 5 | 0.148798 |
| pvu-miR4225 | 4225 | UUGUUUAAGCCAUCGAUUC | 19 | 5 | 0.148798 |
| pvu-miR4345a | 4345 | AAUCUUUGUAAGAUCAGUCUU | 21 | 4 | 0.119038 |
| pvu-miR4345b | 4345 | AAGACUGAUCUUACAAAGAUU | 21 | 4 | 0.119038 |
| pvu-miR1533h | 1533 | AUUAUCAUUUUUAUUAU | 17 | 3 | 0.0892787 |
| pvu-miR5261a | 5261 | GCCAAAGCCAUCUACAAU | 18 | 3 | 0.0892787 |
| pvu-miR6034c | 6034 | UCUGAUGUCUAUAGUUUUGG | 20 | 3 | 0.0892787 |
| pvu-miR1519a | 1519 | AGUGUUGCAAGAUAGUCAUU | 20 | 2 | 0.0595191 |
| pvu-miR1533e | 1533 | AUAAUAAUAAUAAUGAUGA | 19 | 2 | 0.0595191 |
| pvu-miR156b | 156 | GUGCUCUCUCUCUUCUGUCAAC | 22 | 2 | 0.0595191 |
| pvu-miR1888a | 1888 | AAGUUAAGAAUUGAGAAGAA | 20 | 2 | 0.0595191 |
| pvu-miR408a | 408 | CAUGCACUGCCUCUUCCCUG | 20 | 2 | 0.0595191 |
| pvu-miR6034b | 6034 | CUGAUGUCUAUAGCUUUGG | 19 | 2 | 0.0595191 |
| pvu-miR773a | 773 | CAACAGUUUUUUCAAAGAUA | 20 | 2 | 0.0595191 |
| pvu-miR1052a | 1052 | UUCCUUUGCUUGAUUGUGGU | 20 | 1 | 0.0297596 |
| pvu-miR1533g | 1533 | UCAUUAUUAAUUUUAUUA | 18 | 1 | 0.0297596 |
| pvu-miR1533m | 1533 | UCAUUAUUUUUUAUAUUAU | 19 | 1 | 0.0297596 |
| pvu-miR156a | 156 | GACAGAAGAGAGAAAGCAG | 19 | 1 | 0.0297596 |
| pvu-miR169b | 169 | GCAGUCUCCUUGGAUA | 16 | 1 | 0.0297596 |
| pvu-miR1860a | 1860 | AGAUCUGUAGGCUGGUUUUC | 20 | 1 | 0.0297596 |
| pvu-miR2082 | 2082 | UGUGUGUUCUUCUUCUUCUU | 20 | 1 | 0.0297596 |
| pvu-miR396a | 396 | UCCACGACCUUCUUGAAU | 18 | 1 | 0.0297596 |
| pvu-miR5041a | 5041 | UUGAGCAAGUUGAAGAUG | 18 | 1 | 0.0297596 |
| pvu-miR5140a | 5140 | GCUGGUGAAGAUUUGG | 16 | 1 | 0.0297596 |
| pvu-miR5555a | 5555 | UCUAAGAGUGGAAUAUGACU | 20 | 1 | 0.0297596 |
| pvu-miR5721b | 5721 | AAAAAUGGAGUGAGAAAU | 18 | 1 | 0.0297596 |
| pvu-miR5998b | 5998 | AGUUUUUGUUUUGUUUUGU | 19 | 1 | 0.0297596 |
| pvu-miR771a | 771 | CAUGAAGAUAUGAGGAGCC | 19 | 1 | 0.0297596 |
| pvu-miR848c | 848 | UUUGACAAGGGAUUGC | 16 | 1 | 0.0297596 |
| pvu-miR848d | 848 | UUGACAUGGGUUUGC | 15 | 1 | 0.0297596 |
| pvu-miR902b | 902 | CUAUGUUUCAGAUCCUUCU | 19 | 1 | 0.0297596 |
| pvu-miR919a | 919 | AUCUCGGUCGAGCAUCUCGAU | 21 | 1 | 0.0297596 |

^a^Transcript per million reads.
