# Supplementary material for: Computational prediction of miRNAs and their targets in Phaseolus vulgaris using simple sequence repeat signatures
Source: BMC Plant Biol. 2015 Jun 12;15:140. doi: 10.1186/s12870-015-0516-3 (PMC4464996; doi:10.1186/s12870-015-0516-3)
Supplement: Supplementary file 4 — Predicted miRNAs of A. thaliana. [file 12870_2015_516_MOESM4_ESM.docx]

# **Table S4: Predicted miRNAs of *A. thaliana***

| Identifier | miRNA Sequence | Identifier | miRNA Sequence |
| --- | --- | --- | --- |
| ath-miR173-5p | UUCGCUUGCAGAGAGAAAUCAC | ath-miR862-3p | AUAUGCUGGAUCUACUUGAAG |
| ath-miR395a | CUGAAGUGUUUGGGGGAACUC | ath-miR164b-5p | UGGAGAAGCAGGGCACGUGCA |
| ath-miR5021 | UGAGAAGAAGAAGAAGAAAA | ath-miR400 | UAUGAGAGUAUUAUAAGUCAC |
| ath-miR157c-3p | GCUCUCUAUACUUCUGUCACC | ath-miR169g-5p | UGAGCCAAGGAUGACUUGCCG |
| ath-miR167a-3p | GAUCAUGUUCGCAGUUUCACC | ath-miR172c | AGAAUCUUGAUGAUGCUGCAG |
| ath-miR156a-3p | GCUCACUGCUCUUUCUGUCAGA | ath-miR165a-3p | UCGGACCAGGCUUCAUCCCCC |
| ath-miR169g-3p | UCCGGCAAGUUGACCUUGGCU | ath-miR399c-3p | UGCCAAAGGAGAGUUGCCCUG |
| ath-miR169b-3p | GGCAAGUUGUCCUUCGGCUACA | ath-miR399f | UGCCAAAGGAGAUUUGCCCGG |
| ath-miR158a-3p | UCCCAAAUGUAGACAAAGCA | ath-miR160a-5p | UGCCUGGCUCCCUGUAUGCCA |
| ath-miR397b | UCAUUGAGUGCAUCGUUGAUG | ath-miR397a | UCAUUGAGUGCAGCGUUGAUG |
| ath-miR5658 | AUGAUGAUGAUGAUGAUGAAA | ath-miR159a | UUUGGAUUGAAGGGAGCUCUA |
| ath-miR5020a | UGGAAGAAGGUGAGACUUGCA | ath-miR169d | UGAGCCAAGGAUGACUUGCCG |
| ath-miR396b-5p | UUCCACAGCUUUCUUGAACUU | ath-miR408-3p | AUGCACUGCCUCUUCCCUGGC |
| ath-miR166e-3p | UCGGACCAGGCUUCAUUCCCC | ath-miR862-5p | UCCAAUAGGUCGAGCAUGUGC |
| ath-miR771 | UGAGCCUCUGUGGUAGCCCUCA | ath-miR390b-5p | AAGCUCAGGAGGGAUAGCGCC |
| ath-miR395d | CUGAAGUGUUUGGGGGAACUC | ath-miR169f-3p | GCAAGUUGACCUUGGCUCUGC |
| ath-miR847 | UCACUCCUCUUCUUCUUGAUG | ath-miR395c | CUGAAGUGUUUGGGGGGACUC |
| ath-miR171c-5p | AGAUAUUGGUGCGGUUCAAUC | ath-miR4245 | ACAAAGUUUUAUACUGACAAU |
| ath-miR161.2 | UCAAUGCAUUGAAAGUGACUA | ath-miR854a | GAUGAGGAUAGGGAGGAGGAG |
| ath-miR399c-5p | GGGCAUCUUUCUAUUGGCAGG | ath-miR169m | UAGCCAAGGAUGACUUGCCUG |
| ath-miR156f-5p | UGACAGAAGAGAGUGAGCAC | ath-miR399d | UGCCAAAGGAGAUUUGCCCCG |
| ath-miR166f | UCGGACCAGGCUUCAUUCCCC | ath-miR390a-5p | AAGCUCAGGAGGGAUAGCGCC |
| ath-miR5641 | UGGAAGAAGAUGAUAGAAUUA | ath-miR157a-5p | UUGACAGAAGAUAGAGAGCAC |
| ath-miR416 | GGUUCGUACGUACACUGUUCA | ath-miR403-3p | UUAGAUUCACGCACAAACUCG |
| ath-miR160c-3p | CGUACAAGGAGUCAAGCAUGA | ath-miR846-3p | UUGAAUUGAAGUGCUUGAAUU |
| ath-miR168b-5p | UCGCUUGGUGCAGGUCGGGAA | ath-miR157d | UGACAGAAGAUAGAGAGCAC |
| ath-miR166b-3p | UCGGACCAGGCUUCAUUCCCC | ath-miR166a-5p | GGACUGUUGUCUGGCUCGAGG |
| ath-miR167d | UGAAGCUGCCAGCAUGAUCUGG | ath-miR413 | AUAGUUUCUCUUGUUCUGCAC |
| ath-miR860 | UCAAUAGAUUGGACUAUGUAU | ath-miR157b-3p | GCUCUCUAGCCUUCUGUCAUC |
| ath-miR472-3p | UUUUUCCUACUCCGCCCAUACC | ath-miR157c-5p | UUGACAGAAGAUAGAGAGCAC |
| ath-miR834 | UGGUAGCAGUAGCGGUGGUAA | ath-miR159b-3p | UUUGGAUUGAAGGGAGCUCUU |
| ath-miR168a-5p | UCGCUUGGUGCAGGUCGGGAA | ath-miR172d-3p | AGAAUCUUGAUGAUGCUGCAG |
| ath-miR172e-3p | GGAAUCUUGAUGAUGCUGCAU | ath-miR868-3p | CUUCUUAAGUGCUGAUAAUGC |
| ath-miR156i | UGACAGAAGAGAGAGAGCAG | ath-miR858b | UUCGUUGUCUGUUCGACCUUG |
| ath-miR824-5p | UAGACCAUUUGUGAGAAGGGA | ath-miR156h | UGACAGAAGAAAGAGAGCAC |
| ath-miR833b | UGUUUGUUGACAUCGGUCUAG | ath-miR172e-5p | GCAGCACCAUUAAGAUUCAC |
| ath-miR399a | UGCCAAAGGAGAUUUGCCCUG | ath-miR829-5p | ACUUUGAAGCUUUGAUUUGAA |
| ath-miR827 | UUAGAUGACCAUCAACAAACU | ath-miR5634 | AGGGACUUUGUGAAUUUAGGG |
| ath-miR167b | UGAAGCUGCCAGCAUGAUCUA | ath-miR845a | CGGCUCUGAUACCAAUUGAUG |
| ath-miR163 | UUGAAGAGGACUUGGAACUUCGAU | ath-miR172d-5p | GCAACAUCUUCAAGAUUCAGA |
| ath-miR156c-5p | UGACAGAAGAGAGUGAGCAC | ath-miR391-5p | UUCGCAGGAGAGAUAGCGCCA |
| ath-miR167a-5p | UGAAGCUGCCAGCAUGAUCUA | ath-miR396a-5p | UUCCACAGCUUUCUUGAACUG |
| ath-miR390a-3p | CGCUAUCCAUCCUGAGUUUCA | ath-miR403-5p | UGUUUUGUGCUUGAAUCUAAUU |
| ath-miR840-3p | UUGUUUAGGUCCCUUAGUUUC | ath-miR169n | UAGCCAAGGAUGACUUGCCUG |
| ath-miR169a-5p | CAGCCAAGGAUGACUUGCCGA | ath-miR162a-5p | UGGAGGCAGCGGUUCAUCGAUC |
| ath-miR854d | GAUGAGGAUAGGGAGGAGGAG | ath-miR169i | UAGCCAAGGAUGACUUGCCUG |
| ath-miR393a-5p | UCCAAAGGGAUCGCAUUGAUCC | ath-miR393a-3p | AUCAUGCUAUCUCUUUGGAUU |
| ath-miR840-5p | ACACUGAAGGACCUAAACUAAC | ath-miR398b-5p | AGGGUUGAUAUGAGAACACAC |
| ath-miR398c-5p | AGGGUUGAUAUGAGAACACAC | ath-miR858a | UUUCGUUGUCUGUUCGACCUU |
| ath-miR5629 | UUAGGGUAGUUAACGGAAGUUA | ath-miR2112-5p | CGCAAAUGCGGAUAUCAAUGU |
| ath-miR319b | UUGGACUGAAGGGAGCUCCCU | ath-miR833a-3p | UAGACCGAUGUCAACAAACAAG |
| ath-miR319a | UUGGACUGAAGGGAGCUCCCU | ath-miR393b-5p | UCCAAAGGGAUCGCAUUGAUCC |
| ath-miR156e | UGACAGAAGAGAGUGAGCAC | ath-miR857 | UUUUGUAUGUUGAAGGUGUAU |
| ath-miR166a-3p | UCGGACCAGGCUUCAUUCCCC | ath-miR5020c | UGGCAUGGAAGAAGGUGAGAC |
| ath-miR399b | UGCCAAAGGAGAGUUGCCCUG | ath-miR395b | CUGAAGUGUUUGGGGGGACUC |
| ath-miR158a-5p | CUUUGUCUACAAUUUUGGAAA | ath-miR2111b-5p | UAAUCUGCAUCCUGAGGUUUA |
| ath-miR162b-3p | UCGAUAAACCUCUGCAUCCAG | ath-miR166b-5p | GGACUGUUGUCUGGCUCGAGG |
| ath-miR169h | UAGCCAAGGAUGACUUGCCUG | ath-miR854c | GAUGAGGAUAGGGAGGAGGAG |
| ath-miR171b-5p | AGAUAUUAGUGCGGUUCAAUC | ath-miR169e | UGAGCCAAGGAUGACUUGCCG |
| ath-miR781b | UUAGAGUUUUCUGGAUACUUA | ath-miR8177 | GUGUGAUGAUGUGUCAUUUAUA |
| ath-miR171a-3p | UGAUUGAGCCGCGCCAAUAUC | ath-miR846-5p | CAUUCAAGGACUUCUAUUCAG |
| ath-miR2111a-5p | UAAUCUGCAUCCUGAGGUUUA | ath-miR825 | UUCUCAAGAAGGUGCAUGAAC |
| ath-miR157a-3p | GCUCUCUAGCCUUCUGUCAUC | ath-miR828 | UCUUGCUUAAAUGAGUAUUCCA |
| ath-miR396b-3p | GCUCAAGAAAGCUGUGGGAAA | ath-miR839-5p | UACCAACCUUUCAUCGUUCCC |
| ath-miR171b-3p | UUGAGCCGUGCCAAUAUCACG | ath-miR399e | UGCCAAAGGAGAUUUGCCUCG |
| ath-miR842 | UCAUGGUCAGAUCCGUCAUCC | ath-miR160b | UGCCUGGCUCCCUGUAUGCCA |
| ath-miR394b-5p | UUGGCAUUCUGUCCACCUCC | ath-miR398c-3p | UGUGUUCUCAGGUCACCCCUG |
| ath-miR829-3p.1 | AGCUCUGAUACCAAAUGAUGGAAU | ath-miR160a-3p | GCGUAUGAGGAGCCAUGCAUA |
| ath-miR172b-5p | GCAGCACCAUUAAGAUUCAC | ath-miR172b-3p | AGAAUCUUGAUGAUGCUGCAU |
| ath-miR169k | UAGCCAAGGAUGACUUGCCUG | ath-miR390b-3p | CGCUAUCCAUCCUGAGUUCC |
| ath-miR170-5p | UAUUGGCCUGGUUCACUCAGA | ath-miR164a | UGGAGAAGCAGGGCACGUGCA |
| ath-miR162a-3p | UCGAUAAACCUCUGCAUCCAG | ath-miR851-3p | UGGGUGGCAAACAAAGACGAC |
| ath-miR169b-5p | CAGCCAAGGAUGACUUGCCGG | ath-miR837-5p | AUCAGUUUCUUGUUCGUUUCA |
| ath-miR852 | AAGAUAAGCGCCUUAGUUCUG | ath-miR156d-5p | UGACAGAAGAGAGUGAGCAC |
| ath-miR837-3p | AAACGAACAAAAAACUGAUGG | ath-miR169j | UAGCCAAGGAUGACUUGCCUG |
| ath-miR169a-3p | GGCAAGUUGUCCUUGGCUAC | ath-miR319c | UUGGACUGAAGGGAGCUCCUU |
| ath-miR419 | UUAUGAAUGCUGAGGAUGUUG | ath-miR835-5p | UUCUUGCAUAUGUUCUUUAUC |
| ath-miR166g | UCGGACCAGGCUUCAUUCCCC | ath-miR164c-5p | UGGAGAAGCAGGGCACGUGCG |
| ath-miR156a-5p | UGACAGAAGAGAGUGAGCAC | ath-miR156c-3p | GCUCACUGCUCUAUCUGUCAGA |
| ath-miR5648-3p | AUCUGAAGAAAAUAGCGGCAU | ath-miR774b-5p | UGAGAUGAAGAUAUGGGUGAU |
| ath-miR156b-5p | UGACAGAAGAGAGUGAGCAC | ath-miR472-5p | AUGGUCGAAGUAGGCAAAAUC |
| ath-miR166c | UCGGACCAGGCUUCAUUCCCC | ath-miR171c-3p | UUGAGCCGUGCCAAUAUCACG |
| ath-miR394a | UUGGCAUUCUGUCCACCUCC | ath-miR162b-5p | UGGAGGCAGCGGUUCAUCGAUC |
| ath-miR167c-5p | UAAGCUGCCAGCAUGAUCUUG | ath-miR171a-5p | UAUUGGCCUGGUUCACUCAGA |
| ath-miR156j | UGACAGAAGAGAGAGAGCAC | ath-miR414 | UCAUCUUCAUCAUCAUCGUCA |
| ath-miR395e | CUGAAGUGUUUGGGGGAACUC | ath-miR398b-3p | UGUGUUCUCAGGUCACCCCUG |
| ath-miR168b-3p | CCCGUCUUGUAUCAACUGAAU | ath-miR160c-5p | UGCCUGGCUCCCUGUAUGCCA |
| ath-miR824-3p | CCUUCUCAUCGAUGGUCUAGA | ath-miR2111a-3p | GUCCUCGGGAUGCGGAUUACC |
| ath-miR396a-3p | GUUCAAUAAAGCUGUGGGAAG | ath-miR157b-5p | UUGACAGAAGAUAGAGAGCAC |
| ath-miR165a-5p | GGAAUGUUGUCUGGAUCGAGG | ath-miR166d | UCGGACCAGGCUUCAUUCCCC |
| ath-miR854e | GAUGAGGAUAGGGAGGAGGAG | ath-miR170-3p | UGAUUGAGCCGUGUCAAUAUC |
| ath-miR169l | UAGCCAAGGAUGACUUGCCUG | ath-miR166e-5p | GGAAUGUUGUCUGGCACGAGG |
| ath-miR426 | UUUUGGAAAUUUGUCCUUACG | ath-miR395f | CUGAAGUGUUUGGGGGGACUC |
| ath-miR398a-3p | UGUGUUCUCAGGUCACCCCUU | ath-miR169f-5p | UGAGCCAAGGAUGACUUGCCG |
| ath-miR394b-3p | AGGUGGGCAUACUGCCAAUAG | ath-miR158b | CCCCAAAUGUAGACAAAGCA |
| ath-miR161.1 | UGAAAGUGACUACAUCGGGGU | ath-miR829-3p.2 | CAAAUUAAAGCUUCAAGGUAG |
| ath-miR172a | AGAAUCUUGAUGAUGCUGCAU | ath-miR854b | GAUGAGGAUAGGGAGGAGGAG |
| ath-miR845b | UCGCUCUGAUACCAAAUUGAUG | ath-miR781a | UUAGAGUUUUCUGGAUACUUA |
| ath-miR165b | UCGGACCAGGCUUCAUCCCCC | ath-newmiR1 | UUGUUUUCCUUUUAUAUCUCA |
| ath-miR168a-3p | CCCGCCUUGCAUCAACUGAAU | ath-newmiR2 | UUGAAAUCUUUAAAUUCUUCAA |
| ath-miR164c-3p | CACGUGUUCUACUACUCCAAC | ath-newmiR3 | CAACAACAACAAGAAGACGAAGA |
| ath-miR415 | AACAGAGCAGAAACAGAACAU | ath-newmiR4 | CAAUGAAGUGUUCAGGAGAAC |
| ath-miR393b-3p | AUCAUGCGAUCUCUUUGGAUU | ath-newmiR5 | AAAACAUCUUAUAAUUUGGA |
| ath-miR5650 | UUGUUUUGGAUCUUAGAUACA | ath-newmiR6 | UUUGGAAAAAUAUGGAUCAU |
| ath-miR159b-5p | GAGCUCCUUGAAGUUCAAUGG | ath-newmiR7 | UGAAUGAUGCCUGGCUCGAGA |
| ath-miR159c | UUUGGAUUGAAGGGAGCUCCU | ath-newmiR8 | UUGUUUCUUGUUUUGUUCA |
| ath-miR156b-3p | UGCUCACCUCUCUUUCUGUCAGU | ath-newmiR9 | AAUCAAUUAUAAAAUAACA |
| ath-miR391-3p | ACGGUAUCUCUCCUACGUAGC | ath-newmiR10 | CAGUCUCUUUGUCUAUC |
| ath-miR418 | UAAUGUGAUGAUGAACUGACC | ath-newmiR11 | UACUCCCUUUGUUCCAUAAA |
| ath-miR169c | CAGCCAAGGAUGACUUGCCGG | ath-newmiR12 | UCAGUCGCAGGAGAGAUGA |
| ath-miR164b-3p | CAUGUGCCCAUCUUCACCAUC | ath-newmiR13 | AUUCAAAAAAAGAACUAGAA |
| ath-miR156f-3p | GCUCACUCUCUAUCCGUCACC | ath-newmiR14 | UUGUUUGUGGUGAUGUC |
| ath-miR838 | UUUUCUUCUACUUCUUGCACA | ath-newmiR15 | UAGCCAAGGAGACUGCC |
| ath-miR156g | CGACAGAAGAGAGUGAGCAC | ath-newmiR16 | CUUUAUGGGACAGAGGGAGUA |
| ath-miR835-3p | UGGAGAAGAUACGCAAGAAAG |  |  |
