# Supplementary material for: Computational prediction of miRNAs and their targets in Phaseolus vulgaris using simple sequence repeat signatures
Source: BMC Plant Biol. 2015 Jun 12;15:140. doi: 10.1186/s12870-015-0516-3 (PMC4464996; doi:10.1186/s12870-015-0516-3)
Supplement: Supplementary file 5 — Predicted miRNAs of G. max. [file 12870_2015_516_MOESM5_ESM.docx]

# **Table S5: Predicted miRNAs of *G.max***

| Identifier | miRNA Sequence | Identifier | miRNA Sequence |
| --- | --- | --- | --- |
| gma-miR5038a | UGAGAAUUUGGCCUCUGUCCA | gma-miR5037d | CGGGAGCCUAUGAAGGUUAAC |
| gma-miR5672 | CAUGGUAGUGGAAGAAAUGGA | gma-miR5781 | CUGAAACUGAGACUGCAUCUGG |
| gma-miR862b | GCUGGAUGUCUUUGAAGGA | gma-miR5375 | ACUAUAGAAGUACUUGUGGAGC |
| gma-miR156v | UGACAGAAGAGAGUGAGCAC | gma-miR5032 | AGAGCCACUUUUGGGUUCCCUAU |
| gma-miR1520l | AAUCAGAACAUGACACGUGAUAGU | gma-miR396h | UCCACAGCUUUCUUGAACUG |
| gma-miR169a | CAGCCAAGGAUGACUUGCCGG | gma-miR5768 | AAGUGCAAUACUGAUCUUCGGAAC |
| gma-miR2606a | AAAAGCACUUAAGGAACGGUA | gma-miR1508b | UAGAAAGGGGAAUAGCAGUUG |
| gma-miR396k | GCUCAAGAAAGCUGUGGGAGA | gma-miR4382 | UAUGUUAACUGAUUUCAUGGAU |
| gma-miR403b | UUAGAUUCACGCACAAACUUG | gma-miR408b-5p | CUGGGAACAGGCAGGGCACG |
| gma-miR5371-5p | UAGGAAUUAGUCACUCAGAUC | gma-miR167e | UGAAGCUGCCAGCAUGAUCUU |
| gma-miR482b-5p | UAUGGGGGGAUUGGGAAGGAAU | gma-miR390d | AAGCUCAGGAGGGAUAGCACC |
| gma-miR171i-3p | UUGAGCCGUGCCAAUAUCACG | gma-miR319f | UUGGACUGAAGGGGCCUCUU |
| gma-miR1532 | AACACGCUAAGCGAGAGGAGCUC | gma-miR396i-3p | GUUCAAUAAAGCUGUGGGAAG |
| gma-miR408c-5p | CAGGGGAACAGGCAGAGCAUG | gma-miR156z | AUUGGAGUGAAGGGAGCU |
| gma-miR1524 | CGAGUCCGAGGAAGGAACUCC | gma-miR4371c | GACGUGACAGACGGAAUAUCACAU |
| gma-miR156m | UUGACAGAAGAUAGAGAGCAC | gma-miR4375 | UACCACUAGUGGUCGCGCCUGGCA |
| gma-miR408d | UGCACUGCCUCUUCCCUGGC | gma-miR4380b | UAUGGUCAUACGGAUUGUUGAU |
| gma-miR4358 | CAGUGCAUGACUAUAUCGCCAG | gma-miR4388 | AAUCUUAGGGACCAAAUUGACAGC |
| gma-miR4398 | UGUCAGCGGAGUGAGAAGACGAAA | gma-miR4364b | UAACAACAGCGGAAGAACCUUCUU |
| gma-miR160f | UGCCUGGCUCCCUGUAUGCCA | gma-miR5675 | UAGAGACGACAACAAUGGAAA |
| gma-miR393b | UUUGGGAUCAUGCUAUCCCUU | gma-miR172f | AGAAUCUUGAUGAUGCUGCA |
| gma-miR166d | UCGGACCAGGCUUCAUUCCCC | gma-miR1520d | AUCAGAACAUGACACGUGACAA |
| gma-miR1520h | AACGUCCAAUCAGAACGUGACAUG | gma-miR4408 | UAACAACAUUGGAUGAGGGUUGGA |
| gma-miR171b-5p | ACGGCGUGAUAUUGGUACGGCUC | gma-miR1536 | AAGCAGAGACAAAUGUGUUUA |
| gma-miR395e | UGAAGUGUUUGGGGGAACUUU | gma-miR4394 | AAUGGACUAAAGAGAAAGGGGCCG |
| gma-miR4353 | CAAGUCGUAGCCGGUGUUAUUACU | gma-miR4359b | AACGCGUGAUAUGUUAACAUCGGU |
| gma-miR4996 | UAGAAGCUCCCCAUGUUCUC | gma-miR1520k | AAUCAGAACAUGACACAUGACAGU |
| gma-miR4379 | UAGAGUGUAUACUGUGAGAGGCCU | gma-miR168a | UCGCUUGGUGCAGGUCGGGAA |
| gma-miR169l-5p | CAGCCAAGAAUGACUUGCCGG | gma-miR4343b | UCUUACAGAUCAAGUUGAUUCGGA |
| gma-miR2118b-5p | GGAGAUGGGAGGGUCGGUAAAG | gma-miR1531 | UCGUCCAUAUGGGAAGACUUGUC |
| gma-miR156b | UGACAGAAGAGAGAGAGCACA | gma-miR5775 | AUAAGCUCUUUUGAGAGCUUC |
| gma-miR394a-5p | UUGGCAUUCUGUCCACCUCC | gma-miR5763a | UGAACUAUACAAAGACGGUUA |
| gma-miR2108a | UUAAUGUGUUGUGUUUGUCGG | gma-miR169p | UGAGCCAAGGAUGACUUGCCG |
| gma-miR1513c | UAUGAGAGAAAGCCAUGAC | gma-miR5776 | AACUUGGGCUGAGCUUAGGUG |
| gma-miR393i | UUCCAAAGGGAUCGCAUUGAUC | gma-miR397b-5p | UCAUUGAGUGCAGCGUUGAUG |
| gma-miR4366 | CUACUUAGUAGAGAUUUGUUGG | gma-miR5037a | GCCUCAAAGGCUUCCACUACUG |
| gma-miR166t | UCGGACCAGGCUUCAUUCCC | gma-miR156ab | AUUUAAGUGAUGGGAGCUCCG |
| gma-miR5679 | UUGGUGACCCAGAAGAAGUUGA | gma-miR4352b | UAAAAUGUAGACAUUCUAAGACGG |
| gma-miR4389 | UCGGUCGGACCGAUCCAAUCGGAA | gma-miR5668 | AGCAAUGGAAUUAUAGACUGC |
| gma-miR159d | AGCUGCUUAGCUAUGGAUCCC | gma-miR172d | GGAAUCUUGAUGAUGCUGCAGCAG |
| gma-miR5784 | AAUUAGCUAAUGGUUAGCUAA | gma-miR169e | AGCCAAGGAUGACUUGCCGG |
| gma-miR4411 | UUAUUGUAACUAAUUUGUCGGU | gma-miR167j | UGAAGCUGCCAGCAUGAUCUG |
| gma-miR4401a | ACAACGUCUUUGAAAGUAGGCAUU | gma-miR4387e | UGUUAGUGAUAAGGCGUGAUG |
| gma-miR4997 | GAUCGUCAAGCGCGAAGAUGAGG | gma-miR5031 | UUAAUGAUUAACAUCUAAUUU |
| gma-miR4412-3p | AGUGGCGUAGAUCCCCACAAC | gma-miR4385 | AAUCGAUGUAGAAAAGUGAUUGGU |
| gma-miR4404 | AUUCGUGGAAGACUGGCGGAUCAA | gma-miR5782 | UAGCUGGUAGGAGAAGUUCAG |
| gma-miR530c | UGCAUUUGCACCUGCACUUUA | gma-miR156r | CUGACAGAAGAUAGAGAGCAU |
| gma-miR171i-5p | AUAAGAAAGCAAUGCUCAAA | gma-miR1512c | UAACUGAACAUUCUUAGAGCAU |
| gma-miR4415b-3p | UUGAUUCUCAUCACAACAUGG | gma-miR1535a | CUUGUUUGUGGUGAUGUCU |
| gma-miR1521a | CUGUUAAUGGAAAAUGUUGA | gma-miR1509a | UUAAUCAAGGAAAUCACGGUCG |
| gma-miR4390 | UCGUACUCGUCGGGUAUCGGGUAU | gma-miR1530 | UUUUCACAUAAAUUAAAAUAU |
| gma-miR164a | UGGAGAAGCAGGGCACGUGCA | gma-miR1507a | UCUCAUUCCAUACAUCGUCUGA |
| gma-miR5043 | UGUCCCCUUCUCUGCACCACC | gma-miR4364a | CGCGAGAUCGCACGGAAGAAGGUU |
| gma-miR4344 | AAGUAGACAUUCUAAGACGUUGCU | gma-miR4349 | UAUUGGCUAGAGAUAAGACAAAGA |
| gma-miR1516d | GUACUUGUGGCUUGUAUCCAA | gma-miR390b-3p | UACUUGGCGCUAUCUAUCUUGA |
| gma-miR1508c | UAGAAAGGGAAAUAGCAGUUG | gma-miR319n | UUUGGACCGAAGGGAGCCCCU |
| gma-miR166m | CGGACCAGGCUUCAUUCCCC | gma-miR1520n | UCAAUCAGAACAUGACACGUGACA |
| gma-miR172b-5p | GUAGCAUCAUCAAGAUUCAC | gma-miR4401b | UCAAAGACGUUGCUGAGGUAA |
| gma-miR4363 | CGAUUACCAGAAGGCUUAUUAG | gma-miR171d | UGAUUGAGUCGUGUCAAUAUC |
| gma-miR3522 | AGACCAAAUGAGCAGCUGA | gma-miR156g | ACAGAAGAUAGAGAGCACAG |
| gma-miR1520m | AAUCAGAACAUGACAUGUGACAAU | gma-miR5225 | CCUGUCGUAGGAGAGAUGACGC |
| gma-miR319i | UUGGACUGAAGGGGAGCUCCUUC | gma-miR2107 | CAAACCUCCGUAGCCUGUAUC |
| gma-miR160a-3p | GCGUAUGAGGAGCCAAGCAUA | gma-miR4993 | GAGCGGCGGCGGUGGAGGAUG |
| gma-miR396a-5p | UUCCACAGCUUUCUUGAACUG | gma-miR169b | CAGCCAAGGAUGACUUGCCGA |
| gma-miR399e | UGCCAAAGGAGAUUUGCCCAG | gma-miR1520f | CAAUCAGAACAUGACACAUGACAA |
| gma-miR4383 | UAUUGGAUCUCAGUUGAACCGGUC | gma-miR5039 | CCCUUUUUUAAUCGUUGCAUG |
| gma-miR4370 | AGUAGACUCGUCCGAUUUUGCGUA | gma-miR4413b | UAAGAGAAUUGUAAGUCACU |
| gma-miR2111a | GUCCUUGGGAUGCAGAUUACG | gma-miR171k-5p | CGAUGUUGGUGAGGUUCAAUC |
| gma-miR393d | UCCAAAGGGAUCGCAUUGAUCC | gma-miR172g | GCAGCACCAUCAAGAUUCAC |
| gma-miR5773 | UUUUUAAAAGGUUCAGUUAGGU | gma-miR1513a-3p | UUUAAAUGUGUAUAAGUCAUGGU |
| gma-miR4357 | CAGUCGUGUGAUUGUACGGUUCAU | gma-miR1520j | AAGAACGUGACACAUGACAAUCAA |
| gma-miR167a | UGAAGCUGCCAGCAUGAUCUA | gma-miR4359a | AACGAAGUGACUCUAACAUCGGUU |
| gma-miR5770a | UUAGGACUAUGGUUUGGACGA | gma-miR1529 | UUAAAGGAAACAAUUAAUCGUUA |
| gma-miR159e-3p | UUUGGAUUGAAGGGAGCUCUA | gma-miR4413a | AAGAGAAUUGUAAGUCACUG |
| gma-miR319b | UUGGACUGAAGGGAGCUCCC | gma-miR169s | AAGCCAAGGAUGACUUGCCGG |
| gma-miR5764 | UCCAUUCGCGGACAUGAUGGAU | gma-miR396e | UUCCACAGCUUUCUUGAACUGU |
| gma-miR159f-5p | GAGUUCCCUGCACUCCAAGUC | gma-miR5770b | UUAGGACUAUGGUUUGGACAA |
| gma-miR398a | UGUGUUCUCAGGUCACCCCUU | gma-miR1514a | UUCAUUUUUAAAAUAGGCAUU |
| gma-miR167g | UGAAGCUGCCAGCAUGAUCUGA | gma-miR5767 | UGGAGGACCUUUGAAGGUGCA |
| gma-miR166h-3p | UCUCGGACCAGGCUUCAUUCC | gma-miR482a-5p | AGAAUUUGUGGGAAUGGGCUGA |
| gma-miR4376a-3p | AGCAUCAUAUCUCCUGCAUAG | gma-miR396f | AGCUUUCUUGAACUUCUUAUGCCUA |
| gma-miR1520c | UUCAAUAAGAACGUGACACGUGA | gma-miR171j-5p | UAUUGGCCUGGUUCACUCAGA |
| gma-miR5044 | GUAGUGGAUGCCUAGAGGUCCA | gma-miR5783 | GACGACGACGGGGAGGACGCGC |
| gma-miR171c-5p | AGAUAUUGGUGCGGUUCAAUC | gma-miR6299 | AUUUAAAAUUAUUGAUUUGUCA |
| gma-miR4415b-5p | AAGUUGUGAUGGGAAUCAAUGGCA | gma-miR1518 | UGUGUUGUAAAGUGAAUAUCA |
| gma-miR1516c | AAUGUCUGGGCUUAGCGAGGCGGU | gma-miR4412-5p | UGUUGCGGGUAUCUUUGCCUC |
| gma-miR4377 | UACGUCAUCGCUGAAUGGAAGACG | gma-miR5377 | CUGAAGGAUCGAUGUAGAAUGCU |
| gma-miR171r | CGAGCCGAAUCAAUACCACUC | gma-miR4998 | AGUUUCGUGACUACAACUUCUG |
| gma-miR4410 | UAUGUUGAUCCGUAUGAGUCGUAC | gma-miR4416b | UGGGUGAGAGAAACGCGUAUC |
| gma-miR166h-5p | GGAAUGUUGUUUGGCUCGAGG | gma-miR4407 | CAGAGGAAGCAGCACUUGUACC |
| gma-miR169v | CAGCCAAGGAUGACUUGCC | gma-miR4397-5p | CAUCGUUGACGCUGACUGUACG |
| gma-miR156n | UUGACAGAAGAGAGUGAGCAC | gma-miR5373 | UCUCUUGAUUCUAGAUGAUGU |
| gma-miR169i-3p | CCGGUGCCAUCCCGUCUCAUA | gma-miR1516b | AGCUUCUCUACAGAAAAUAUA |
| gma-miR169l-3p | CGGGCAAGUUGUUUUUGGCUAC | gma-miR2108b | UUAAUGUGUUGUGUUUGUGAG |
| gma-miR159c | AUUGGAGUGAAGGGAGCUCCG | gma-miR6300 | GUCGUUGUAGUAUAGUGG |
| gma-miR4348 | AAACUUGUAAGAUGGUGACAUU | gma-miR2119 | UCAAAGGGAGUUGUAGGGGAA |
| gma-miR1525 | UGGGUUAAUUAAGUUUUUAGU | gma-miR1523a | AUGGGAUAAAUGUGAGCUCA |
| gma-miR160e | UGCCUGGCUCCCUGUAUGCC | gma-miR1512a | UAACUGAAAAUUCUUAAAGUAU |
| gma-miR4368b | AAGGACGGUACUUACGUAAGCAAC | gma-miR5778 | CGACGAACUCUUCGUCGGCAUC |
| gma-miR159f-3p | AUUGGAGUGAAGGGAGCUCCA | gma-miR4395 | UGGAUAGGAGUAUGGGCUUGAG |
| gma-miR4365 | AAGAACUUCUUCCGCGAGAUCGCA | gma-miR171m | UUGAGCCGCGUCAAUAUCUCA |
| gma-miR156s | UGACAGAAGAGAGUGAGCACU | gma-miR4347 | AAGCUUCUUACGGAUCAAGUUGAU |
| gma-miR5369 | UGAGAAAAGGAGGAUGUCA | gma-miR4360 | CAGUUGACGUACGUACGGAUUGAC |
| gma-miR5786 | UGUCGCAGGAUAGAGGGCACU | gma-miR1520b | GUGACAGUCAUCAUUUAAUAAGA |
| gma-miR1507c-5p | GAGGUGUUUGGGAUGAGAGAA | gma-miR4373 | AAGUUGACGUACGUACGGAUUGAC |
| gma-miR162c | UCGAUAAACCUCUGCAUCCAG | gma-miR4392 | UCUGCGAAAAUGUGAUUUCGGA |
| gma-miR4354 | CAAUUGGAUCGGUCCAACCGGC | gma-miR4387a | AACAAGACGUGAUGACGUGACACU |
| gma-miR1521b | GACUGUCACGUGUCAUAAUCAUA | gma-miR394b-3p | AGGUGGGCAUACUGUCAACU |
| gma-miR1515a | UCAUUUUGCGUGCAAUGAUCUG | gma-miR5037c | AGUGGAACUUUGAGGCCUGC |
| gma-miR4376-5p | UACGCAGGAGAGAUGACGCUGU | gma-miR1526 | CCGGAAGAGGAAAAUUAAGCAA |
| gma-miR2111b | UAAUCUGCAUCCUGAGGUUUA | gma-miR4355 | CACUGUUGUGCUGGGUGUACCA |
| gma-miR5370 | CUAAAGAUUGUCCAAAAGGAA | gma-miR1534 | UAUUUUGGGUAAAUAGUCAU |
| gma-miR319k | UUGGACUGAAGGGAGCUCCCU | gma-miR171k-3p | UUGAGCCGCGCCAAUAUCACU |
| gma-miR5035 | CUUCUAAACAUUUUUUCCCUUA | gma-miR5036 | AGAGGCCCUUGGGGAGGAGUAA |
| gma-miR4362 | CCUUAGGACAGACGUCAUGUAG | gma-miR169i-5p | UGAGCCGGGAUGGCUUGCCGGCA |
| gma-miR5378 | CAUCUGAAGGAUAGAACACAUA | gma-miR319d | UGGACUGAAGGGGAGCUCCUUC |
| gma-miR4416a | ACGGGUCGCUCUCACCUAGG | gma-miR4381 | UAUGUGACGGUAAACGGUGACAAG |
| gma-miR482d-3p | UCUUCCCUACACCUCCCAUACC | gma-miR5673 | CGUGGAAUCUCGCGGAAGACAU |
| gma-miR164b | UGGAGAAGCAGGGCACGUGC | gma-miR5041 | UUUCAUCUUCAACUUGCUCAA |
| gma-miR4396 | UGUAGUUUCUAAGACGAUGCUGAC | gma-miR1516a-3p | CAAAAGAGCUUAUGGCUUGUA |
| gma-miR4351 | AUUGGGAUUCAGUUGGAGUUGG | gma-miR159a-5p | GAGCUCCUUGAAGUCCAAUUG |
| gma-miR395l | AUGAAGUGUUUGGGGGAACUC | gma-miR4372a | UAAAAUCGUGACAUGUGACGGUCA |
| gma-miR5762 | UCAUAGGAGGAAUCAACUGGC | gma-miR1520p | AUGUUGUUAUUGGAUGAUGACGGU |
| gma-miR5670 | CAUCAUACCAUAUUUGCUUCAU | gma-miR5769 | UGAGGGAAAUGAAGACGACGA |
| gma-miR530a | UGCAUUUGCACCUGCACUUU | gma-miR1511 | AACCAGGCUCUGAUACCAUG |
| gma-miR5037b | AACCCUCAAAGGCUUCCUAG | gma-miR398c | UGUGUUCUCAGGUCGCCCCUG |
| gma-miR5667 | AAACAGAUCUAAAUGGAUUCC | gma-miR390e | AGCUCAGGAGGGAUAGCGCC |
| gma-miR1522 | UUUAUUGCUUAAAAUGAAAU | gma-miR4343a | AAAAAACUUACGGAUCAAGUUGAU |
| gma-miR169h | GGCGAGACAUCUUGGCUCAUU | gma-miR482e | UAUGGGGGGAUUGGGAAGGAA |
| gma-miR4368a | AAGACGGUACUUACCUCAGUAACA | gma-miR4340 | UGCAGAGAUAGGGACGCGCUUA |
| gma-miR1528 | AUAGAUUAGAUCAAUAUAUUAGU | gma-miR4406 | AUUGAUUCUGAGAGAACCGGUGUA |
| gma-miR4378b | UAGAACUGUCUUAGAAUGUGCUAC | gma-miR169n | CAGCCAAGGGUGAUUUGCCGG |
| gma-miR5674b | UAAUUGUGUUGUACAUUAUCA | gma-miR1507c-3p | CCUCAUUCCAAACAUCAUCU |
| gma-miR4399 | UUAACGAAAAAGGACUAACGAC | gma-miR4416c | CUGGGUGAGAGAAACACGUAU |
| gma-miR1509b | UUAAUCAAGGAAAUCACGGUU | gma-miR4371b | AAGUGAUGACGUGGUAGACGGAGU |
| gma-miR5676 | UCGACACCAUAUGUAGAGGCAG | gma-miR1510b-5p | AGGGAUAGGUAAAACAACUACU |
| gma-miR171p | UUGAGCCGCGUCAAUAUCUUA | gma-miR4380a | CGGAUUGUUGAUCCGUAUGUGCAU |
| gma-miR1520r | UGUCACAUCCUGGUUGGACAUGAA | gma-miR162a | UCGAUAAACCUCUGCAUCCA |
| gma-miR171j-3p | UGAUUGAGCCGUGCCAAUAUC | gma-miR1507b | UCUCAUUCCAUACAUCGUCUG |
| gma-miR4397-3p | UGUCAAAGAUGUGGCGAAUACU | gma-miR4367 | CUGAACCCUAGCGAAGUAAAUC |
| gma-miR171c-3p | UUGAGCCGUGCCAAUAUCACA | gma-miR4374b | UACUUUCAAAGACGUUGUUGAG |
| gma-miR5559 | UACUUGGUGAAUUGUUGGAUC | gma-miR5766 | UUGAGGCUGAGAAGAGGCAAG |
| gma-miR4342 | AAUCGACUUAGAAUGUAGGAUGGU | gma-miR4415a-5p | AAGUUGUGAUGAGAAUCAAUG |
| gma-miR167i | UCAUGCUGGCAGCUUCAACUGGU | gma-miR172c | GGAAUCUUGAUGAUGCUGCAG |
| gma-miR1520o | UCAUCGUCCAAUCAGAAUGUGACA | gma-miR5372 | UUGUUCGAUAAAACUGUUGUG |
| gma-miR1519 | UAAGUGUUGCAAAAUAGUCAUU | gma-miR1510a-3p | UUGUUGUUUUACCUAUUCCACCC |
| gma-miR1514b | UUCAUUUUUAAAAUAGACAUU | gma-miR5042 | UAUCUUGGAUCACAGCCCCAUU |
| gma-miR1508a | UCUAGAAAGGGAAAUAGCAGUUG | gma-miR159e-5p | GAGCUCCUUGAAGUCCAAUU |
| gma-miR169c | AAGCCAAGGAUGACUUGCCGA | gma-miR5777 | CUAGCAAUAAUGUUGGAUGCAC |
| gma-miR1520i | AACGUGACACGUGACGGUCAACAU | gma-miR1535b | CUUGUUUGUGGUGAUGUCUAG |
| gma-miR171h | AUUGAGACGAGCCGAAUCAAU | gma-miR1510b-3p | UGUUGUUUUACCUAUUCCACC |
| gma-miR5774b | GCUGGCGUCGACACGUGGCAU | gma-miR156f | UUGACAGAAGAGAGAGAGCACA |
| gma-miR5379 | AUGAAAAUCAUUCAUUAUGAUAUC | gma-miR172k | UGAAUCUUGAUGAUGCUGCAU |
| gma-miR5368 | GGACAGUCUCAGGUAGACA | gma-miR394a-3p | AGCUCUGUUGGCUACACUUU |
| gma-miR169u | CAGCCAAGGAUGACUUGCCGU | gma-miR4405 | AUUCUAAGACGGUUAUCUGGGACC |
| gma-miR5380b | GAAAAUGAAUGAUGAGGAUGGGGA | gma-miR4384 | AAUCAGACACUGCAUUCAAAGACG |
| gma-miR4403 | ACGGACACCGAACACGACACGGAC | gma-miR4387c | AGCGUGAUGACGUGACACUCCGUC |
| gma-miR4346 | GAAAGACCAAACGAGAAGCUGCAU | gma-miR1520e | CAAUAAGAACGUGACAUAUGACAG |
| gma-miR172h-5p | GCAGCAGCAUCAAGAUUCACA | gma-miR482c-3p | UUCCCAAUUCCGCCCAUUCCU |
| gma-miR4994 | GGUUAGCUCAAGGAUCUCAC | gma-miR4386 | UCGAAGGUUCUGGAGAGGACUGCA |
| gma-miR408c-3p | AUGCACUGCCUCUUCCCUGGC | gma-miR168b | UCGCUUGGUGCAGGUCGGG |
| gma-miR5376 | UGAAGAUUUGAAGAAUUUGGGA | gma-miR4992 | AUUCUAAGAUGGUUUUUGUUAG |
| gma-miR169r | UGAGCCAGGAUGGCUUGCCGGC | gma-miR169d | UGAGCCAAGGAUGACUUGCCGGU |
| gma-miR5374 | UUAUAGUCUGACAUCUGGAAU | gma-miR319c | UUGGACUGAAAGGAGCUCCU |
| gma-miR5034 | GGUACCCUUUCAGAUAGUCUCA | gma-miR482c-5p | AUUUGUGGGAAUGGGCUGAUUGG |
| gma-miR862a | UGCUGGAUGUCUUUGAAGGAAU | gma-miR396d | AAGAAAGCUGUGGGAGAAUAUGGC |
| gma-miR828b | UCUUGCUCAAAUGAGUAUUCCA | gma-newmiR1 | CAAGGGUAUGAUGUGCAAUCU |
| gma-miR319l | UUGGACUGAAGGGAGCUCCUUC | gma-newmiR2 | UUUGAAUGUCCAGAUACGUAC |
| gma-miR5371-3p | UCUCAGUGACUAAUUUCUAGA | gma-newmiR3 | UUUUGGACUGAAGGGAGCUCC |
| gma-miR4414 | AGCUGCUGACUCGUUGGCUC | gma-newmiR4 | UGUAAGUCCAUAUGUGCUCUCU |
| gma-miR166i-5p | GGAAUGUCGUCUGGUUCGAG | gma-newmiR5 | UGAUAUCCUUGAGCUAAUACA |
| gma-miR1513a-5p | UGAGAGAAAGCCAUGACUUAC | gma-newmiR6 | UGGAUGAUGUAGUUUUGAUUG |
| gma-miR4387d | AUGUCACUGAUUAGGCAUGAUGAU | gma-newmiR7 | GAAGGAAGUGUAGAGGGAUGAC |
| gma-miR390g | AAGCUCAGGAGGGAUAGCGCC | gma-newmiR8 | UGUUUAGAAGCUCAUAGAAUAGAU |
| gma-miR399c | UGCCAAAGGAGAGUUGCCCUG | gma-newmiR9 | CAAAGGAGAUUUGGACAACUC |
| gma-miR4402 | ACAUAUUAUGGGUCUCAGACGGAC | gma-newmiR10 | AGAAUUAAAUUUGGACCGUAUAAC |
| gma-miR5671 | CAUGGAAGUGAAUCGGGUGAC | gma-newmiR11 | GUAAUUUUAAACCUAAACCCUAAA |
| gma-miR395h | AUGAAGUGUUUGGGAGAACUC | gma-newmiR12 | UGUAGGUUCCAGUGAGGGAAA |
| gma-miR1516a-5p | CAAGUUAUAAGCUCUUUUGAGAG | gma-newmiR13 | UGAAACAUGAUGUGGACUCUUC |
| gma-miR5669 | CAAUGUAGUGUGGUAAGUGGUC | gma-newmiR14 | UGGGGCUUGAUCCAAGAUAGG |
| gma-miR5677 | UUUGGUCUUUAAUCAAGCUGA | gma-newmiR15 | AAAGUGUUUGAAUCUCAAUUAGAU |
| gma-miR393a | UCCAAAGGGAUCGCAUUGAUC | gma-newmiR16 | UAGAGAUAGUGUCAAAAUAGAA |
| gma-miR1523b | UCAUCGCUCCUGAGCUCACA | gma-newmiR17 | UGUUUAGUCAUGCAAGUUUAG |
| gma-miR5779 | CAAGUCCAAAGUAGGAAUGUUGCA | gma-newmiR18 | UGAGAACUUUAUCCAAACAGAG |
| gma-miR4995 | AGGCAGUGGCUUGGUUAAGGG | gma-newmiR19 | GUUGAGCAAGUUGAAGAUGAA |
| gma-miR4378a | AUAGGACUGUCUUAGAAUGGUGUA | gma-newmiR20 | AUGGAAUGGUUACUUAUGAAAAGA |
| gma-miR396j | AUUCAAGAUAGCUGUGGAAAA | gma-newmiR21 | UCCAACGAUGCGGGAGCUGC |
| gma-miR169j-5p | UAGCCAAGAAUGACUUGCCGG | gma-newmiR22 | UUAGCUUCUUUCACCUUUCCC |
| gma-miR171a | UGAGCCGUGCCAAUAUCACGA | gma-newmiR23 | AUGCCUAUUUUAAAAUGAAAA |
| gma-miR172i-3p | GGAAUCUUGAUGAUGCUGCAU | gma-newmiR24 | UCUUUUGAAUUUGACUAUUAG |
| gma-miR1527 | UAACUCAACCUUACAAAACC | gma-newmiR25 | CGAUUGCUGUCAUAACUGCUGC |
| gma-miR1533 | AUAAUAAAAAUAAUAAUGA | gma-newmiR26 | UGAAAGACAAACAAAGGUGGG |
| gma-miR4341 | UGUGUUGAAAGUUUAACAUGACGG | gma-newmiR27 | AUUGUCACGUGUCAUGUUCUGAUU |
| gma-miR2118b-3p | UUGCCGAUUCCACCCAUUCCU | gma-newmiR28 | UGUUAAACUUGCAAGAUGACA |
| gma-miR4409 | UAACAAGUGGGUUUGUUGACUG | gma-newmiR29 | UGAAGGUCUAGGAUAUUUUGU |
| gma-miR482a-3p | UCUUCCCAAUUCCGCCCAUUCCUA | gma-newmiR30 | UUGUGGCUGAAAUCACUGUUGC |
| gma-miR167h | AUCAUGCUGGCAGCUUCAACUGGU | gma-newmiR31 | GUAAUGAGUAGAAACAUUUAGAAG |
| gma-miR5040 | AUGAUAUAUAACAAGCAUGAG | gma-newmiR32 | CAUGCGAUGAUUUAAAUACUUU |
| gma-miR4393a | UGAGAAAAGGACGGCAGAAAAGCC | gma-newmiR33 | UACGGCUUAAGUUCAACUUUGGAG |
| gma-miR156p | UUGACAGAAGAAAGGGAGCAC | gma-newmiR34 | UCGUGAAUGAGAUUUGUGUUGCUU |
| gma-miR396a-3p | UUCAAUAAAGCUGUGGGAAG | gma-newmiR35 | UGAAGCUGCCAGCCUGAUCUUA |
| gma-miR166l | GGAAUGUUGUCUGGCUCGAGG | gma-newmiR36 | AAUCCAUUCAGAUCUGUUUCG |
| gma-miR171b-3p | CGAGCCGAAUCAAUAUCACUC | gma-newmiR37 | UAACAGACUUAGAUUAACAGA |
| gma-miR172a | AGAAUCUUGAUGAUGCUGCAU | gma-newmiR38 | UGGAUACACUCUUACUUUCUC |
| gma-miR169t | UAGCCAAGGAUGGACUUGCCUA | gma-newmiR39 | AAUGUGUUGAACUGAGUGAAGACA |
| gma-miR5785 | UAGUGUUGUCCUGUCGAACACGGA | gma-newmiR40 | UAAUACAGAAUUCGGAGACAAC |
| gma-miR4345 | UAAGACGGAACUUACAAAGAUU | gma-newmiR41 | GCUUUAAGAAUUUCAGUUAUG |
| gma-miR5678 | UUCCAUGAUAAGAUCUUUGAC | gma-newmiR42 | UAUAAGCAAGUAGAAUUUAAU |
| gma-miR4374a | UAAGACGGUCGUGAUGUCAGCA | gma-newmiR43 | UAUAGGCAUUAUUUUUUUCUUC |
| gma-miR2109 | UGCGAGUGUCUUCGCCUCUG | gma-newmiR44 | UGAAGUUACUCUGAGCACUGAG |
| gma-miR166u | UCUCGGACCAGGCUUCAUUC | gma-newmiR45 | ACCCAUCCAACUCUGAAGAUA |
| gma-miR5780 | AUCACUUAGCUGACGGUAGGGAC | gma-newmiR46 | UGUGUUGUUUGUUUUGUAGCA |
| gma-miR5761b | UUUUGUGUCGUGAAGCUUUUG | gma-newmiR47 | UAAUAGAGGGAAGAAGAUGAA |
| gma-miR5033 | GGCUGUACAAAAGGAAACUAC | gma-newmiR48 | UUUAAUAUAUCAGGGACUUGGA |
| gma-miR5765 | CGAAACGUUGAGGUAUAUGUGGAC | gma-newmiR49 | ACAAUUUGGGACUUAGGGCUACAA |
| gma-miR4393b | UUGAAAAGGGACAGCAGAGAAGCC | gma-newmiR50 | AAAGUGUUUGAAUUUCAAUUAGAU |
| gma-miR5772 | AGAAUGUGAGUUAGAGUGAGCAUC | gma-newmiR51 | UGCCAAAGGAGAAUUGCCCUG |
| gma-miR4371a | AAGUGAUGACAUGACAAGCGAAGU | gma-newmiR52 | CACAUCAUACCAUAUUUGCUUC |
| gma-miR5771 | AUCUCAAGUGGAUUGCUUAAGGAC | gma-newmiR53 | UUCAAAUGUCAGAUUAUAAAA |
| gma-miR396g | UUCUUGAACUUCUUAUGCAUC | gma-newmiR54 | AUAUGGACGAAGAGAUAGGUAAAU |
| gma-miR1520a | UAGAACAUGAUACAUGACAGUCA | gma-newmiR55 | UGUUUUGAGUUUCUGAUAAAUU |
| gma-miR396b-5p | UUCCACAGCUUUCUUGAACUU | gma-newmiR56 | AUCAUGCUAUCCCUUUGGAUU |
| gma-miR4387b | AAGGUGUGAUGGCAUGACACUCUG | gma-newmiR57 | CAACCCUCCUCAGUUAGAUCUC |
| gma-miR1520q | AUUGACCAAUCAGAACAUGACACA | gma-newmiR58 | UGCCGGCAAGUUUCUCUUGGC |
| gma-miR4350 | UCAAAUGAUUUUGUGUCGUUGG | gma-newmiR59 | AUACAUAUCGUGUUGCCAAGC |
| gma-miR397b-3p | UAUUGACGCUGCACUCAAUCA | gma-newmiR60 | UUAAUUUUUUUGGAUCAGCAU |
| gma-miR1510a-5p | AGGGAUAGGUAAAACAAUGACUGC | gma-newmiR61 | AGAUAUUGGUACGGUUCAAUC |
| gma-miR395a | CUGAAGUGUUUGGGGGAACUC | gma-newmiR62 | GGAGGCGUAGAUACUCACACC |
| gma-miR4352a | AUUUCUAGGACAUACUACGACGGU | gma-newmiR63 | UGGACUGAAGGGAGCUCCUUC |
| gma-miR4400 | UUCGGAAAAAUUCUGGAAGACGUC | gma-newmiR64 | AAUGGAUAUGAGCUGCAUACA |
| gma-miR4372b | UAAUAAAAUCGUGACAUGUAAC | gma-newmiR65 | UACCCGAAUUUGCUUCCAUGAU |
