# Supplementary material for: Computational prediction of miRNAs and their targets in Phaseolus vulgaris using simple sequence repeat signatures
Source: BMC Plant Biol. 2015 Jun 12;15:140. doi: 10.1186/s12870-015-0516-3 (PMC4464996; doi:10.1186/s12870-015-0516-3)
Supplement: Supplementary file 6 — Predicted targets of P. vulgaris miRNAs. [file 12870_2015_516_MOESM6_ESM.docx]

# **Table S6 - Predicted targets of P. vulgaris miRNAs.**

| Family | Member miRNA(s) | Target protein(s)^a^ | Protein Accession Number^a,b^ | Mode of action^a,c^ |
| --- | --- | --- | --- | --- |
| 156 | miR156a | ankyrin repeat-containing protein | 356542601 | C |
|  | miR156d | MYB transcription factor; serine/threonine-protein kinase; vestitone reductase | 110931858; 356501610; 351723693 | C; C; C |
| 166 | miR166d | UDP-N-acetylglucosamine pyrophosphorylase; SNF1-related protein kinase regulatory subunit | 356524976; 356508802 | C; T |
| 167 | miR167c | quinone-oxidoreductase homolog | 356527330 | C |
| 169 | miR169b | prolycopene isomerase; carboxyl-terminal-processing protease-like | 356522129; 356534629 | C; C |
|  | miR169d | serine/threonine-protein kinase HT1-like | 356521372 | C |
| 171 | miR171a | scarecrow-like protein | 356498250 | C |
| 395 | miR395a | ATP sulfurylase | 90194295 | C |
| 396 | miR396a | succinate dehydrogenase; rhamnose biosynthetic enzyme 1-like | 356538180; 356563232 | C; C |
|  | miR396b | gamma-tubulin complex component 4 homolog | 356500339 | C |
| 408 | miR408a | basic blue protein | 356509342 | C |
| 477 | miR477a | peptide chain release factor GTP-binding subunit ERF3A; glucosyltransferase-8; AAA-metalloprotease FtsH | 356527880; 19911199; 418731182 | C; T; T |
| 482 | miR482a | chlorophyllide a oxygenase; | 42571759 | C; |
| 774 | miR774a | Trans-cinnamate 4-monooxygenase | 586082 | C |
| 848 | miR848a | monosaccharide-sensing protein; lysosomal beta glucosidase; spermidine synthase; translation initiation factor eIF-2B subunit epsilon-like isoform 1; beta-D-xylosidase 7-like; Beta-glucosidase; pectinesterase 53-like; BI1-like protein | 356551132; 356534700; 33340515; 356505908; 356548162; 223545780; 356520820; 356500166 | C; C; C; T; C; C; C; T |
|  | miR848b | PI-PLC X domain; eukaryotic translation initiation factor 3 subunit E; histone-arginine methyltransferase 1.4-like | 356502503; 356500541; 356556084 | C; C; C |
|  | miR848c | Ubiquitin; zinc finger CCCH domain-containing protein 18; | 283245864; 356527087 | C; C; |
|  | miR848d | anthocyanidin reductase-like; ribonuclease Z; NADH dehydrogenase C1; | 356571303; 356501243; 356512870 | C; C; C |
|  | miR848e | pheophorbide a oxygenase; IS10 transposase; PI-PLC X domain-containing protein At5g67130; Ca^2+^-ATPase | 356525253; 113205155; 356502503; 110741169 | C; C; C; C |
|  | miR848g | sorting nexin-1-like; L-ascorbate oxidase homolog; amino acid permease 2 | 356551353; 356496848; 356508941 | C; C; C |
| 861 | miR861a | peroxisome biogenesis protein 12 | 356505983 | C |
| 866 | miR866b | pentatricopeptide repeat-containing protein; axial regulator YABBY 1-like; | 356534396; 356506384 | C; C |
| 902 | miR902b | tetratricopeptide repeat protein 35-A-like | 356505931 | C |
|  | miR902c | fatty acid desaturase 3 | 356526912 | C |
| 1029 | miR1029a | THO complex subunit 4-A-like | 356550261 | C |
| 1043 | miR1043a | ribulose bisphosphate carboxylase/oxygenase activase; histidinol dehydrogenase; arginyl-tRNA--protein transferase 1-like | 356550687; 356532431; 356568461 | C; C; C |
| 1044 | miR1044a | endoplasmin homolog | 356553371 | C |
| 1051 | miR1051a | ceramide kinase-like | 35651033 | C |
| 1134 | miR1134a | small heat shock protein | 356569197 | C |
| 1514 | miR1514a | proteasome subunit alpha type-6-like | 356545355 | C |
| 1527 | miR1527a | lysosomal beta glucosidase-like; cytochrome P450 82C4-like; alpha-galactosidase | 356541600; 356560753; 927577 | C; C; C |
|  | miR1527b | thylakoid membrane phosphoprotein; lysosomal beta glucosidase-like; Anthranilate phosphoribosyltransferase-like; non-specific lipid-transfer protein-like; ferredoxin-NADP reductase | 356506106; 356541600; 357509865; 356570043; 317456382 | C; C; T; C; T |
|  | miR1527c | thylakoid membrane phosphoprotein; lysosomal beta glucosidase-like; non-specific lipid-transfer protein-like; ferredoxin-NADP reductase; transcription factor TCP13-like | 356506106; 356541600; 356570043; 317456382; 356568770 | C; C; C; T; T |
|  | miR1527d | non-specific lipid-transfer protein-like; transcription factor TCP13-like; thylakoid membrane phosphoprotein; | 356570043; 356568770; 356506106 | C; T; C |
|  | miR1527e | non-specific lipid-transfer protein-like; transcription factor TCP13-like; lysosomal beta glucosidase-like | 356570043; 356568770; 356541600 | C; T; C |
|  | miR1527f | lysosomal beta glucosidase-like; alpha-galactosidase | 356541600; 927577 | C; C |
|  | miR1527g | lysosomal beta glucosidase-like; thylakoid membrane phosphoprotein; transcription factor TCP13-like | 356541600; 356506106; 356568770 | C; C; C |
|  | miR1527h | thylakoid membrane phosphoprotein; lysosomal beta glucosidase-like; Anthranilate phosphoribosyltransferase-like protein; non-specific lipid-transfer protein-like; probable inositol transporter 1-like; 60S ribosomal protein L7a-like; | 356506106; 356541600; 357509865; 356570043; 356576905; 356544596 | C; C; C; C; T; T |
|  | miR1527i | non-specific lipid-transfer protein-like; transcription factor TCP13-like; thylakoid membrane phosphoprotein; lysosomal beta glucosidase-like; ferredoxin-NADP reductase | 356570043; 356568770; 356506106; 356541600; 317456382 | C; T; C; C; T |
|  | miR1527j | non-specific lipid-transfer protein-like; transcription factor TCP13-like; thylakoid membrane phosphoprotein; lysosomal beta glucosidase-like; ferredoxin-NADP reductase | 356570043; 356568770; 356506106; 356541600; 317456382 | C; T; C; C; T |
| 1533 | miR1533a | mitogen-activated protein kinase 3-like; plasmalemma intrinsic protein; glucose-6-phosphate 1-epimerase-like isoform 2; E3 ubiquitin-protein ligase RGLG2-like; Non-functional NADPH-dependent codeinone reductase 2; zeta-carotene desaturase precursor; polyubiquitin | 356542571; 886100; 356530310; 356526411; 75266185; 4105563; 899115 | C; T; T; T; T; T; C |
|  | miR1533b | plasmalemma intrinsic protein; glucose-6-phosphate 1-epimerase-like isoform 2; E3 ubiquitin-protein ligase RGLG2-like; Non-functional NADPH-dependent codeinone reductase 2; zeta-carotene desaturase precursor; syntaxin-24-like; adenine phosphoribosyltransferase; | 886100; 356530310; 356526411; 75266185; 4105563; 356505422; 356514471 | C; C; C; C; C; C; C; |
|  | miR1533c | MLP-like protein; defender against cell death 1-like; 7s Globulin; cytochrome P450 78A3-like; nodulin 41; indole-3-acetic acid-amido synthetase GH3.5-like; legumin; phototropin; | 413968364; 356497637; 330689365; 356559861; 343198386; 356560454; 312982406; 60099456 | C; C; C; C; C; C; C; C |
|  | miR1533d | MLP-like protein; Plant Photosystem I; defender against cell death 1-like; proteolipid subunit of vacuolar H+ ATPase; 7s Globulin; protein kinase family protein; UDP-glycosyltransferase 91B1-like | 413968364; 268612174; 356497637; 168542; 330689365; 351727579; 356503182 | C; C; C; C; C; C; C; |
|  | miR1533e | adenine phosphoribosyltransferase 1; E3 ubiquitin-protein ligase RGLG2-like; Non-functional NADPH-dependent codeinone reductase 2; zeta-carotene desaturase precursor; syntaxin-24-like; glucose-6-phosphate 1-epimerase-like isoform 2; c-Myc-binding protein-like; | 356514471; 356526411; 75266185; 4105563; 356505422; 356530310; 356506005 | C; C; C; C; C; C; C; |
|  | miR1533f | mitogen-activated protein kinase 3-like; plasmalemma intrinsic protein; glucose-6-phosphate 1-epimerase-like isoform 2; E3 ubiquitin-protein ligase RGLG2-like; Non-functional NADPH-dependent codeinone reductase 2; zeta-carotene desaturase precursor; metallothionein type 2; syntaxin-24-like; | 356542571; 886100; 356530310; 356526411; 75266185; 4105563; 146747422; 356505422 | C; C; T; T; T; T; C; T |
|  | miR1533g | WW domain-containing oxidoreductase-like; Thylakoid formation 1 chloroplastic-like; 7s Globulin; MLP-like protein | 356529180; 356543780; 330689365; 413968364 | C; C; T; T; |
|  | miR1533h | Thylakoid formation 1 chloroplastic-like; peptide/nitrate transporter; serine/threonine-protein phosphatase 4 regulatory subunit 3-like isoform 2 | 356543780; 356569101; 356521793 | T; C; C; |
|  | miR1533i | metallothionein type 2; mitogen-activated protein kinase 3-like; plasmalemma intrinsic protein; transmembrane protein 18-like; mitogen-activated protein kinase 3-like; glucose-6-phosphate 1-epimerase-like isoform 2; E3 ubiquitin-protein ligase RGLG2-like; Non-functional NADPH-dependent codeinone reductase 2; arogenate dehydratase/prephenate dehydratase 6; | 146747422; 356542571; 886100; 356542571; 356530310; 356526411; 75266185; 356543548 | C; C; T; C; C; T; T; T; C; |
|  | miR1533j | MLP-like protein; 7s Globulin; pleckstrin homology domain; aspartate aminotransferase 2; oxidation resistance protein 1-like; cytochrome P450 78A3-like; | 413968364; 330689365; 356576513; 366984548; 356535454; 356559861 | C; C; C; C; C; C; C; |
|  | miR1533k | epoxide hydrolase 2-like; aspartic proteinase-like protein 2-like; histone H4; GPN-loop GTPase 2-like; MKI67 FHA domain-interacting nucleolar phosphoprotein-like; L-ascorbate peroxidase T; MLP-like protein; pentatricopeptide repeat-containing protein At5g59600-like; | 35656424; 356564743; 347597786; 356518134; 356530697; 356509393; 413968364; 356541211 | C; C; C; C; C; C; C; C |
|  | miR1533l | cytochrome P450 78A3-like; nodulin 41; indole-3-acetic acid-amido synthetase GH3.5-like; legumin; phototropin; | 356559861; 343198386; 356560454; 312982406; 60099456 | C; C; C; C; C; |
|  | miR1533m | pseudouridine-5'-monophosphatase-like | 356543908 | C |
|  | miR1533n | oxidation resistance protein 1-like; cytochrome P450 78A3-like; nodulin 41; indole-3-acetic acid-amido synthetase GH3.5-like; legumin; phototropin | 356535454; 356559861; 343198386; 356560454; 312982406; 60099456 | C; C; C; C; C; C; |
|  | miR1533o | oxidation resistance protein 1-like | 356535454 | T |
| 1888 | miR1888a | U-box domain-containing protein 7-like | 356540164 | C |
| 2095 | miR2095a | 26S proteasome ATPase subunit | 50660432 | C |
| 2673 | miR2673a | U6 snRNA-associated Sm-like protein; apoptosis inhibitor 5-like | 356572732; 356521430 | C; C |
|  | miR2673b | triose phosphate/phosphate translocator; THO complex subunit 4-A-like; squamosa promoter-binding-like; photosystem II stability/assembly factor HCF136; asparagine synthetase type II; F-box protein SKIP5; Membrane protein sll0875-like; cytochrome P450 94A1-like; putative septum site-determining protein minD homolog; asparagine synthetase type II | 356564996; 356550261; 356503476; 357117071; 3821280; 355487680; 356544132; 356570622; 356529149; 3821280 | C; C; C; C; C; C; C; C; C; C |
| 3442 | miR3442a | epoxide hydrolase 2-like | 356564247 | C |
| 3979 | miR3979 | protein FRIGIDA-like; vacuolar-sorting receptor 1-like isoform 1; FAD-linked oxidoreductase 2 precursor ;beta-carotene hydroxylase; sphingoid long-chain bases kinase 1-like; Plant Photosystem I; histone H2A.5 | 356504458; 356536097; 351721585; 342357374; 356573377; 303324975; 356572998 | C; C; C; C; C; C; C |
| 5021 | miR5021a | fiber protein Fb23; heat stress transcription factor C-1-like; glycylpeptide N-tetradecanoyltransferase 1-like isoform 2; plastid-lipid-associated protein 4; chloroplast chlorophyll a/b-binding protein; septum site-determining protein minD homolog | 30983938; 356531261; 356522160; 356558503; 117661756; 356529149; | C; C; C; C; C; C; |
|  | miR5021b | CPRD86; vacuolar protein sorting-associated protein 4B-like; MLP-like protein; HSF domain class transcription factor; dolichyl-diphosphooligosaccharide; nuclease HARBI1-like | 9857292; 356542738; 413968364; 302398883; 356517213; 356502730 | C; C; C; C; C; C |
|  | miR5021c | class II KNOX protein; probable plastid-lipid-associated protein 4; ferritin; RPM1-interacting protein 4; asparagine synthetase type II; F2K11.3; T-complex protein 1 subunit epsilon-like | 371767706; 356558503; 21027; 356527157; 3821280; 6633836; 356567490 | C; C; C; C; C; C |
|  | miR5021d | phospholipase A(1) LCAT3-like; ornithine aminotransferase; CPRD86; ribulose bisphosphate carboxylase/oxygenase activase, chloroplastic-like | 356568525; 351727128; 9857292; 356550687 | C; C; C |
|  | miR5021e | plastid-lipid-associated protein 4; ferritin; RPM1-interacting protein 4; asparagine synthetase type II; F2K11.3; T-complex protein 1 subunit epsilon-like | 356558503; 21027; 356527157; 3821280; 6633836; 356567490 | C; C; C; C; C; C |
|  | miR5021f | class II KNOX protein; RPM1-interacting protein 4; glutaredoxin-C5; asparagine synthetase type II ; homeobox; RPM1-interacting protein 4 | 371767706; 356527157; 225437910; 3821280; 356530621; 356527157 | C; C; C; C; C; C |
|  | miR5021g | class II KNOX protein; RPM1-interacting protein 4; asparagine synthetase type II ; homeobox protein; RPM1-interacting protein 4; enolase 1 | 37176770; 356527157; 3821280; 356530621; 356527157; 356519186 | C; C; C; C; C; C |
|  | miR5021h | CPRD86; ribulose bisphosphate carboxylase/oxygenase activase; Mo25 family protein; phospholipase A(1) LCAT3-like ; nuclease HARBI1-like; ATP-dependent Clp protease proteolytic subunit 3 | 9857292; 356550687; 35748055; 356568525; 356502730; 356569022 | C; C; C; C; C |
|  | miR5021i | vacuolar protein sorting-associated protein 4B-like; Oligopeptidase A;HSF domain class transcription factor; dolichyl-diphosphooligosaccharide; tRNA pseudouridine synthase A-like; DEAD-box ATP-dependent RNA helicase 5-like; dual specificity protein kinase pyk1-like; fasciclin-like arabinogalactan | 356542738; 357485331; 302398883; 356517213; 356543399;356531475; 356521649; 356527155 | C; C; C; C; C; C; C; C |
|  | miR5021j | vacuolar protein sorting-associated protein 4B-like; HSF domain class transcription factor; dolichyl-diphosphooligosaccharide; DEAD-box ATP-dependent RNA helicase 5-like | 356542738; 302398883; 356517213; 356531475 | C; C; C; C |
| 5041 | miR5041a | Galactinol-sucrose galactosyltransferase | 357461865 | T |
| 5054 | miR5054b | Ty-1 copia retrotransposon | 353685482 | C |
| 5140 | miR5140a | peroxisomal ascorbate peroxidase; conserved oligomeric Golgi complex subunit 3-like; cytochrome P450 94A1-like; pyrrolidone-carboxylate peptidase-like isoform 2; NADP-dependent glyceraldehyde-3-phosphate dehydrogenase-like | 167963366; 356565620;356503586; 356505751; 356562585 | C; C; C; C; C |
| 5177 | miR5177a | endoplasmin homolog | 356553371 | C |
| 5248 | miR5248a | TMV resistance protein N-like | 356506581 | C |
| 5368 | miR5368a | Cell wall-associated hydrolase | 355504652 | C |
|  | miR5368b | Cell wall-associated hydrolase | 355505211 | C |
| 5558 | miR5558a | transmembrane protein 184B-like | 356575269 | C |
| 5562 | miR5562a | 40S ribosomal protein S17E | 313586441 | C |
| 5654 | miR5654a | Ted2; UTP--glucose-1-phosphate uridylyltransferase-like | 1617036; 356516358 | C; C |
|  | miR5654b | Eukaryoticelongation initiation factor 4A-11-like; UDP-glycosyltransferase 92A1-like; transmembrane protein 115-like; serine/threonine protein kinase-like | 386870481; 356557419; 356498564; 351722951 | C; C; C; C |
| 5721 | miR5721b | SUN domain-containing protein 1-like; fructokinase-1-like | 356556739; 356545828 | C; C |
| 5773 | miR5773a | lysosomal beta glucosidase-like;cytochrome P450 82C4-like ; alpha-galactosidase | 356541600; 356560753 | C; C; C |
| 5998 | miR5998a | arginase-like; ferredoxin NADP+ reductase protein | 356496368; 374255993 | C; T |
|  | miR5998b | serine/threonine-protein kinase Cx32; serine/threonine-protein kinase STN7; V-type proton ATPase subunit C-like; 1-acyl-sn-glycerol-3-phosphate acyltransferase 4-like; arginase-like; reticulon-like protein B2; S-adenosylmethionine decarboxylase | 356501765; 356495992; 356498582; 356564243; 356496368; 356513327; 37694878 | C; C; C; C; C; C; C |
| 6034 | miR6034a | protein strubbelig-receptor family 3-like ; inactive leucine-rich repeat receptor-like; serine/threonine-protein kinase ; phytosulfokine receptor 2-like | 356547026; 356544420; 356509845; 356535758 | C; C; C; C |
|  | miR6034b | serine/threonine-protein kinase | 356548711 | C |
|  | miR6034c | protein kinase | 223452329 | C |
|  | miR6034d | serine/threonine-protein kinase ; transmembrane protein 53-like | 356548711; 356574115 | C; C |
| 6169 | miR6169a | cytochrome P450 710A1-like; ribosomal protein L19 | 356555538; 38324697 | C |
|  | miR6169b | gibberellin receptor; coatomer subunit beta-1-like; phosphoinositide 3-kinase | 238654635; 356521768; 35649757 | C; C; C |
| 6196 | miR6196a | Fumarylacetoacetase-like; tubulin beta-1 chain-like; leucine-rich repeat extension-like | 149941260; 356535873; 356575879 | C; T; C |
| 6214 | miR6214a | amino acid permease YfnA-like; serine incorporator-like | 356532335; 356546444 | C; C |
| 6470 | miR6470b | alpha-galactosidase | 927577 | C |
